# Human microphysiological systems of aging recreate the in vivo process expediting evaluation of anti-geronic strategies

**DOI:** 10.1038/s41551-026-01618-6

**Published:** 2026-03-25

**Authors:** Lin Qi, Yuchen He, Alexandra Sviercovich, Xiaoyue Mei, Erzhen Chen, Yihan Xia, Michael J. Conboy, Irina M. Conboy, Andreas Stahl

**Affiliations:** 1 Department of Nutritional Science and Toxicology, College of Natural Resources, University of California Berkeley, Berkeley, CA USA; 2 Department of Bioengineering and QB3 Institute, University of California Berkeley, Berkeley, CA USA; 3 Generation Lab, San Francisco, CA USA

**Keywords:** Lab-on-a-chip, Ageing, Metabolic diseases, Stem-cell biotechnology, Experimental models of disease

## Abstract

The search for biological mechanisms of human aging is stalled by a lack of suitable models, and it remains unknown whether and to what degree rejuvenation reported in rodents translates to people. Here we report a human induced pluripotent stem cell-derived microphysiological system modelling the white adipose tissue–liver axis in the presence of heterochronic human serum to study aging and rejuvenation in humans. We reveal changes in functional and molecular hallmarks of aging and rejuvenation. We also investigate unknown biomarkers and mechanisms of plasticity in human tissue aging and potential rejuvenation strategies. The microphysiological chip recapitulates, in 4 days, aging-associated hallmarks that occur after decades of aging in people, including gerontic shifts in gene expression and oxidative DNA damage. We uncover unknown signalling networks in human aging, knock-on effects of aging in fat on liver, sexual polymorphisms of aging and tissue memory of age, and develop a custom machine learning model for biological age. Combining heterochronic human serum with the microphysiological system allows for rapidly establishing human tissue aging, discovering clinically relevant mechanisms and biomarkers, and testing of anti-geronic approaches.

## Abstract (web summary)

Heterochronic human serum in a microphysiological system of hiPSC-derived white adipose tissue–liver axis allows the study of aging and rejuvenation in humans.

## Main

One billion people are age 60 and older, equating to 10% of the global population, a number that is estimated to reach 22% by 2050, and is already creating urgent medical and social challenges worldwide^1^. White adipose tissue (WAT) shows an early onset of aging^2,3^ that contributes to systemic metabolic disorders^4–7^, including those of liver, which is directly exposed to adipokines from visceral fat via the portal vein^8–10^. Thus, age-related changes in the WAT–liver axis, which can manifest by middle age, need to be better understood for developing therapeutics that attenuate the progression of systemic aging and a plethora of age-associated diseases.

Currently, both human clinical studies and rodent models of aging are highly protracted except for heterochronic blood exchange in mice^11–13^, an invasive procedure that cannot be directly translated to studying human aging. Considering the inconsistencies across different species^14–17^, human in vitro models are more clinically relevant for studying key mechanisms in heterochronic blood exchange but face challenges in regard to maintaining the viability and functionality of hepatocytes^18,19^ and adipocytes^20^, and frequently lack the ability to reproduce interorgan crosstalk. Primary human cell-based systems further suffer from issues of reproducibility, scalability and limited approaches for genetic manipulation. Human induced pluripotent stem cell (hiPSC)-based approaches can overcome these limitations, but functional techniques to generate functional iPSC-derived adipocytes and the ability to interconnect them to hepatocytes were only recently developed^21–23^.

Here we investigate the effects of blood heterochronicity on a human iPSC-derived WAT–liver-axis microphysiological system (MPS), a preclinical animal system alternative recently developed by us^23^ as advocated by the FDA Modernization Act 2.0 (ref. ^24^). Our miniaturized in vitro model of functional human tissues substantially reduces the needed volumes of serum, which is often limited from older donors^25^, by 2–3 orders of magnitude. We found that hallmarks of aging are established in 4 days by human serum from old (>62 years) donors, accurately representing human aging that takes decades in vivo. In addition, the interconnected WAT–liver MPS allows for continuous medium infusion and the ability to simulate interorgan crosstalk. The propagation of aging from fat to liver and mechanisms behind the plasticity of human aging were uncovered, enhancing the understanding of this fundamental process through the new approach of human on-chip aging.

## Results

### Aging of WAT MPS and liver MPS by human sera and pro-geronic effects of aged fat on liver

**Fig. 1:**
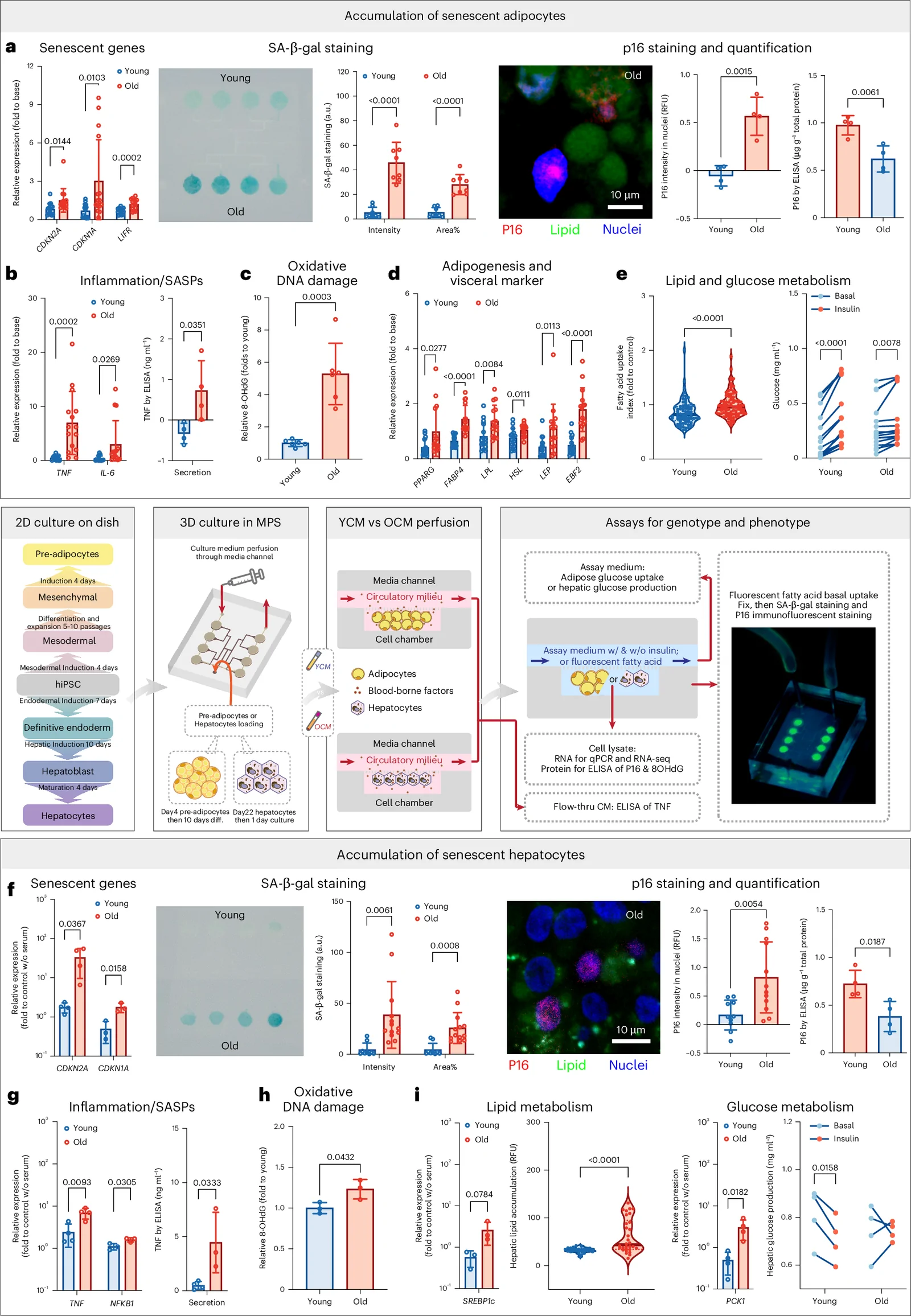
Human serum induced aging in WAT and liver MPS. **a**–**i**, After 4 days of perfusion of the WAT MPS (**a**–**e**) and liver MPS (**f**–**i**) with OCM vs YCM, several clinically relevant aging-associated tissue dysfunctions were established. **a**,**f**, Accumulation of senescent cells in measures of aging-associated gene expression (*n* = 15 in all genes in **a**; *n* = 4 for *CDKN2A*, *n* = 3 for *CDKN1A* in **f**); SA-β-gal senescence (*n* = 8 for both in **a**; *n* = 9 for YCM and *n* = 13 for OCM in **f**); increase in p16 marker of senescence (*n* = 4 for all in **a**; *n* = 10 for YCM and *n* = 12 for OCM in nuclei-positive p16, and *n* = 4 for both in p16 ELISA in **f**). **b**,**g**, Inflammatory proteins and senescence-associated secretory phenotype, SASP. *n* = 15 for all genes and *n* = 4 for TNF ELISA in **b**; *n* = 4 for both in *TNF*, *n* = 3 for both in *NFKB1*, *n* = 4 in YCM and *n* = 3 in OCM for TNF by ELISA in **g**. **c**,**h**, Increase in oxidative DNA damage. *n* = 6 for both in **c** and *n* = 3 for both in **h**. **d**, Perturbation of adipogenesis and fat depot. *n* = 15 for all. **e**,**i**, Perturbation of lipid and insulin-regulated glucose metabolism. *n* = 77 in YCM and *n* = 93 for OCM in lipid metabolism; *n* = 15 for both of YCM and *n* = 16 for both of OCM in glucose metabolism in **e**; *n* = 3 for both in *SREBP1c*, *n* = 4 for both in *PCK1*, *n* = 29 in YCM and *n* = 30 for OCM in lipid metabolism; *n* = 4 for all in glucose metabolism in **i**. *n* refers to one chamber on MPS in quantifications of SA-β-gal staining, P16 nuclei-positive fluorescence and lipid metabolism; *n* refers to media in TNF by ELISA and in glucose metabolism; otherwise, *n* refers to MPS. *P* values were calculated using paired two-tailed *t*-test for the comparison of basal vs insulin in glucose metabolism, unpaired two-tailed *t*-tests for the other comparisons. Exact *P* < 0.1 are directly noted on the compared pairs. Data are presented as mean ± s.d. for all bar graphs. All gene markers were normalized to a negative control, which was cultured in the same batch with the conventional MPS medium. TNF secretion was assessed relative to a serum-free control condition, with negative values indicating comparatively reduced production. Statistical methods and exact *P* values are shown in Supplementary Data 1. Only significant gene pairs are shown, and all tested genes are shown in Supplementary Fig. 1. Source data

hiPSC-derived MPSs (iADIPO and iHEP MPS, also simplified as WAT and liver MPS) were perfused for 4 days with human plasma-like medium (HPLM) containing 5% serum from old male or female human donors (>62 years), which we term old circulatory milieu (OCM). Alternatively, MPSs were exposed to serum from sex-matched young donors (21–34 years) to establish a young circulatory milieu (YCM; see Methods for details). We assessed multiple hallmarks of aging, including cellular senescence, oxidative DNA damage, inflammatory gene expression, as well as changes in the expression of the aging-associated adipocyte progenitor marker *LIFR*^26^, and several key molecular and functional features of WAT and liver metabolism. Results demonstrate that OCM quickly and robustly established multiple aging phenotypes in WAT and liver MPS, both general and tissue specific (Fig. 1).

With respect to the general hallmarks of aging, human tissue senescence was increased by OCM based on senescence-associated β-galactosidase (SA-β-gal) staining, expression of *CDKN1A* and *CDKN2A*, as well as the increased nucleus-localized and total protein levels of p16 as determined by immunofluorescence intensity and quantification by ELISA (Fig. 1a,f). We detected higher levels of senescence-associated secretory protein (SASP, *TNF*, *IL-6*) expression and secretion (Fig. 1b,g). Oxidative DNA damage, measured by 8-OHdG assay, was also robustly induced by OCM in the WAT MPS, with a similar albeit weaker trend observed in the liver MPS (Fig. 1c,h).

Next, we examined adipose and hepatic metabolic markers and functions. Compared to YCM, OCM induced markers of adipogenesis and adipose lipid metabolism (*PPARG*, *FABP4*, *LPL*, *HSL*), as well as obesity/aging-related adipokines (*LEP*) and increased visceral fat depot signatures, that is, higher *EBF2* (Fig. 1d). Furthermore, OCM not only increased lipid synthesis but also elevated the uptake of exogenous fatty acids by the WAT and liver MPSs (Fig. 1e,i). In liver MPS, OCM induced a trend for elevated expression of the lipogenic master regulator *SREBP1c* (0.05 < *P* < 0.1; Fig. 1i).

In addition to lipid metabolism, glucose metabolism was also perturbed by OCM, indicative of an insulin-resistant state: insulin-regulated hepatic gluconeogenesis marker *PCK1* was increased, insulin-induced adipose glucose uptake and regulation of hepatic glucose production were both impaired (Fig. 1e,i). Of note, when compared to the serum-free conventional MPS medium, YCM and OCM drive WAT MPS genes (*CDKN2A*, *CDKN1A*, *LIFR*, *TNF*, *IL6*, *FABP4*, *LPL*, *HSL*, *EBF2)* and TNF secretion, as well as liver-MPS genes (*CDKN1A*, *SREBP1c*, *PCK1*) in opposite directions (Fig. 1).

**Extended Data Fig. 1:**
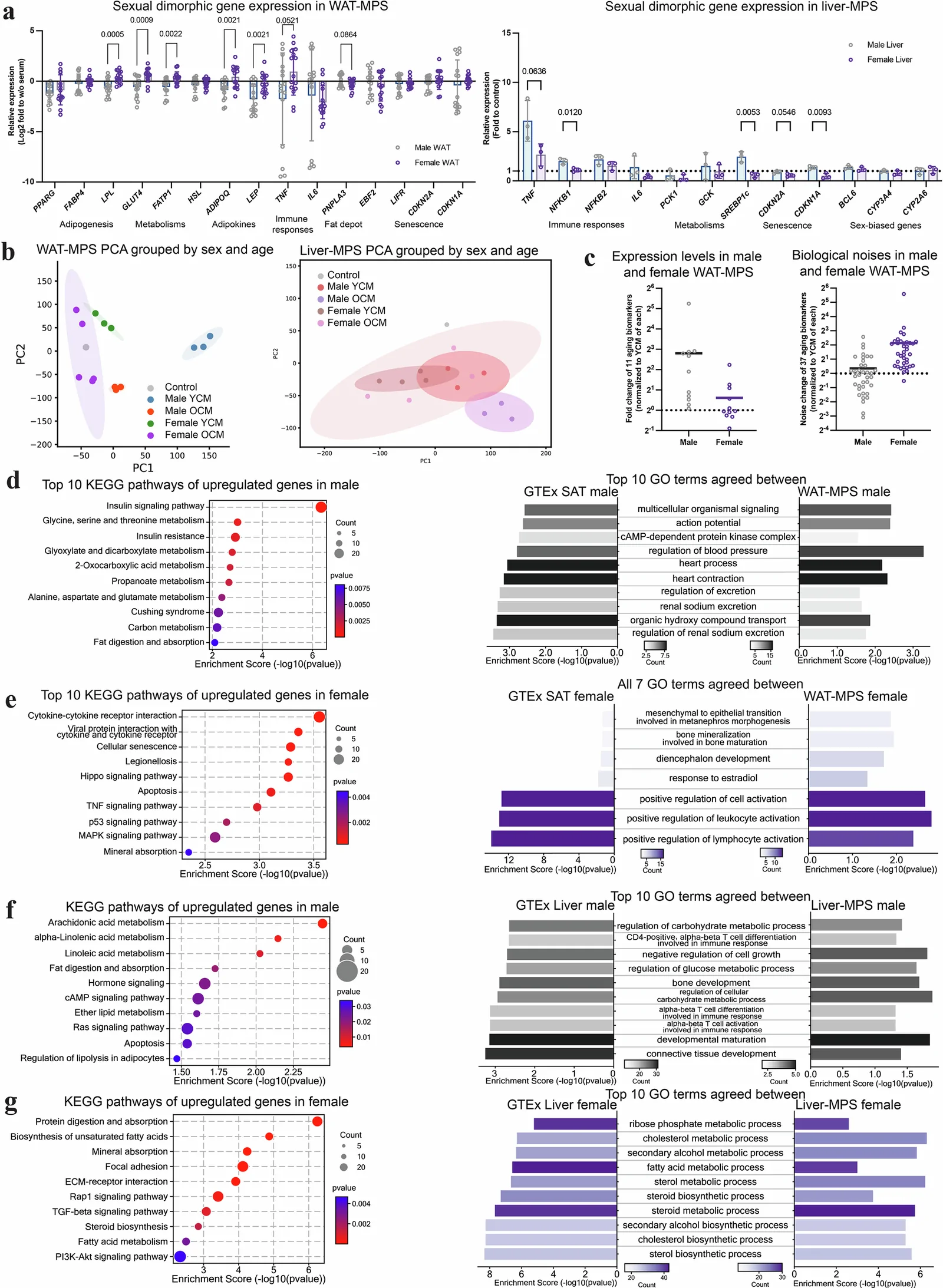
Sexual dimorphism in serum-treated MPSs. (**a**) gene expression characterized by qPCR in WAT- and liver-MPS, replotting from the same data set of Fig. 1 but in the measure of sex. n = 15 for all in WAT-MPS and n = 3 for all in liver MPS. n referring to MPS. p-values were calculated by unpaired two-tailed t-tests. Exact p values < 0.1 were directly noted on the compared pairs. Data are presented as mean values +/- SD. All genes were normalized to serum-free control. (**b**) Principal components analysis regarding on both age and sex in WAT-MPS and liver-MPS. (**c**) Aging biomarkers of expression level and biological noises in male and female conditions of WAT-MPS. (**d**,**e**) Top 10 KEGG pathways of upregulated genes in male (**d**) and female (**e**), and GO terms overlapped to GTEx subcutaneous adipose tissue database. (**f**,**g**) Signature KEGG pathways of upregulated genes in male (**f**) and female (**g**), and GO terms overlapped to GTEx liver database, all in their young conditions due to diminished sex dimorphism in old. Source data

Importantly, both YCM and OCM also established dimorphic patterns based on the sex of serum donors (Extended Data Fig. 1a). In the WAT MPS, this included differential gene expression for adipogenesis markers (*LPL*, *GLUT4*, *FATP1*, *ADIPOQ*, *LEP*), adipokines (*ADIPOQ*, *LEP*), insulin-sensitive glucose and lipid metabolism (*GLUT4* and *FATP1*), as well as trends for sex-based differences in inflammation and subcutaneous adipogenesis. Similarly, liver MPSs exposed to male YCM exhibited higher inflammation, lipogenesis and senescence compared with MPSs exposed to female YCM.

**Fig. 2:**
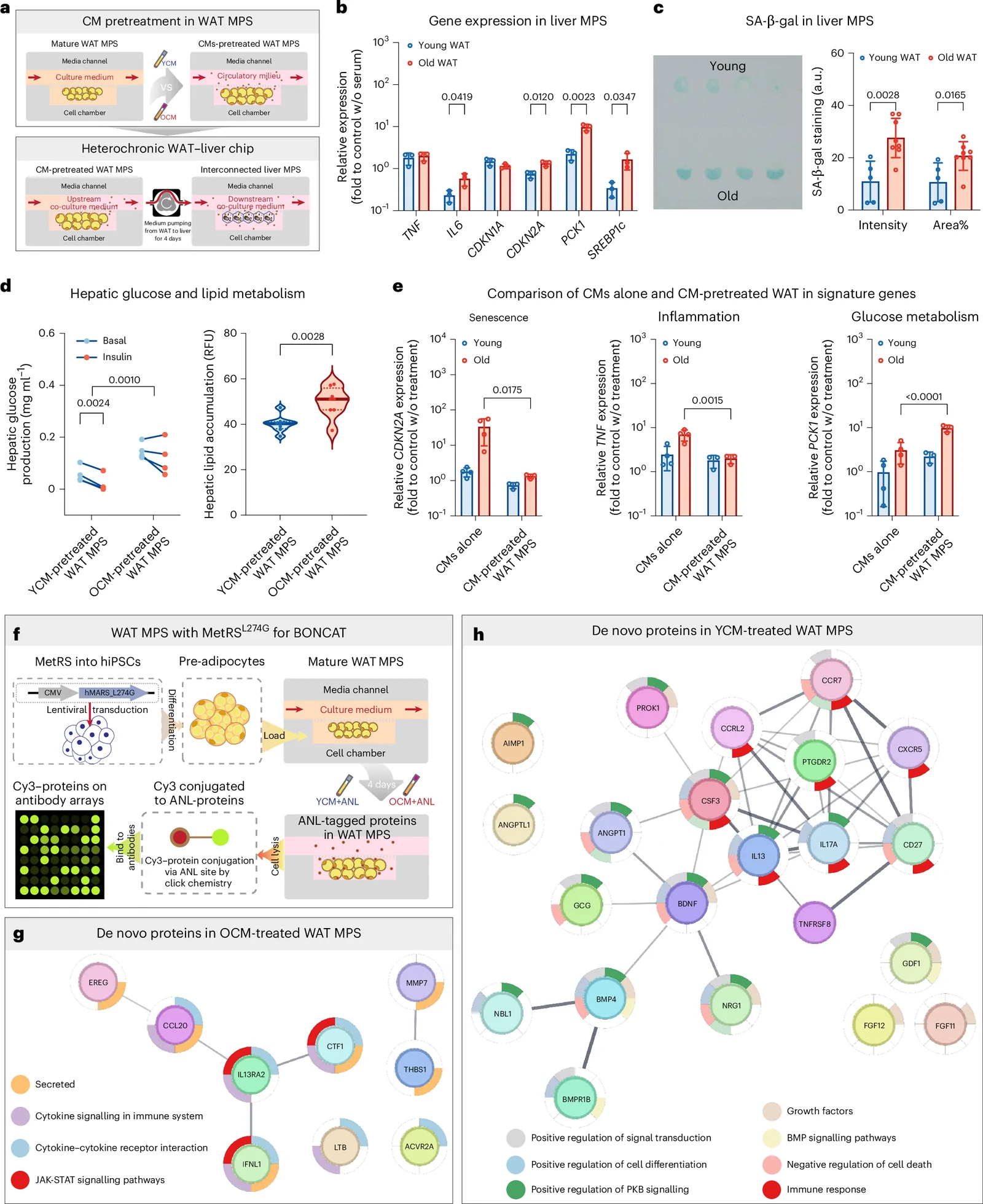
Aged fat propagates aging to liver MPS. Transfer of aging from OCM-pretreated fat to liver was successfully established in the WAT–liver organome chip (**a**, schematics), with respect to the altered gene expression (**b**). *n* = 3 for all genes in **b**. **c**, Induction of SA-β-gal accumulation. *n* = 5 in YCM and *n* = 8 in OCM. **d**, Perturbation of glucose and lipid metabolism. *n* = 4 for all glucose and *n* = 8 for all lipid metabolism. **e**, Genotype pattern comparing to OCM treatment in liver. *n* = 4 for all CM alone and *n* = 3 for all CM pretreatments. All gene markers were normalized to the negative control, which was cultured in the same batch with the conventional MPS medium. *n* refers to one chamber in MPS in quantifications of SA-β-gal staining and lipid metabolism; *n* refers to media in glucose metabolism; otherwise, *n* refers to MPS. *P* values were calculated using paired two-tailed *t*-test for the comparison of basal vs insulin in glucose metabolism, and unpaired two-tailed *t*-tests for the other comparisons of young vs old; using two-way ANOVA for YCM- vs OCM-pretreated conditions of glucose metabolism (**d**); and using two-way ANOVA followed by Šídák’s test for cross-group difference (**e**). Exact *P* < 0.1 are directly noted on the compared pairs. Data are presented as mean ± s.d. for all bar graphs. Statistical methods and exact *P* values are shown in Supplementary Data 1. Source data are provided. **f**, Workflow schematic for the detection of de novo proteins in WAT MPSs derived from MetRS^L274G^ hiPSCs. **g**,**h**, Profiles of de novo proteins under OCM (**g**) and YCM (**h**) conditions. Proteins are shown as their encoding gene names in the enrichment analysis. Data represent merged results from both male and female samples. A singleton without enriched functions is not shown. Source data

Aging-induced dysfunction in visceral adipose tissue adipokine and metabolite secretion directly impacts the liver via the portal vein. Here we recreated this process by first establishing a cellular memory of exposure to YCM or OCM in WAT MPS and then interconnected the fat chips with untreated liver MPS via a unidirectional perfusion from the WAT to the liver with the basal HPLM medium (Fig. 2a). The data demonstrated that the OCM- but not the YCM-pretreated WAT MPS quickly induced aging hallmarks in the liver MPS, including higher expression of *IL6*, *CDKN2A*, *PCK1* and *SREBP1c* (Fig. 2b), senescence (Fig. 2c) and insulin resistance indicating elevated hepatic glucose production and higher lipid accumulation (Fig. 2d). Interestingly, this knock-on hepatocyte dysfunction differed from the OCM-driven aging phenotype of liver MPS based on key signature genes of senescence, inflammation and glucose metabolism (Fig. 2e).

To further dissect the proteomic drivers of these on-chip aging patterns, we employed WAT MPSs derived from the hiPSC line stably expressing the methionyl-tRNA synthetase mutant (MetRS^L274G^) for bio-orthogonal non-canonical amino acid tagging (BONCAT) (Fig. 2f). This system enables incorporation of azidonorleucine (ANL) into newly synthesized proteins, which can then be detected via click chemistry on the azide group^27–30^. Using this approach, we found that OCM rapidly promoted de novo protein synthesis along activation of inflammatory signalling pathways (Fig. 2g). Conversely, YCM induced the groups of newly synthesized growth factors that promote cell proliferation, developmental pathways and core homeostatic biological processes (Fig. 2h). Notably, the changes in proteomic output under OCM also mirrors the broadly observed age-associated decline in gene expression reported in natural aging and parabiosis studies^31^.

Taken together, these results establish a rapid and sex-specific in vitro system of aging using human iPSC-derived adipocytes and hepatocytes that, within 4 days of OCM treatment, show key features of aging including senescence with secretion of inflammatory/SASP markers, oxidative DNA damage, blunted insulin sensitivity with dysregulated glucose and lipid metabolism. Further, OCM exposure established a cellular memory that propagated aging-related dysfunctions from one tissue to another even in the absence of continued exposure to OCM.

### On-chip aging closely recreates natural human aging based on genome-wide transcriptomics

**Fig. 3:**
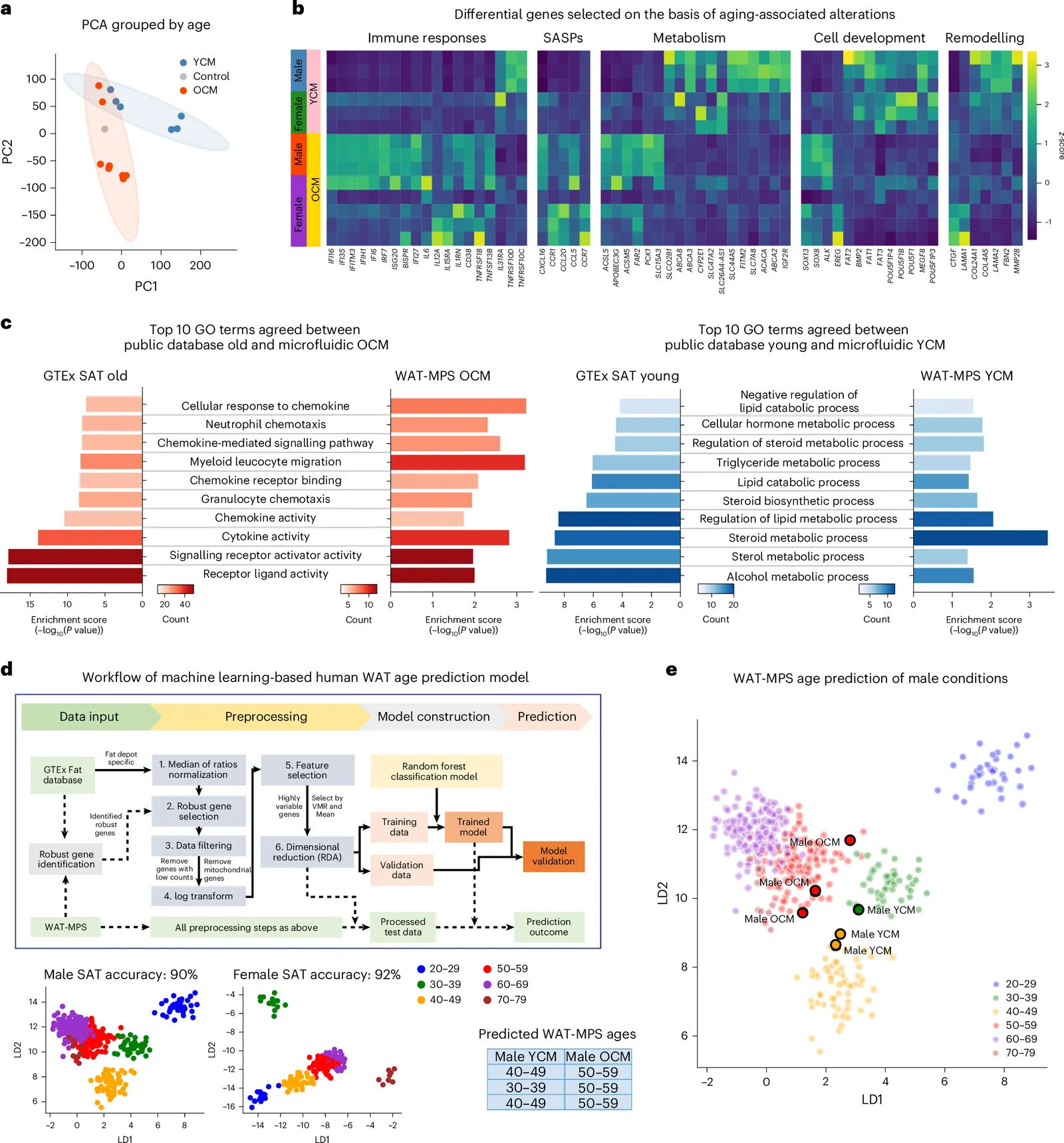
Transcriptomics of the serum-treated heterochronic WAT MPS and human data-trained ML model predict the age of WAT MPS. **a**, PCA grouped by age conditions. *n* = 3 for young-male, young-female and old-male conditions, *n* = 5 for old-female condition. **b**, Genes of aging-associated alterations selected from the top 25% of the differential gene list. **c**, Top 10 GO terms agreed between the public database (GTEx subcutaneous adipose tissue, SAT) and WAT MPSs with OCM and YCM treatments. **d**, Human SAT age prediction model training workflow based on machine learning from the GTEx database. The model was trained on the basis of 70% random datapoints of GTEx, and then validated using the remaining 30% of datapoints. **e**, Age prediction of male OCM- and YCM-treated WAT MPS. Source data

**Extended Data Fig. 2:**
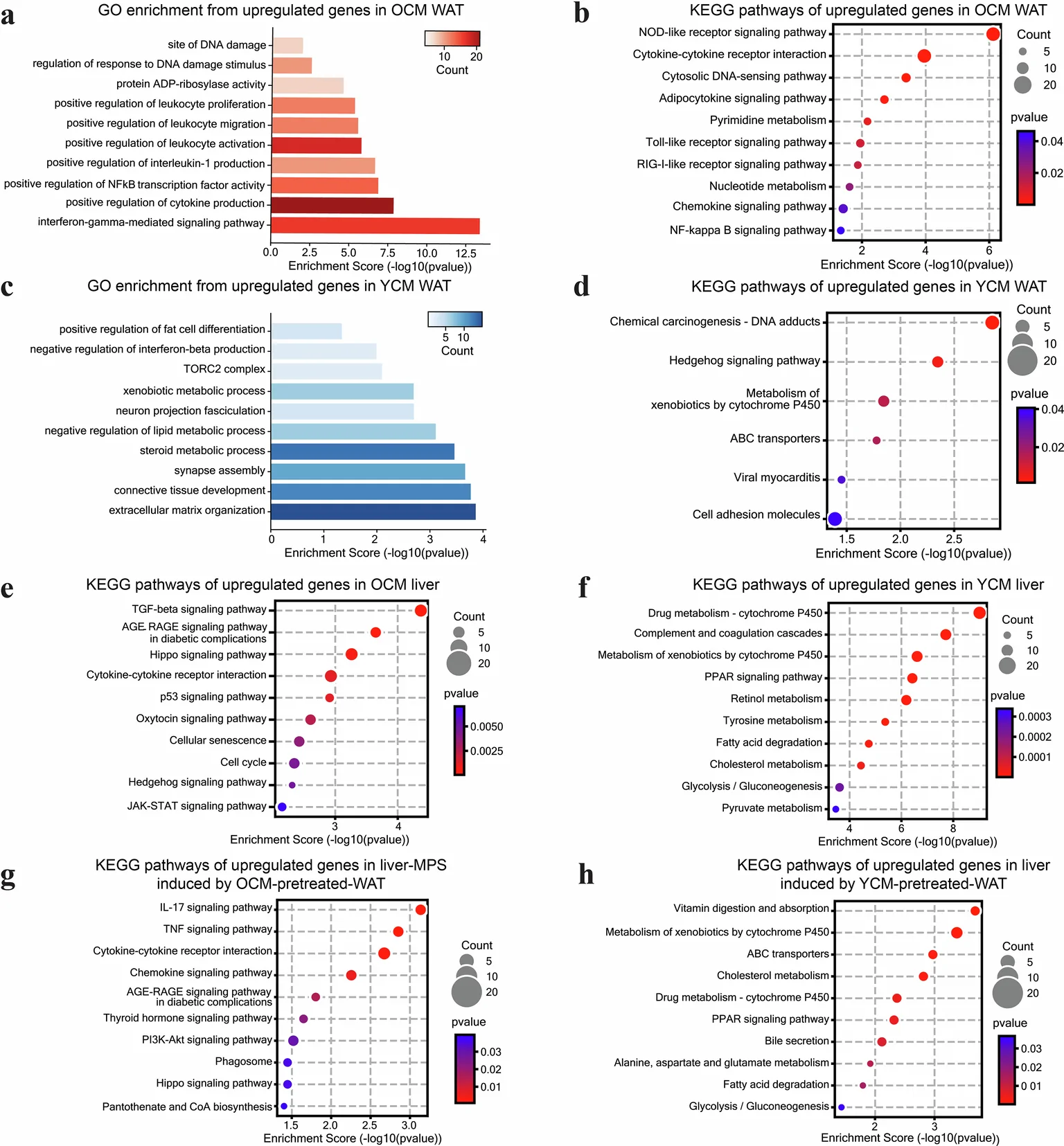
Additional analyses of serum heterochronicity in WAT-/liver-MPS and fat-liver organome influences. (**a**,**c**) GO and (**b**,**d**) KEGG enriched from WAT-MPS under OCM (**c**,**d**) and YCM (**e**,**f**) conditions. (**e**,**f**) KEGG enriched liver-MPS under OCM (**e**) an YCM (**f**) conditions. (**g**,**h**) KEGG in liver-MPS induced by OCM-pretreated (**g**) and YCM-pretreated WAT-MPS (**h**).

Bulk RNA-sequencing analysis was performed on the 4-day serum-treated WAT MPS. Principal component analysis (PCA; Fig. 3a) distinguished two signature clusters grouped by YCM and OCM. Differential analysis demonstrated that the on-chip heterochronicity of human serum influenced biomedically relevant and known age-specific gene expression patterns: immune-tissue crosstalks and regulators of inflammation (*TNF*, interleukins, chemokines, interferons, their receptors and associated proteins), metabolism (adipokine signalling pathways, *SLC*s, *PCK1*, *CYP2E1*, *ACC*s), the aging hallmarks of ADP-ribosylase activity, cytosolic DNA sensing, DNA damage, lifespan-relevant TORC2, regulators of cell fate and cell proliferation (*SOX*s, *POU5F*s, *FAT*s) and tissue remodelling factors (*COL*s, *LAMA*s, *MMP28*, *FBN2*) (Fig. 3b and Extended Data Fig. 2a,b).

**Extended Data Fig. 3:**
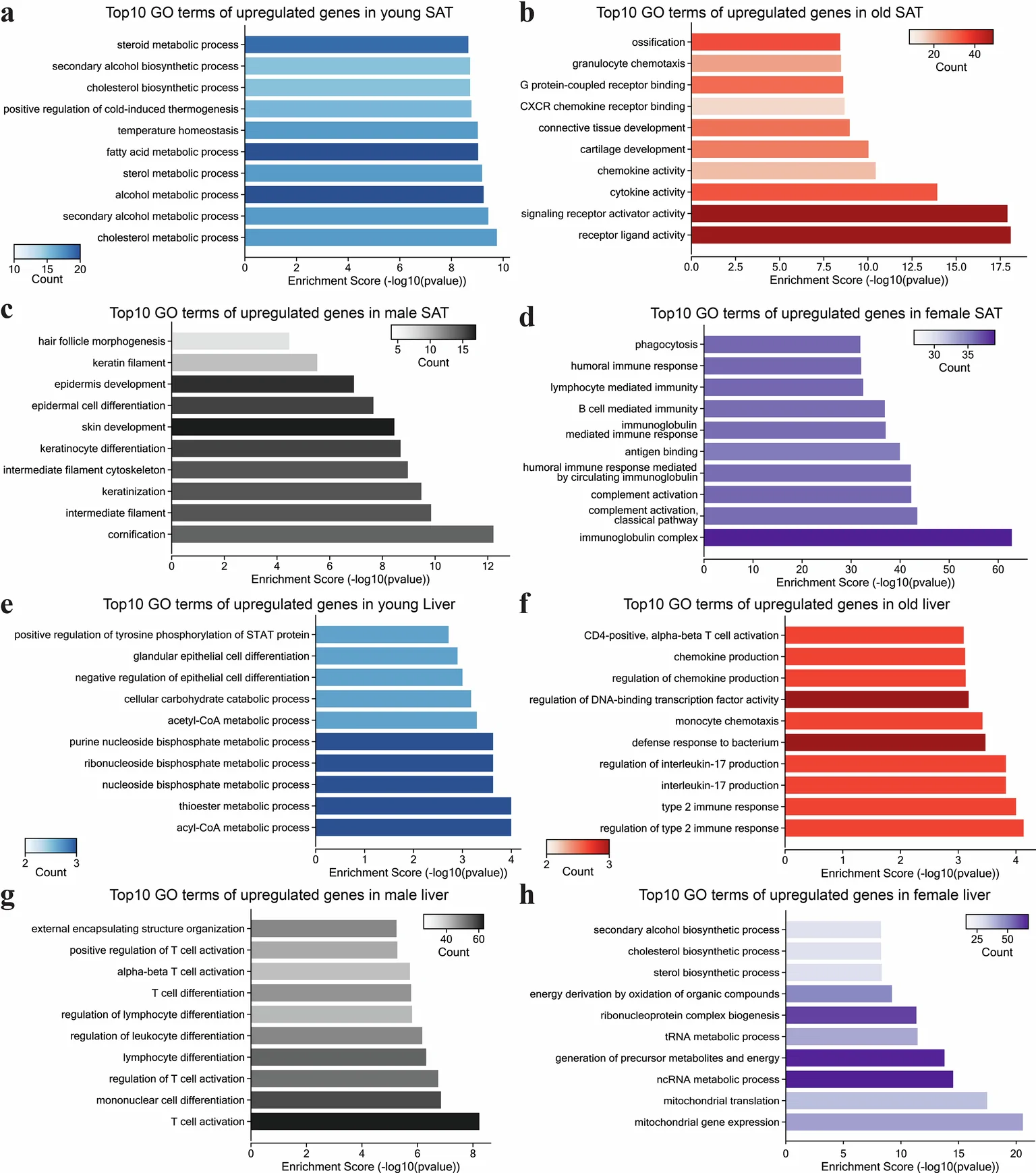
SAT and liver tissue transcriptomics analyses of age and sex variables using public database GTEx. (**a**-**d**) Top 10 GO terms enriched from upregulated genes in SAT young (20-29 year-old (a) vs old (60-69 year-old) (**b**) and male (**c**) vs female (**d**). (**e**-**h**) Top 10 GO terms enriched from upregulated genes in liver young (30-39 year-old) (**e**) vs old (50-69 year-old) (**f**) and male (**g**) vs female (**h**). Young vs old comparison of two tissues merged both male and female data. Male vs female comparison of SAT merged both young and old data, but of liver only used young data due to diminished sexual dimorphism in old liver. The age range in liver was off the proposed range because limited sample number and disease-biased 20-29-year-old cohort. 6 of top 10 GO terms enriched from female SAT dataset (**d**) and 9 of top 10 from smale liver dataset (**g**) related to immune responses, confirming the tissue-specific inflammation in the MPS set-up with the same set of serum (Extended Data Fig. 1a,e,f).

To determine whether on-chip aging faithfully recreates patterns of human aging, we compared our RNA-seq data with the human subcutaneous adipose tissue (SAT) database of GTEx (gtex_v8)^32^. We found that the top 10 overlapping Gene Ontology (GO) terms agreed between the human studies and the heterochronic WAT MPS and, importantly, suggested increased inflammation in OCM and improved metabolic processes in YCM (Fig. 3c and Extended Data Fig. 3a,b).

**Extended Data Fig. 4:**
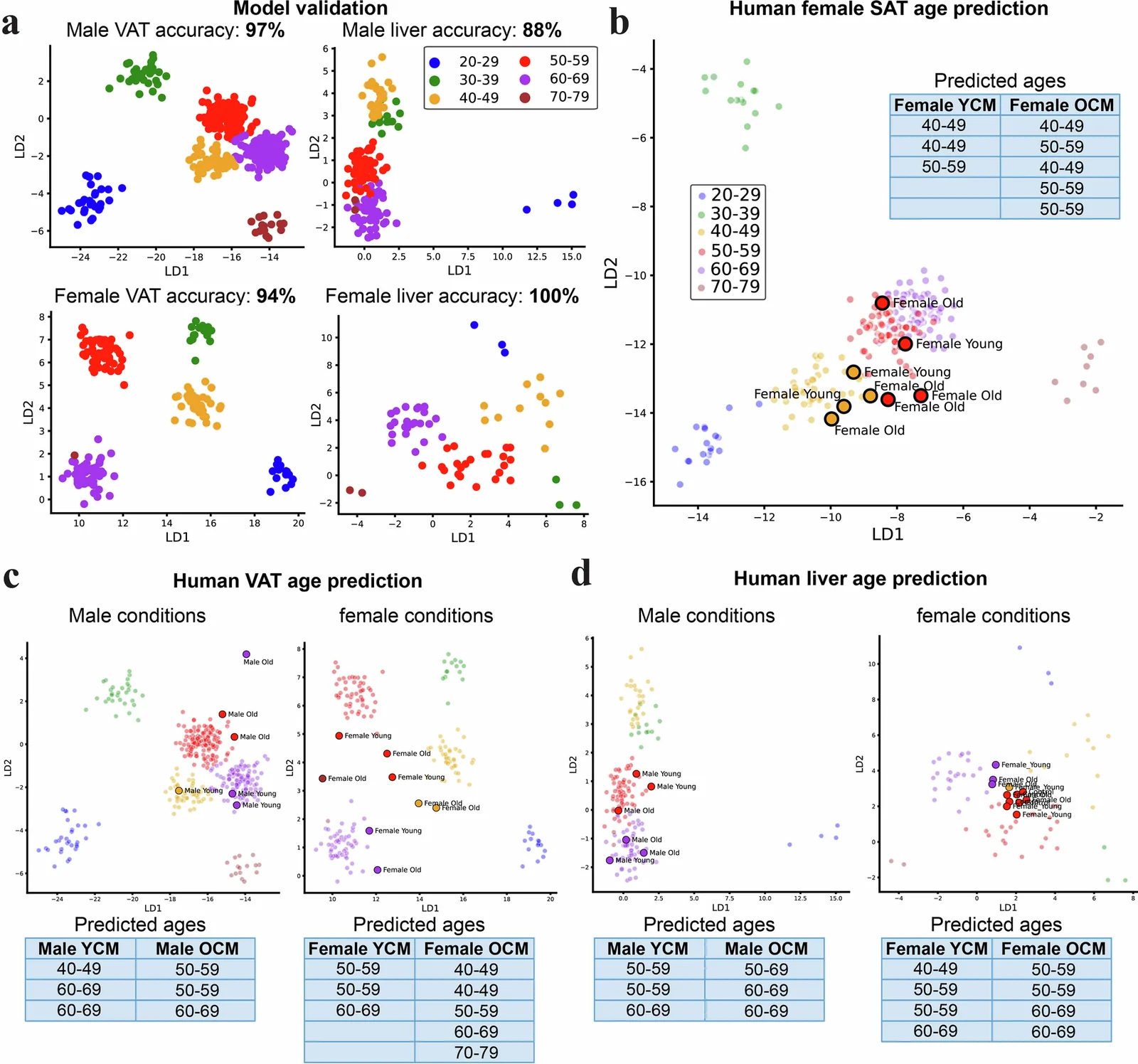
Additional human WAT and liver age prediction models. (**a**) model validation of male and female VAT and liver. (**b**) female SAT age prediction of WAT-MPS in female condition. (**c**,**d**) age prediction of both sex conditions of WAT-MPS in human VAT model (**c**) and liver-MPS in human liver model (**d**). Noting that only 65 female liver samples, 46 for machine-learning and 19 for validation, highly compromised the reliability of the model, even with a nominal 100% accuracy.

In an even more stringent test of the biomedical relevance of the on-chip aging, we compared the absolute age of fat MPS in YCM vs OCM with the chronological age of human fat donors. To this end, we developed a transcriptome-based age-predictor machine learning (ML) model for human WAT. This approach avoids biases in transcript feature selection, minimizing arbitrary errors and is comprehensive: it includes hundreds to thousands of transcript features per input, which is more than sufficient for the number of studied aging biomarkers. This machine learning pipeline was designed to be universally applicable to the studies of WAT aging. The models were trained on 70% of randomly selected samples from the GTEx database, with separate training based on sex and fat depots; in testing on the remaining 30%, the models demonstrated 90% accuracy for male SAT and 92% for female SAT; 97% for male visceral adipose tissue (VAT) and 94% for female VAT (Fig. 3d and Extended Data Fig. 4). Next, we used trained models on the SAT human database and tested them for predicting the biological age of the WAT MPS that were perfused with YCM vs OCM. We found a remarkably quick equilibration of hiPSC-derived tissue age towards the age of the serum donors (Fig. 3e).

In summary, the global gene expression that is rapidly established by heterochronic serum on-chip reflects the patterns of aging in human populations, and the age of human MPS is influenced by the age of serum donors.

### Biomarkers and mechanisms of plasticity in human WAT aging

To confirm and extrapolate the relevance of our findings to the fundamental process of human aging, we performed more comprehensive comparisons between the RNA-seq on the heterochronic MPS and the public databases CellGen, GenAge and Aging Atlas.

**Fig. 4:**
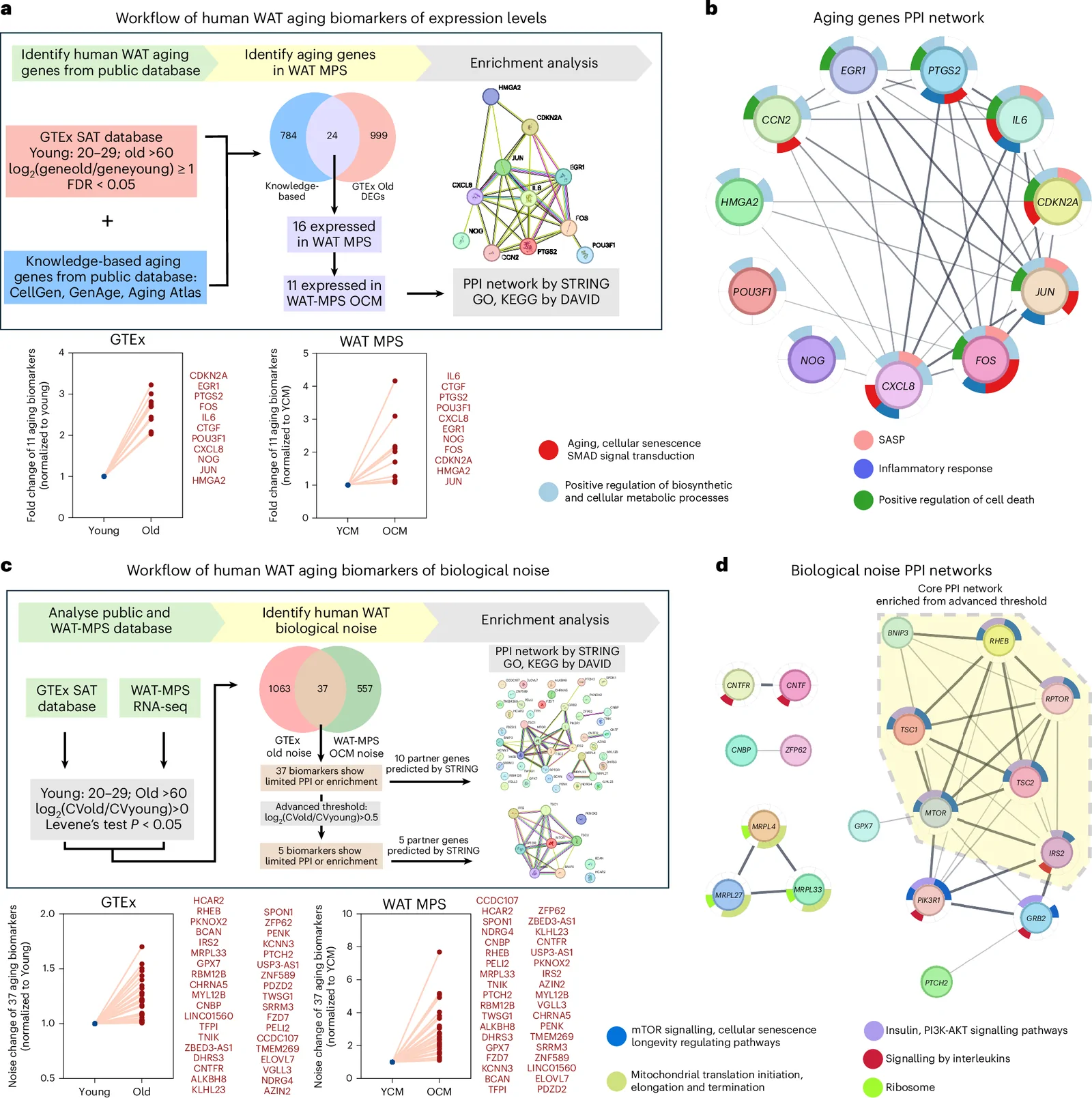
Serum-induced WAT-MPS aging recreates human in vivo aging, suggesting biomarkers and pathways. **a**,**c**, Workflow and visualization of aging biomarkers on expression levels (**a**) and biological noises (**c**). **b**,**d**, Protein–protein interaction networks enriched from the aging biomarkers of expression levels (**b**) and biological noises (**d**). **b** shows the network in degree-sorted layout. **d** only shows genes with interactions. STRING analysis predicted 10 associated partner genes from the original 37 genes of noise with confidence score >0.99. Highlighted core PPI network is associated with 5 noise genes and 5 STRING-predicted partner genes based on higher threshold, log_2_(CV_old_/CV_young_) > 0.5). *n* = 11 (**a**) and *n* = 37 (**c**). *n* refers to genes. Source data

**Extended Data Fig. 5:**
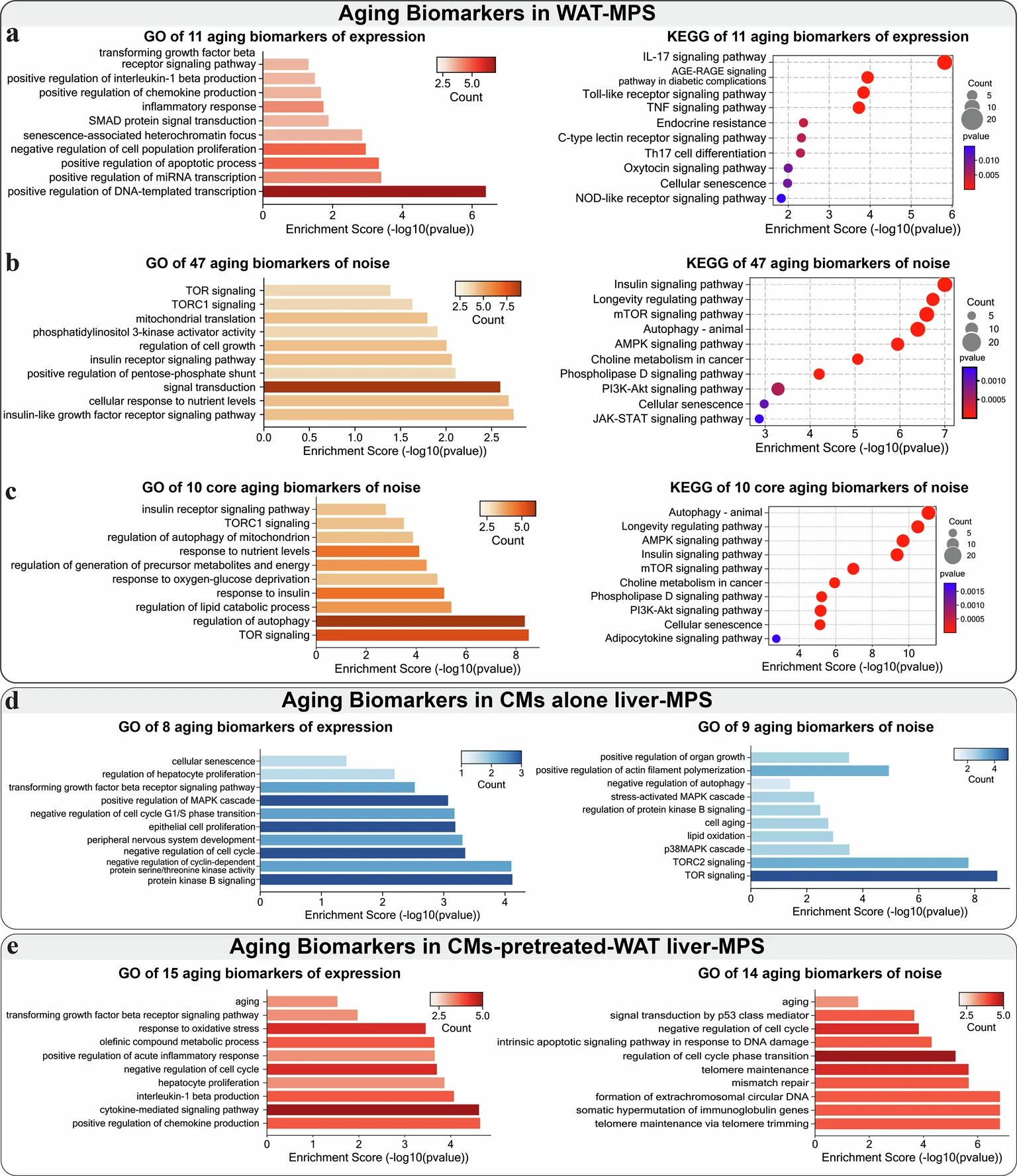
GO and KEGG enriched from aging markers. (**a**-**c**) GO and KEGG from the WAT biomarkers of transcriptomic *levels* (**a**) and *noises* with log2 (CV_old_/CV_young_) > 0 (**b**) and >0.5 (**c**). (**d**,**e**) GO from transcriptomic *levels* and *noises* in liver aging induced by CMs alone (**d**) and CMs-pretreated-WAT (**e**).

First, we focused on identifying the biomarkers of human aging that show the same differences on-chip (YCM vs OCM) and in vivo (young vs old humans), the conserved determinants behind systemically influenced plasticity of human WAT aging. We gated the union set of the Aging datasets by the GTEx SAT database (Fig. 4a), which yielded 24 overlapping genes, 11 of which also overlapped with our WAT-MPS RNA-seq, the 11 biomarkers of aging that exist in the human population and are established in 4 days on-chip by human serum. These 11 genes had an average 2.57-fold increase with aging in the GTEx and had on-average 1.91-fold increase in the OCM compared to YCM, in strong agreement with the human tissue aging in the MPS. Confirming the biomedical importance of our findings and expanding the understanding of aging, these 11 biomarkers include central hub genes *CXCL8* (*IL8*), *JUN*, *FOS*, *CDKN2A*, *IL6*, and the networks of aging-associated pathways, such as TNF, cellular senescence, NOD-like and TGFβ/SMAD (Fig. 4b and Extended Data Fig. 5a).

Not only did the global mRNA levels of on-chip aging correlate with the natural human aging but the same was also true for another hallmark of aging, transcriptional noise. Comparison between the GTEx and the MPS RNA-seq datasets revealed 37 biomarkers of transcriptional noise, which have increased standard deviation among older people and in the WAT MPS exposed to the OCM, as compared to YCM (Fig. 4c). These biomarkers of transcriptional noise, plus 10 STRING-predicted partner genes (all with combined confidence scores >0.99), underlay the systemically regulated plasticity of human tissue aging, interestingly providing critical links to mTOR and insulin signalling and outlining several networks that were less studied in aging (Fig. 4d and Extended Data Fig. 5b). With advanced threshold (log_2_(fold change) > 0.5), 5 most fluctuated genes together with 5 predicted partners identified the mTOR signalling pathway as the core network with aging-related increase in transcriptional noise.

### Biomarkers and mechanisms in human liver aging

Fat tissue ages early in life^2,3^ and is instrumental to the aging of other organ systems such as the liver, allowing us to compare two aging-inducing paradigms for the liver: direct serum exposure (YCM- vs OCM-treated liver, or CMs alone) and via intertissue communication between OCM-pretreated WAT interconnected to the liver MPS. We found that serum heterochronicity induced some common pathways in both fat and liver MPS including cytokine interactions (Extended Data Fig. 2b,e). Initial transcriptomic comparison of liver gene expression in serum heterochronicity vs heterochronic WAT–liver chip revealed both shared and unique features of each paradigm (Extended Data Fig. 2e,g).

**Fig. 5:**
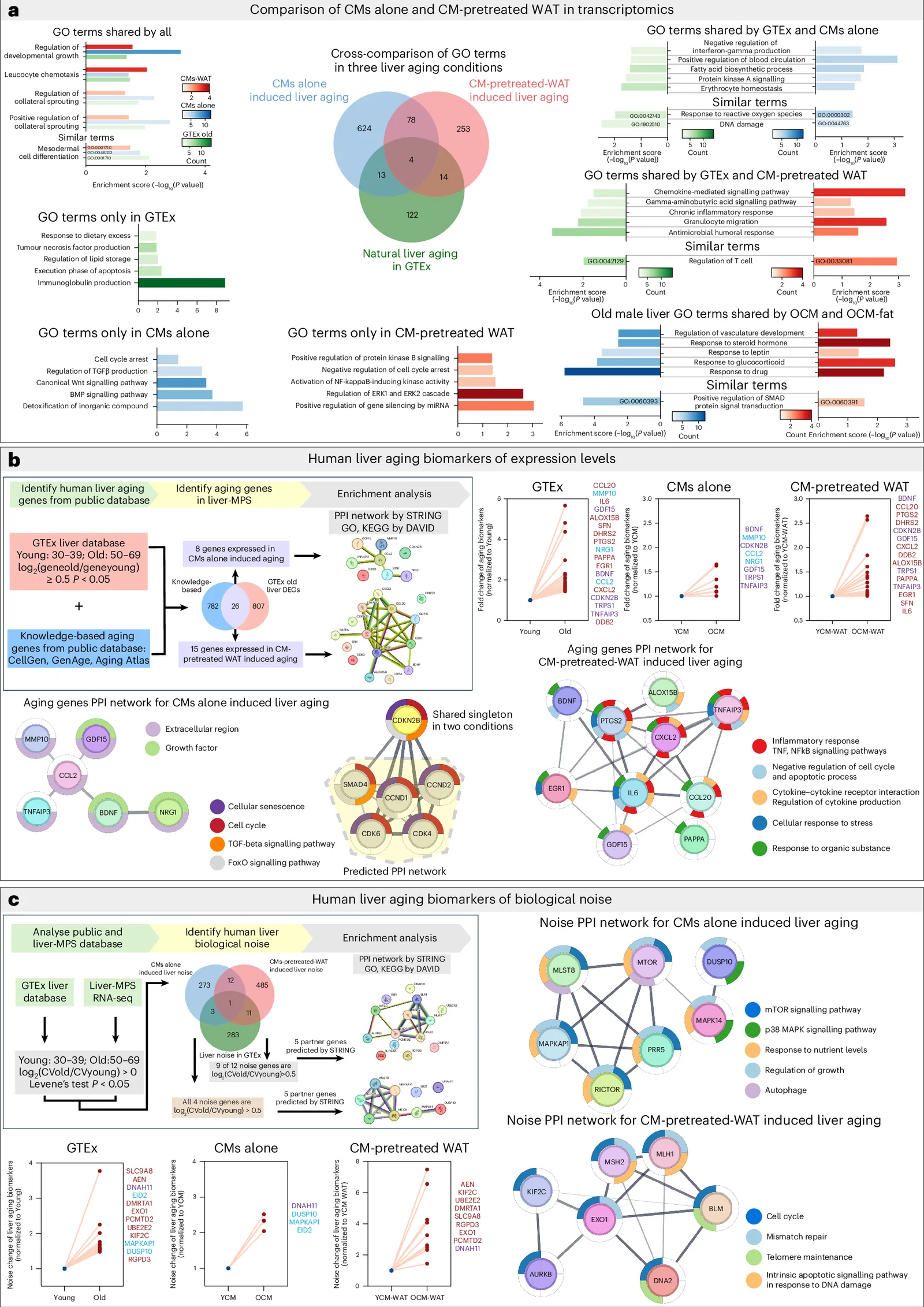
Transcriptomic analysis and identification of biomarkers and pathways in liver-MPS aging induced by CMs alone and CM-pretreated WAT. **a**, Cross-comparison of GO terms to GTEx male liver aging. **b**,**c**, Identification of aging biomarkers on expression levels (**b**) and biological noises (**c**) from GTEx liver aging of both sexes, aligning to Fig. 4. Both only show genes with protein–protein interactions, except for shared singleton CDKN2B. STRING analysis predicted 5 associated partner genes for noise genes with confidence score >0.99 in two aging conditions, and for CDKN2B with confidence score >0.95. Blue hallmark genes are shared by GTEx and CMs alone, red by GTEx and CM-pretreated WAT, and purple by all three. Source data

In a rigorous validation of biomedical importance, aging-associated genes from these datasets were further compared with the in vivo human liver aging signatures from the GTEx database (Fig. 5a). All three aging contexts showed some overlapping GO terms (for example, tissue development and immune responses), but the two liver-MPS models exhibited distinct aging signatures. Liver aging induced by OCM shared metabolic and oxidative DNA damage signatures with the GTEx human in vivo aging data, and also featured TGF superfamily signalling. In contrast, OCM-treated WAT induced liver aging with a more pronounced inflammaging signature than the direct OCM treatment; importantly, the inflammatory terms were also reflected in the in vivo GTEx liver data. A biological age prediction model for human liver was limited in its performance, probably due to the small training sample size (161 males and 65 females in GTEx), and only slightly differentiated the predicted ages between male YCM- and OCM-treated liver MPSs (Extended Data Fig. 4d).

On the basis of a similar workflow as in WAT MPS, we identified and compared aging biomarkers in the liver MPS under two conditions. At the expression level (Fig. 5b), 8 aging-associated genes induced by OCMs alone were primarily growth factors (*BDNF*, *GDF15*, *NRG1*), whereas 15 genes induced by OCM-pretreated WAT included not only growth factors (*BDNF*, *GDF15*) but also inflammatory mediators, most prominently *IL-6*. Both conditions shared *CDKN2B*, which, although isolated from the two primary networks, could independently form a PPI network with 5 predicted partners (confidence score >0.95), enriched in TGFβ-associated senescence pathways. Focusing on biological noise (Fig. 5c), the OCMs-alone condition showed strong association with mTOR signalling, consistent with the core PPI network identified in WAT MPS and highlighting mTOR dysregulation as a central feature of heterochronic CMs. In contrast, the heterochronic CM-pretreated WAT showed dysregulated maintenance and repair of chromosomal components without involvement of mTOR, a reasonable outcome given that the heterochronic serum factors were no longer present in the culture environment.

In summary, we describe critical biomarkers and mechanisms behind the systemically influenced plasticity of human tissue aging, both at the levels of gene expression and transcriptional noise.

### Testing rejuvenation strategies for vital human tissues

**Fig. 6:**
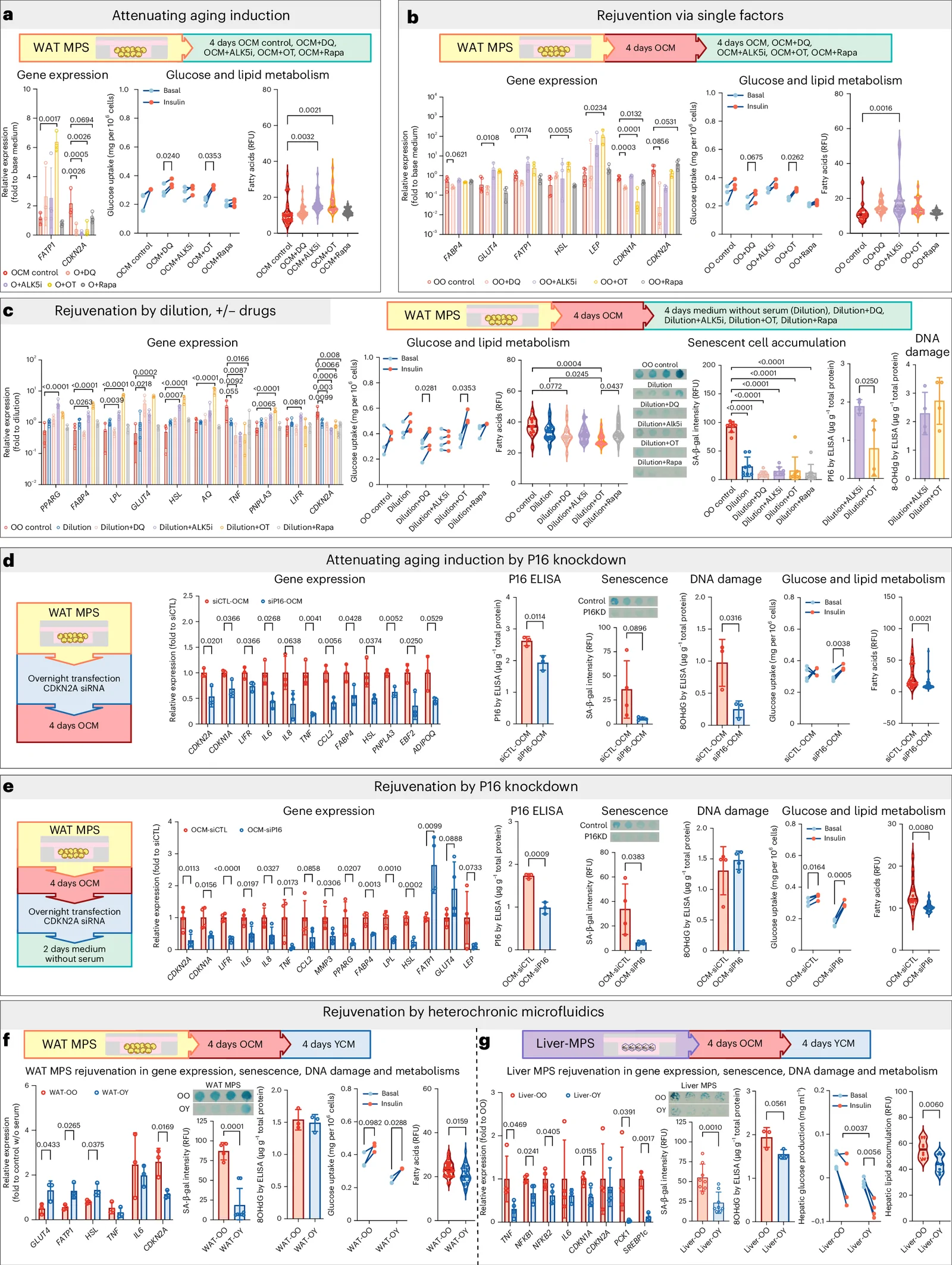
Rejuvenation approaches in the aging-on-chip MPS. **a**–**c**, Drug screening in WAT MPS by perfusing with OCM plus drugs for 4 days (**a**, *n* = 4 for ALK5i and Rapa, otherwise *n* = 3 in gene expression; *n* = 4 for Rapa, otherwise *n* = 3 for both basal and insulin conditions in glucose metabolism; *n* = 32 for ALK5i, otherwise *n* = 24 in lipid metabolism); OCM for 4 days, then OCM plus drugs for an additional 4 days (**b**, *n* = 5 for all of OO control and *n* = 4 for all of Rapa, otherwise *n* = 3 in gene expression; *n* = 4 for Rapa, otherwise *n* = 3 for both basal and insulin conditions in glucose metabolism; *n* = 16 for OO control and *n* = 13 for Rapa, otherwise *n* = 24 in lipid metabolism); or OCM for 4 days, then medium without old serum and plus drugs (**c**, *n* = 3 for all of OO control, OT and Rapa, otherwise *n* = 4 in gene expression and for both basal and insulin conditions in glucose metabolism; *n* = 13, 24, 16, 24, 22, 20 sequentially in conditions of lipid metabolism; *n* = 8 for all in SA-β-gal, *n* = 4 for all in P16 ELISA and DNA damage). **d**,**e**, Knockdown P16 by siRNA together with 4 days OCM (**d**, *n* = 3 for all in gene expression and DNA damage, *n* = 4 for all in SA-β-gal and glucose metabolism, *n* = 32 for all in lipid metabolism) or after OCM for 2 days (**e**, *n* = 3 for P16 ELISA, *n* = 14 for lipid metabolism, *n* = 4 for others). **f**,**g**, Heterochronic microfluidics by perfusion with OCM for 4 days, then YCM for 4 days in WAT MPS (**f**, *n* = 3 for all in gene expression, DNA damage and lipid metabolism; *n* = 4 for OO and *n* = 8 for OY in SA-β-gal, *n* = 30 for OO and *n* = 28 for OY for lipid metabolism) and in liver MPS (**g**, *n* = 3 for OY condition in *IL6* and both in *SREBP1c*, otherwise *n* = 4 for all in gene expression, *n* = 4 for all in glucose metabolism, *n* = 3 for all in DNA damage, *n* = 8 for all in SA-β-gal and lipid metabolism). *n* refers to one chamber in MPS in quantifications of SA-β-gal staining and lipid metabolism; *n* refers to media in glucose metabolism; otherwise, *n* refers to MPS. *P* values were calculated using paired two-tailed *t*-test for the comparison of basal vs insulin in all glucose metabolism; using two-way ANOVA for OO vs OY conditions of glucose metabolism (**g**); using one-way ANOVA followed by Dunnett’s test in expression comparison of each gene and lipid metabolism (**a**,**b**); using one-way ANOVA followed by Tukey’s test in expression comparison of each gene, lipid metabolism and SA-β-gal (**c**); using unpaired two-tailed *t*-tests for the other comparisons. Exact *P* < 0.1 are directly noted on the compared pairs. Data are presented as mean ± s.d. for all bar graphs. All genes were normalized to the dilution condition (**c**), otherwise to the first condition. Statistical methods and exact *P* values are shown in Supplementary Data 1. Source data are provided. Only significant gene pairs are shown, and all tested genes are shown in Supplementary Fig. 1. Source data

MPS are well known for their ability to support drug screens but had not been applied to testing rejuvenation strategies. Here we used the human on-chip aging for drug screens in two key directions: attenuation of aging and reversal of aging. In the first approach, WAT MPS was aged by OCM for 4 days, in the presence vs absence of a senolytics drug combination of dasatinib (200 nM) plus quercetin (20 μM) called DQ, or an ALK5 inhibitor of aging-elevated TGFβ signalling (ALK5i, 342 nM), or the aging-diminished hormone oxytocin (OT, 15 μM), or mTOR inhibitor rapamycin (Rapa, 20 nM) (Fig. 6a). In the second approach on pharmacological reversal of aging, after 4 days in OCM alone, that is, the aging, WAT MPSs were continued in the OCM for 4 more days in the presence or absence of DQ, Alk5i, OT or Rapa (Fig. 6b). As a variant of the approach on reversal of aging, we also examined whether dilution of old serum would synergize with the DQ, Alk5i, OT or Rapa (that is, after the 4 days in OCM, MPSs were washed with the base medium (5% knockout serum replacement, 0% old serum) and then kept for an additional 4 days, in the presence vs absence of DQ, Alk5i, OT or Rapa) (Fig. 6c).

All four drugs at all regimens as well as dilution of old serum attenuated expression of the senescence marker *CDKN2A* in the WAT MPS (Fig. 6a–c). However, not all parameters of aging were attenuated in the continuous presence of OCM by the tested drugs, with ALK5i and OT showing weak effects on adipogenesis (Fig. 6a,b). The most robust rejuvenation that included diminished SASP and improved metabolic parameters was observed when old serum dilution was combined with the tested longevity drug candidates (Fig. 6c). Moreover, in this setting, DQ and oxytocin robustly increased expression of the insulin-regulated glucose transporter, *GLUT4*, leading to functional recovery of insulin sensitivity in the glucose uptake assay. Oxytocin was the most effective in overriding old serum, decreasing senescence, reducing inflammation, promoting the highest expression of multiple important adipogenesis and function markers (*FABP4*, *LPL*, *HSL*, *ADIPOQ* and *PNPLA3*), and improving both glucose and lipid metabolism (Fig. 6c).

To test the functional relevance of regulatory nodes identified by transcriptomic analysis, we established in-device small interfering RNA (siRNA)-based knockdown strategies for the adipocyte MPS. On the basis of our aging hallmark analyses, a key biomarker of senescence, *CDKN2A* (p16), emerged as a central regulatory node, showing strong associations with aging and functionally linking the other 10 markers through upstream and downstream signalling pathways. For example, knockdown of *CDKN2A* suppresses senescence-associated cell cycle arrest, attenuates the autocrine pro-aging effects of SASPs (such as *IL-6* and *IL-8* (ref. ^33^), and enhances long-lasting metabolic health in mice^34^. To test whether these p16-related rejuvenation features would manifest in our human aging-on-chip, we performed siRNA-mediated knockdown of *CDKN2A* both concurrently with and following OCM treatment (Fig. 6d,e). In both scenarios, *CDKN2A* knockdown markedly reduced aging phenotypes and enhanced metabolic function, with greater efficacy observed when knockdown was applied after OCM removal (that is, in combination with serum dilution).

Inspired by the systems of heterochronic parabiosis^35^ and heterochronic blood exchange^11,12^, we next examined whether switching from OCM to YCM can reverse the experimental on-chip aging for WAT MPS and liver MPS. The MPSs were sequentially perfused with medium containing 5% serum from old donors for 4 days and were then switched to medium containing 5% serum from young donors for another 4 days (OY). Control isochronic MPSs were treated for 8 days with medium containing 5% serum from old donors (OO).

As compared with OO, the OY serum switch in WAT MPS resulted in lower levels of *CDKN2A* and higher levels of the metabolism markers: *GLUT4*, *FATP1* and *HSL*, accompanied by recovery of insulin sensitivity in glucose uptake assay and reduced fatty acid uptake (Fig. 6f). Based on the bulk RNA-seq of WAT MPS, the OY switch promoted transcriptional rejuvenation of immune responses, metabolism, cell fates, tissue remodelling, rejuvenated *CEBPB*, *CEBPD* and *SLC2A4* (*GLUT4)* network, and normalized TGFβ/Smad signalling, which suggests improvements in adipogenesis, insulin sensitivity, glucose metabolism and signal transduction (Extended Data Fig. 7b). The exposure of WAT MPS to OCM after YCM pretreatment (YO) induced pro-geronic features, as compared to (YY), including DNA damage responses via p53 (Extended Data Fig. 7c), and patterns of higher adiposity and insulin resistance (Extended Data Fig. 7g).

For the liver MPS, the switch from old to young serum (OY) reduced several inflammation markers (*TNF*, *NFKB1*, *NFKB2*) and decreased senescence, based on the levels of *CDKN1A*, all as compared to OO (Fig. 6g). A key marker of glucose metabolism, *PCK1*, and a determinant of lipid metabolism, *SREBP1c*, were attenuated, accompanied by healthier physiologic responses: less glucose production, better insulin sensitivity and lower lipid accumulation.

Overall, the above results demonstrate not only that human tissue aging is rapidly induced on-chip, but also that human tissue rejuvenation is equally rapid, and moreover, only achievable by some approaches, enabling selective screens for longevity therapeutics.

## Discussion

This study provides deep insights on the paradigm of plasticity in aging, its inducibility and attenuation, and applies these concepts to cell types from vital human tissues ex vivo. While we acknowledge that WAT and liver consist of more cell types than adipocytes and hepatocytes, respectively, and thus that the WAT and liver MPS are a simplification of complex tissues, OCM-driven on-chip aging of these human iPSC-derived cells accurately reflects the physiological in vivo process, and is in agreement with clinical observations while markedly improving our understanding beyond animal models and cell cultures^11,12,19,20^. It informs on key age-related influences of the human systemic milieu on health, function and crosstalks of human tissues. The discovered attributes of human aging, for example, signalling networks, knock-on effects of fat aging on liver, sexual polymorphism and tissue memory of age would have been hard or impossible to uncover using previous approaches^36^, including previously published reports with animal alternative aging models (Supplementary Table 1).

Although sex chromosomes are considered the primary drivers of sexual dimorphism, this study demonstrated that sexually dimorphic responses can also be elicited by the biological sex of the serum donor, even when using a single XY hiPSC line. To our knowledge, such a strategy for inducing sex-biased gene expression in iPSC-derived adipocytes and hepatocytes has not been previously reported. Moreover, serum-induced transcriptomic signatures of sexual dimorphisms in MPSs were, intriguingly, found to be tissue specific and, when benchmarked against published human databases^37–39^ (Extended Data Fig. 1d–g), confirmed that sex-specific patterns found in human WAT and liver are faithfully recapitulated on-chip. Specifically, inflammatory and immunoregulatory pathways were higher, while metabolic processes were lower in female serum WAT MPS^37,38^, but such pattern was observed in male serum liver MPS^39^, suggesting that on-chip sexual dimorphisms are driven by complex serum-derived factors with tissue-specific effects that go beyond inflammatory cytokines.

Notably, the extended list of SASPs also revealed sex-biased aging phenotypes (Extended Data Fig. 8a). Under OCM versus YCM conditions, male MPSs exhibited stronger differential expression of inflammatory markers, indicating a more pronounced aging response than in females, particularly in WAT MPS. This aligns with the well-documented observation that males generally age faster and have shorter lifespans than females^40^. However, at the same YCM condition, female WAT MPS demonstrated more pronounced proinflammatory responses compared with male WAT MPS (Extended Data Fig. 8b,c).

Another sex-specific influence was a decreased resolution of the phenotypes that were induced by OCM vs YCM in the female serum-treated MPS. This included a broader distribution in the PCA (Extended Data Fig. 1b), higher transcriptional noise (Extended Data Fig. 1c) and lower accuracy or reliability of age predictions for MPSs (Extended Data Fig. 4). Such on-chip sex differences agree with the clinical observations that female aging is more complicated, varied and less predictable perhaps due to hormonal fluctuations, pregnancy and menopause^41–43^. In addition, the models we used for female aging phenotypes are expected to be less powerful in predictive power due to fewer available samples for machine learning (445 male and 218 female SAT samples; 371 male and 170 female VAT samples; 161 male and 65 female liver samples in GTEx). The limited sample size highlights that subcutaneous WAT is a far more practical tissue for model training and aging prediction than liver, as liver sampling via intraperitoneal procedures is considerably more invasive and carries greater risks compared with the relative ease of subcutaneous adipose sampling.

The comprehensive comparison of the MPS RNA-seq with human transcriptomics, including through our ML age predictor, demonstrates that the on-chip-controlled aging closely recreates in vivo human aging. This transforms the paradigm of biological aging, reducing its reliance on progression of time and highlighting the pivotal role of the circulatory environment in time-independent induction and reduction of human tissue aging.

As a technical limitation, we should note that our results are based on bulk RNA-seq data rather than single-cell RNA-seq approaches^44^ and thus studied noise in gene expression using population noise rather than cell-to-cell noise. However, given the limited availability of larger cohorts of single-cell RNA-seq data for mature adipocytes, our analysis strategy was the currently best option.

Beyond transcriptome-based age prediction models, aging clock algorithms utilizing proteomics, metabolomics and imaging modalities are currently under development^45^. Our system enables rapid monitoring of metabolite and cytokine dynamics through analysis of BONCAT de novo proteomics on microfluidics, effluent medium and microscopy, facilitating continuous tracking of the aging process without disrupting MPS cultures. The integration of multiple indicators, including fatty acids, glucose and SASP factors in the effluent medium, along with cellular morphology and intracellular lipid droplet dimensions obtained via imaging, provides orthogonal validation and enhances the precision of aging status assessment in live MPS.

Our system showed that inflammatory cytokines and growth factors, the two major components of SASPs^46,47^, were primary drivers of aging, but their roles differed between WAT and liver: OCM-induced liver aging showed a stronger dependence on growth factors (*BDNF*, *GDF15*, *NRG*) rather than on inflammatory cytokine (*CCL2*). This was accompanied by activation of the growth-factor-responsive cell cycle regulator *CDKN2B* (p15) via the TGFβ/SMAD pathway, and induction of an inflammation suppressor, *TNFAIP3* (A20), to inhibit the NF-κB pathway. In contrast, WAT aging showed a clear inflammatory propensity, characterized by an aging loop involving immediate–early transcription factors (*JUN*, *FOS*, *EGR1*), the senescence centre *CDKN2A* (p16), and key inflammatory triggers, modulators and SASPs (*PTGS2*, *IL-6*, *IL-8*) that reinforce autocrine senescence and propagate paracrine aging signals.

TGFβ, a growth factor and SASP component that is elevated during aging^47^, may serve as a central regulator distinguishing WAT from liver aging, as it suppresses adipogenesis^48^ while exhibiting complex effects on hepatocytes in coordination with other growth factors^49–52^. This role is strongly supported by our findings: in WAT MPS, YCM and OCM induced divergent genotypic and phenotypic outcomes, whereas in liver MPS, both conditions consistently converged towards enhanced developmental and hepatogenic programs.

Interestingly, OCM-pretreated WAT caused liver aging with similar features to that of OCM-induced aging and both processes closely resembled in vivo aging (overlapped GO terms and aging biomarkers of expression and biological noise). Growth factors (*BDNF*, *GDF15*) and aging-related regulatory pathways (*CDKN2B*) as well as the inflammation suppressor *TNFAIP3* were upregulated in the liver exposed to OCM-aged WAT. However, inflammaging signatures (*EGR1*, *PTGS2*, *IL6*) and proinflammatory cytokines/SASPs (*CCL20*, *CXCL2*) were also elevated. Based on the secretome analyses of OCM- vs YCM-treated WAT (Fig. 2g,h and Extended Data Fig. 8a) and TNF ELISA (Fig. 1b), the heterochronic interorgan signatures were due to SASPs secreted from the OCM-aged WAT and hepatogenic growth factors secreted from the YCM-exposed youthful WAT (*BMP4* (ref. ^53^), *FGFs*^54^).

**Extended Data Fig. 6:**
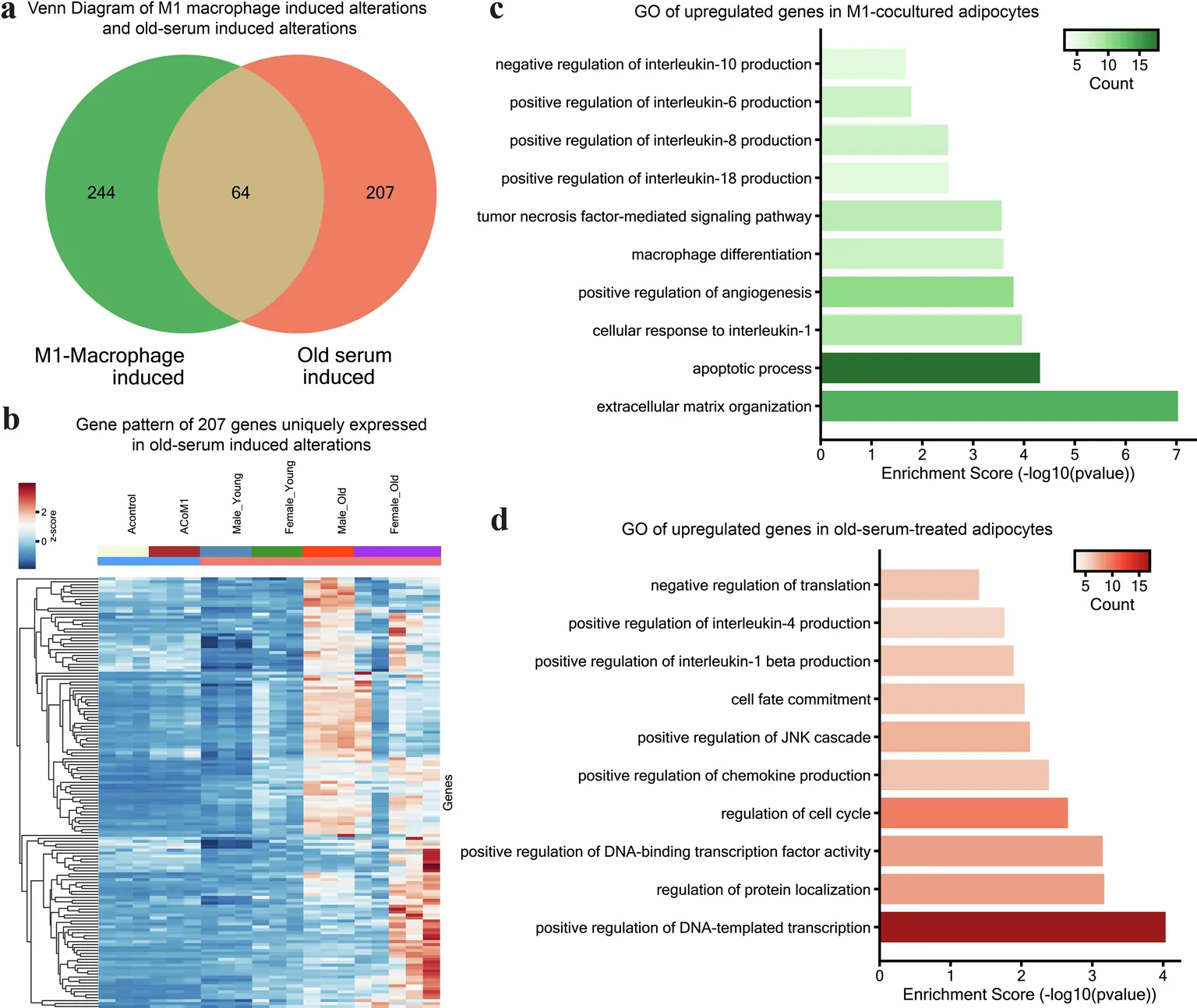
Transcriptomic comparison of old serum-induced and pro-inflammatory M1 macrophage induced alterations in WAT. (**a**) Venn plot of DEGs upregulated in old serum treated iADIPO-MPS and DEGs upregulated in M1 macrophage co-cultured iADIPO. (**b**) Heatmaps of genes uniquely induced by old serum. (**c**,**d**) Selected GO terms enriched from upregulated genes in M1-macrophage-induced (**c**) and old-serum-induced conditions (**d**).

WAT inflammation is tightly linked with aging, but can also be induced by other pathological events, such as obesity resulting in the recruitment of proinflammatory (M1) macrophages^22^. Although obesity and aging share many similar hallmarks and feed forward each other, termed by some as obesageing or adipaging^6,55^, the understanding of their differences is still limited. Here we were able to expand on this important topic by comparing on-chip aging to on-chip inflammation driven by isogenic iPSC-derived M1-like activated macrophages, as in our previous study^22^ (Extended Data Fig. 6). Both conditions manifested inflammatory features; however, WAT remodelling with a strong interleukin production occurred after the M1 infiltration, whereas OCM-treated WAT had severe alterations in transcriptional and translational processes that are common in aging and are observed in heterochronic parabiosis^31^. This approach highlights the capacity of our system to distinguish the clinically relevant details of inflammation, aging and obesity^2^.

While not exhaustive, we tested multiple anti-geronic strategies to demonstrate the value of our aging-on-a-chip system for longevity drug discovery applications. Serum dilution, although a simple approach, proved highly effective in removing aging-increased systemic factors, thereby attenuating aging phenotypes on-chip and enhancing the efficacy of additional interventions. This finding aligns with the growing clinical interest in therapeutic plasma exchange (TPE) as an emerging rejuvenation modality^56^. Oxytocin that had efficacy in reversing on-chip aging, demonstrated anti-geronic effects in vivo, in mice: simultaneously promoting lipolysis, reducing inflammation and correcting visceral fat redistribution^57–61^. Inhibition of TGFβ signalling through ALK5 also produced robust rejuvenation effects on-chip, in agreement with in vivo alleviated TGFβ/SMAD3-driven senescence, and simultaneous rejuvenation of muscle, liver and brain in mice^62,63^. Furthermore, in an excellent evolutionary and in vivo to MPS conservation, treatment of already old and frail mice with oxytocin plus Alk5i extended their lifespan and healthspan by over 72%, intriguingly, only in males^64^, and allowed both male and female old mice to exercise without heart fibrosis and inflammation^65^.

**Extended Data Fig. 7:**
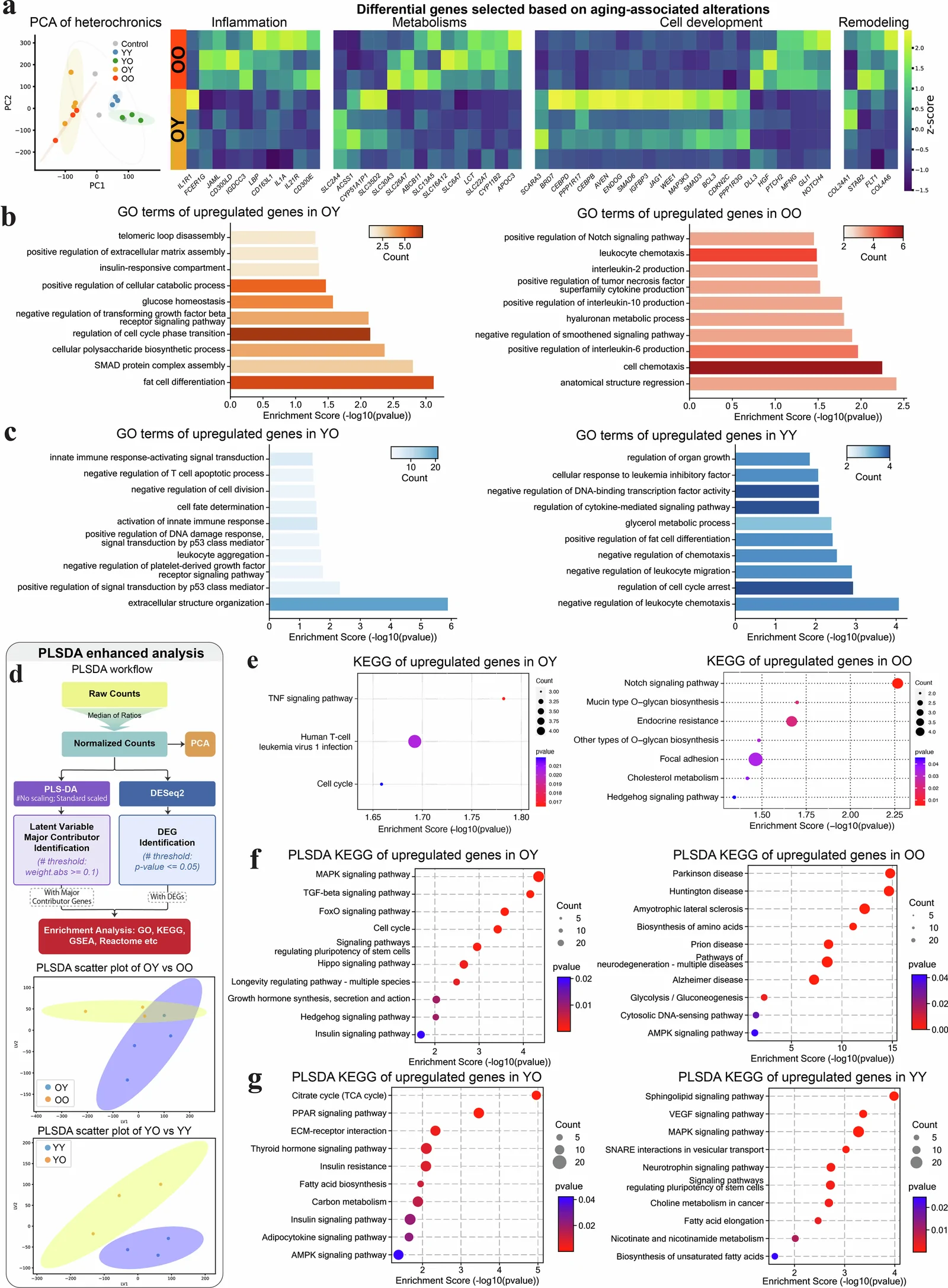
Additional transcriptomic analysis of heterochronic serum exchange in WAT-MPS. (**a**) Principal components analysis regarding on all heterochronic conditions and differential gene list of heterochronic microfluidics in OO vs OY. (**b**) GO terms enriched from upregulated genes in OY and OO. (**c**) GO terms enriched from upregulated genes in YO and YY. (**d**) Workflow and results of supervised PLSDA scatter plots of heterochronic conditions for enhanced comparison. (**e**,**f**) KEGG pathways of upregulated genes in YO and OO before (**e**) and after (**f**) PLSDA enhancement. (**g**) PLSDA-enhanced KEGG pathways of upregulated genes in YO (**f**) and YY (**g**).

**Extended Data Fig. 8:**
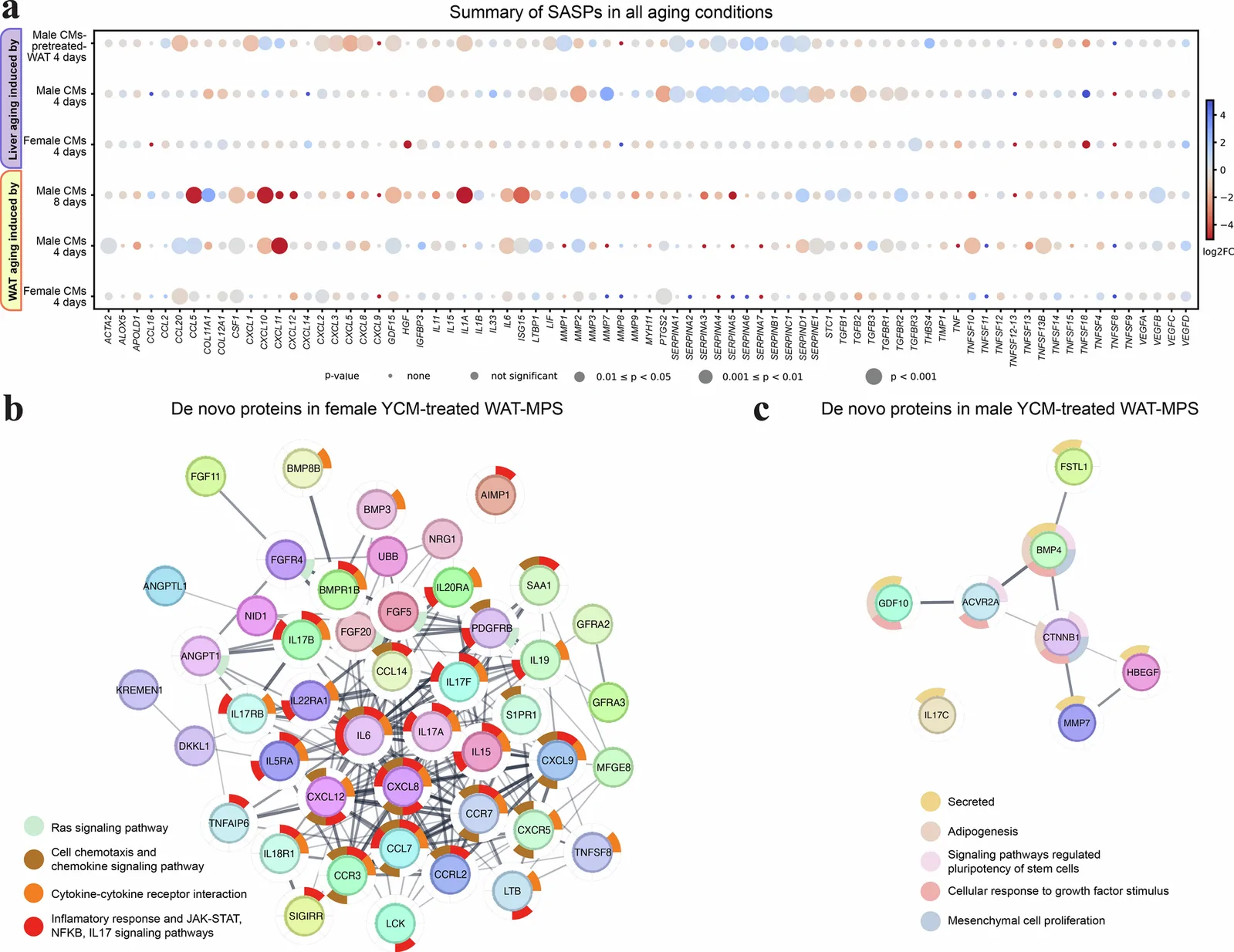
Secretomic analysis based on SASP genes and de novo proteogenesis. (**a**) Summary of major SASP expression in all measured aging conditions. P as ‘none’ because the raw counts with numerous zero-expression samples could not be processed through the transcriptomic analysis pipeline. While there are still two meaningful cases: expression detected only in young samples (extreme blue) or only in old samples (extreme red). (**b**,**c**) De novo proteogenesis in WAT-MPS derived from MetRS^L274G^ hiPSCs for comparisons of female (**b**) and male (**c**) YCM conditions. Singleton genes without enriched functions were hidden.

Our WAT MPS exhibited a clear tissue memory of aging, including complex processes ranging from epigenetic regulation to persistent senescence. Although certain phenotypes were partially alleviated, aging signatures persisted in OCM-treated WAT for at least 4 days after serum withdrawal, as shown in the OO control versus dilution condition (Fig. 6c). This retained aging memory was propagated to interconnected liver MPSs through the circulating medium in a para-/endocrine-like manner (Fig. 2), resulting in phenotypes uniquely observed in OCM-treated WAT MPS (for example, aging markers *IL6*, *PTGS2*, *EGR1*) but absent in OCM-treated liver MPS (Fig. 5b and Extended Data Fig. 8a). Such persistence of memory may also explain the blunted rejuvenating effect of YCM in heterochronic microfluidic conditions (OY vs OO in Fig. 6f, where *TNF* and *IL6* are unchanged; Extended Data Fig. 7f, where TNF signalling remained upregulated), as well as the reduced pro-aging impact of OCM (YO vs YY in Extended Data Fig. 7g). These phenomena bear similarities to the obesogenic memory observed in human WAT that leaves the tissue prone to long-term inflammation and insulin resistance even after obesity is reduced^66–68^. Similarly, a history of obesity, particularly in infancy and childhood, makes it easier to regain and harder to lose weight^69,70^.

One limitation of inducing aging through blood-borne factors is the variability of serum composition among individual donors. In our preliminary studies, we observed that this variability was particularly pronounced in samples from regions with a high prevalence of obesity. To minimize such bias, we selected serum samples from sub-Saharan populations, where the average obesity rate remains below 15%. Similarly, other confounding factors, such as medication use, dietary habits and lifestyle, may further influence the outcomes of aging induction. From a positive perspective, this variability provides an opportunity to investigate cohort-specific effects driven by geography, genetics and lifestyles (for example, caloric restriction^71^, exercise^72^, alcohol^73^) in future work with larger donor pools and platforms with high experimental scalability. Given the practical limitations of human serum availability, our miniaturized and cost-efficient system established the key parameters of controlled human tissue aging on-chip for such future explorations.

Overall, we hope that our promising aging-on-a-chip approach will empower research to further understanding of human aging, provide mechanistic insights into heterochronic serum exchange, as well as discover and test anti-geronic strategies in a preclinical model with high human relevance.

## Methods

### Ethics statement

This research complies with all applicable ethics regulations. The study protocols receive annual approval from the Committee on Laboratory and Environmental Biosafety (CLEB), the Stem Cell Research Oversight (SCRO) Office, and the Office of Environment, Health and Safety (EH&S) at the University of California, Berkeley.

All human serum samples used in this research were obtained from a commercial vendor (Metaphor Laboratory), ensuring compliance with US Department of Health and Human Services regulations. As these samples were fully de-identified by the vendor, no identifying information is accessible, and they do not qualify as ‘human subjects’ under research regulations. Essentially, the samples are anonymous and not linked to any living individual. Serum samples were from healthy donors of sub-Saharan African descent, including young (ages 21–34) and older donors (ages 62 and above). Supplementary Table 2 provides detailed information, including sex (male or female) and exact ages.

### MPS device fabrication

MPS devices were fabricated in accordance with our previous protocol^21^. Briefly, photolithography patterned SU-8 (3100, MicroChem) master templates were replica moulded to polydimethylsiloxane (PDMS, Sylgard 184, NC9285739, Fisher Scientific) by soft lithography. The inlet/outlets were holed using a 0.75-mm biopsy punch (504529, World Precision Instruments). The cell chambers were circular with diameter of 1,500 μm and thickness of 60 μm. A polyethylene terephthalate (PET) isoporous membrane (030060, TRAKETCH, SABEU) was activated by oxygen plasma (Plasma Equipment) at 60 W under ~0.6 Torr for 60 s and then chemically decorated in 2% bis(3-(trimethoxysilyl)propyl)anime solution (413356, Sigma-Aldrich) in 97% isopropyl alcohol (A451-4, Fisher Scientific) and 1% deionized water (Arium Mini, Sartorius) for 30 min at 80 °C. After decoration, the membrane was rinsed in pure isopropyl alcohol and sandwiched by oxygen plasma activated PDMS cell chambers and media channel slabs. The device was then baked at 110 °C for 30 min for solvent removal, bonding stabilization and device sterilization.

### iPSC cell culture and differentiation

All experiments used the healthy human male G15.AO iPSC line (RRID:CVCL_V192), except for the de novo proteomic analysis which used the healthy human male WTC11 iPSC line (GM25256 hPSCreg: UCSFi001-A) with stable expression of a mutant methionyl-tRNA synthetase (MetRS^L274G^, detailed protocol of cell line establishment in Supplementary Method 1). Both iPSCs were used at passage numbers below 80. When iPSCs reached 80% confluence, they were dissociated using ReLeSR (100-0483, StemCell Technologies) and subcultured at a 1:10 ratio in mTeSR Plus medium (100-0276, StemCell Technologies) on Matrigel-coated substrates (356231, Corning). Authentication was confirmed before experimentation by single nucleotide polymorphisms analysis. Mycoplasma testing was conducted before all experiments and annually. Sterility was checked daily. The undifferentiated state, characterized by colony morphology, was verified before each differentiation. Pluripotency was assessed before all experiments and regularly, on the basis of signature gene expression comparisons to endodermal and mesodermal differentiated states, and statistically analysed using *t*-tests.

Differentiation of adipocytes was described in our previous study^21^. Briefly, hiPSCs were first differentiated to mesenchymal progenitors (iPSC-MSCs) (STEMdiff Mesenchymal Progenitor kit, 05240, StemCell Technologies) and transduced for chemically inducible PPARγ. Adipose differentiation of iPSC-MSCs was induced in complete media (DMEM/F12, 11320033, Gibco) containing 1% HEPES (15630080, Gibco), 1% penicillin/streptomycin (15140122, Gibco) and 10% fetal bovine serum (EF-0500-A, Equafetal) with supplements of 0.25 μM dexamethasone (D1756, Sigma-Aldrich), 0.25 mM 3-isobutyl-1-methylxanthine (I5879, Sigma-Aldrich), 100 nM rosiglitazone (R2408, Sigma-Aldrich) and 500 nM insulin (0002-8315-01, Humulin R, Eli Lilly) for 4 days (days 0–3). Subsequent differentiation was completed in media supplemented with insulin and rosiglitazone at the same concentration (days 4–14). Exogeneous PPARγ was induced by 1 μg ml^−1^ doxycycline (D5207, Sigma-Aldrich) from day 0 until the end. On day 14, adipose expression was comparable to that of primary adipocytes collected from human biopsies^21^.

Hepatocytes were generated as previously described^74^. Differentiation was performed at 37 °C in 5% CO_2_ and 5% O_2_ unless stated otherwise. Endoderm was induced using endoderm-induction media (EIM), consisting of RPMI 1640 medium (11875093, Gibco) containing 2% B27 without insulin (A1895601, ThermoFisher), 1% Glutamax (35050061, Gibco), 1% non-essential amino acids solution (NEAA, 11140050, Gibco), 0.5 mM sodium butyrate (B5887, Sigma-Aldrich) and 100 ng ml^−1^ activin A (78001, StemCell Technologies) for 7 days. The following compounds were added to EIM during the first 3 days: 3 μM CHIR99201 (100-1042, StemCell Technologies) on day 1, 20 ng ml^−1^ basic fibroblast growth factor (FGFb, 100-18B, Peprotech) and 10 ng ml^−1^ bone morphogenetic protein 4 (BMP4, 120-05ET, Peprotech) on days 1 and 2, 50 nM PI103 (501932056, ThermoFisher) on days 1–3, and knockout serum replacement (KSR, 10828010, Gibco) at 2% on day 1, 1% on day 2 and 0.2% on day 3. On days 8–17, cells were cultured in hepatic induction media (HIM) consisting of IMDM (12440053, Gibco) containing 2% B27 without insulin, 1% Glutamax, 1% NEAA, 100 nM dexamethasone, 100 nM insulin and 0.5 mM 1-thioglycerol (M1753, Sigma-Aldrich). The following compounds were added to HIM: 10 ng ml^−1^ FGFb and 20 ng ml^−1^ BMP4 between days 8 and 17, 20 ng ml^−1^ hepatocyte growth factor (HGF, 100-39, Peprotech) between days 12 and 17. On days 18–22, cells were cultured in Hepatocyte Culture Media BulletKit (HCM, CC-3198, Lonza) without epidermal growth factor, including 20 ng ml^−1^ oncostatin M (300-10, Peprotech) and 20 ng ml^−1^ HGF. Media were changed daily during differentiation and maintenance.

### MPS culture

For WAT MPS, differentiating iPSC-MSCs on day 4 were dissociated (TrypLE Express, Gibco) and loaded into the cell chamber at a density of 8 × 10^7^ cells per ml. The MPS was connected to catheter couplers (SC20/15 and SP20/12, Instech Laboratories) and tubes (06422-00, Cole-Parmer). Culture media were perfused at a flow rate of 10 μl h^−1^ using a syringe pump (703007, Harvard Apparatus). For liver MPS, hepatocytes on day 22 were loaded similarly as WAT MPS and cultured in HCM.

### Old and young circulatory milieu treatment

The effectiveness of young and old circulatory environments was first assessed and validated in the WAT MPSs, which were perfused for 4 days using HPLM medium (A4899101, Gibco) containing 5% human serum respectively from 8 donors: 4 males (2 young and 2 old) and 4 females (2 young and 2 old), with *n* = 3 or 4 per donor. A time-course pilot study with OCM revealed that a 4-day exposure was sufficient to induce robust aging-associated alterations (Supplementary Method 2), which was therefore adopted as the standard condition in all subsequent experiments. We confirmed that the induction of aging was consistent across donors despite individual variations, and the response was more pronounced in male sera (Figs. 1a–e, 3 and 4). Although sexual dimorphism was less prominent in the liver MPS compared with the WAT MPS, it was still evident when comparing young male and young female CMs (Extended Data Fig. 1a). To focus on aging and to minimize donor and sex-related variability, HPLM media containing 5% pooled male sera pooled from 5 donors per age group were used as OCM and YCM for liver MPS, knock-on treatments and anti-aging interventions (Figs. 1f–i, 2 and 5).

### Interconnection of WAT and liver MPS

On the basis of our previously established design^23^, we interconnected CM-pretreated WAT MPS and liver MPS via a microlitre-scale tubing system that enabled a single-pass flow from WAT to liver. The cell ratio between adipocytes and hepatocytes was maintained at 5:1, as optimized in our previous study, where the liver MPS was not directly burdened while remaining sensitive to the lipidemic flux and inflammatory milieu from WAT MPS. Before connection, the WAT MPS was flushed with HPLM supplemented with 5% KSR under continuous flow at 30 μl min^−1^ for 1 min to remove residual serum-containing medium and eliminate potential air bubbles. Afterwards, the interconnected WAT–liver MPS system was perfused with HPLM supplemented with 5% KSR at a controlled rate of 20 μl h^−1^ from WAT to liver using a syringe pump.

### Drug administration

For drug testing, a senolytic drug combination of 200 nM dasatinib (SML2589) plus 20 μM quercetin (Q4951, both from Sigma-Aldrich), 342 nM ALK5 inhibitor (TGFβ type I receptor kinase inhibitor, ALX-270-445, Enzo Life Sciences), 15 μM oxytocin acetate (4016373 or old H-2510, Bachem) and 20 nM rapamycin (R8781, Sigma-Aldrich) were separately added to the testing medium for 4 days. Drugs were administrated in three scenarios: together with OCM for 4 days in non-treated WAT MPS (OCM+drugs); together with OCM for 4 days in 4-day OCM-pretreated WAT MPS (OO+drugs); and in plain HPLM without old serum for 4 days in 4-day OCM-pretreated WAT MPS (Dilution+drugs). Vehicle conditions were set as OCM for 4 or 8 days without drug. All treatments started on day 14.

### siRNA knockdown in WAT MPS

WAT MPSs were transfected with siRNAs using Lipofectamine RNAiMAX (13778075, ThermoFisher) according to manufacturer protocol. Briefly, 50 pmol of siRNA in 50 μl Opti-MEM (31985070, ThermoFisher) was mixed with 50 μl Opti-MEM containing 1.25 μl Lipofectamine RNAiMAX and incubated for 20 min at room temperature. The mixture was then diluted to 500 μl, yielding a final siRNA concentration of 100 nM, consistent with manufacturer recommendation (Cell Signaling Technology). The transfection medium was perfused into the WAT MPS at a flow rate of 20 μl h^−1^ overnight. Transfection efficiency was validated using control siRNA with fluorescein conjugation (6201, Cell Signaling Technology) in a trial test. For experimental conditions, control siRNA (6568, Cell Signaling Technology) or CDKN2A/p16 siRNA (6598, Cell Signaling Technology) were used in parallel transfections of WAT MPSs derived from the same batch. Before transfection, WAT MPSs were rinsed with Opti-MEM for 5 min. WAT MPSs at day 14 were used for attenuation of aging induction, while WAT MPSs at day 12 were used for rejuvenation, ensuring that the final collection day was the same across conditions.

### Functional assays for WAT and liver in MPS

For the basic lipid accumulation assay, low-glucose DMEM (1 g l^−1^ glucose, 11885084, Gibco) with 2 μM fluorescent fatty acids (Bodipy 500/510 C1, C12, D3823, ThermoFisher) was infused in MPSs at 37 °C for 2 h. After rinsing, fatty acids within MPS chambers were quantified on the basis of the relative fluorescence unit (RFU) of the respective MPS. For adipogenic glucose uptake, WAT MPSs were infused with low-glucose DMEM at 37 °C for 2 h for basal uptake, and then with 1 μM insulin (I2643, Sigma-Aldrich) for an additional 2 h for insulin-stimulated uptake. For hepatic glucose production, liver MPSs were infused with low-glucose DMEM with 2 mM sodium pyruvate (11360070, Gibco), 10 mM sodium lactate (L7022, Sigma-Aldrich) at 37 °C for 2 h for basal production, and then including 100 nm insulin for 2 h for insulin-inhibited production. Glucose content in infusion medium was measured using the Amplex Red Glucose/Glucose Oxidase Assay kit (A22189, ThermoFisher). For cytokine TNF secretion, circulating media were analysed by ELISA (KHC3011, ThermoFisher) and assessed relative to a serum-free control condition, with negative values indicating comparatively reduced production. For DNA damage and P16 expression, cell lysates in RIPA buffer (89901, ThermoFisher) were measured by 8-OHdG ELISA (IT7974, G-Biosciences) and P16 ELISA (ab227903, Abcam), then normalized to protein concentration (BCA Protein Assay kit, 23225, ThermoFisher). All kits were used according to manufacturer instructions. Absorbance and fluorescence of assay solutions were analysed using a monochromator-based microplate reader (SpectraMax i3×, Molecular Devices).

### Immunofluorescence, SA-β-gal staining and image acquisition

MPSs were fixed using 4% paraformaldehyde (15710, Electron Microscopy Sciences) for 2 h. Senescent cells were labelled using a β-galactosidase staining kit (9860, Cell Signaling Technology) following manufacturer instruction, using a consistent final pH of 6.0 (Seven Compact S210, Mettler Toledo). For immunostaining, non-specific binding was blocked in PBS containing 1% BSA (A8806, Sigma) overnight. Permeabilization was activated using 1% saponin (558255, Sigma) for 1 h, and then maintained until final rinse. Human P16 was sequentially stained with 1% human anti-p16 (rabbit recombinant anti-CDKN2A/p16INK4a, ab108349, Abcam) overnight, and then with 0.2% anti-rabbit IgG conjugated to Alexa Fluor 647 (B40926, ThermoFisher) for 2 h. Nuclei and lipid droplets were stained overnight using 300 nM DAPI (D1306, ThermoFisher) and 1 μM fatty acid dye (Bodipy 493/503, D3923, ThermoFisher). Samples were mounted using Diamond Antifade (P36965, Invitrogen) after staining and rinsing. Brightfield and fluorescence images were captured by a wide-field fluorescence microscope (EVOS M5000 Imaging System, ThermoFisher) and confocal fluorescence microscopes (LSM710, LSM900, Zeiss). To enable fluorescence comparison, all staining and imaging procedures were kept consistent. Zen (Zeiss) and ImageJ (no plug-ins) were used to analyse colorimetric (SA-β-gal) or fluorescence intensity (p16) with consistent settings. Imaging analyses were based on whole cell chambers (under ×10 objective lens, 1 chamber as one data point, 8 chambers per MPS).

### RNA isolation from MPS and gene expression

Cells were exposed by cutting and exfoliating the cell chamber slab off the device, and then lysing in Trizol (15596026, Invitrogen). RNA isolation (T2010S, NEB), synthesis of cDNA (Maxima First Strand cDNA Synthesis kit, K1641, ThermoFisher) and qPCR (*Taq*Man Universal PCR Master Mix, 4304437, ThermoFisher) in QuantStudio5 (Applied Biosystems, ThermoFisher) were done according to manufacturer instructions. Primers (Integrated DNA Technologies) for qPCR are listed in Supplementary Table 3. All genomic analyses were done within 2 weeks of collection.

### De novo proteomes

WAT MPSs derived from the MetRS^L274G^ hiPSC WTC11 line were differentiated and treated following the same protocol. Azidonorleucine (ANL, 2 mM) was added to the medium at the onset of YCM and OCM treatments to label newly synthesized proteins. After cell lysis in RIPA buffer, ANL-labelled proteins were conjugated to alkyne-Cy3 using the Click-iT Protein Reaction Buffer kit (C10276, ThermoFisher) and subsequently probed on a human antibody array targeting 507 serum proteins (Human L507 Array, RayBiotech Life) according to manufacturer instructions. Arrays were scanned at 532 nm using a GenePix 4000B scanner (Molecular Devices) and analysed with GenePix Pro 6.1 software. De novo proteins secreted to the effluent medium were also detectable using dialysis, but the signal tended to be more variable between experiments.

Data processing included background correction using positive and negative control probes with an offset to stabilize low-intensity values, normalization to a common reference array as specified in the RayBio L-Series Human Antibody Array user manual, and appropriate averaging of technical and biological duplicates. Further data handling was performed with the limma package in R, with supplementary organization in Python using Pandas and Numpy.

### Statistics and reproducibility

All results were statistically analysed in Prism 10 (GraphPad). All experiments contain 3 or more biological replicates (*n*) for each group. Paired two-tailed *t*-test with assumption of equal variance was used to compare differences between two groups from the same MPS. Unpaired two-tailed *t*-test with the same assumption was used for results from different MPSs. One-way analysis of variance (ANOVA) for comparison of 3 or more groups with one testing variable and two-way ANOVA for comparison of 3 or more groups with two testing variables, followed by post hoc tests for multiple comparisons with corrections were performed to determine significant pair(s): Dunnett’s test was used to determine the significance of differences between drug treatments and old control (Fig. 6a,b), Šídák’s test to determine cross-group differences for each condition (Fig. 2e), and Tukey’s test to determine all significant pairs (Fig. 6c). Detailed test methods, *P* values, degrees of freedom, confidence intervals and exact sample sizes (*n*) for all results are listed in Supplementary Data 1. Exact *P* values are labelled in figures for comparison pairs with *P* < 0.1. Results are shown as mean values with error bars representing standard deviation. All experiments were validated by independently repeating at least 3 times with similar results.

### Bioinformatics analyses methods

Bulk RNA-seq RNA samples collected from MPS were frozen and shipped in dry ice to Novogene for RNA sequencing. Libraries were sequenced on Illumina after quality control via Qubit and qPCR. Alignments were performed with HISAT2 (ref. ^75^) software by Novogene. Subsequent analyses of gene expression were carried out using Python (3.10.13) with methods described below.

#### Differential expression analysis

RNA-seq gene counts were first normalized using the median of ratios, which is the default normalization method of the popular differential expression analysis package DESeq2 (ref. ^76^). For 4-day serum treatment samples, we also performed an additional ‘within batch normalization’ step, in which we normalized the gene counts of each sample to the control sample of the corresponding batch, to reduce batch effects of WAT MPS before proceeding to differential expression analysis (DEA).

DEA was conducted with pyDESeq2 (ref. ^77^), a Python implementation of DESeq2. Since normalization had already been performed using the method described above, the built-in normalization step in the pyDESeq2 pipeline was omitted. Differentially expressed genes (DEGs) were identified with the Wald test using pyDESeq2 (with threshold false discovery rate (FDR) ≤ 0.05 and absolute log_2_(fold change) ≥ 1) after multiple testing correction using the Benjamini–Hochberg method. Visualization of DEGs was performed with Matplotlib (3.8.3), Seaborn (0.13.0) and Scipy (1.11.3). Identification of aging-associated genes was based on the public databases of HAGR (Human Ageing Genomic Resources).

#### Differential variability analysis of gene expression

On the basis of the same normalized datasets utilized in DEA, we identified gene markers that exhibited a significant increase in variability, or biological noise, with age, also known as increasingly variably expressed genes (IVEGs), in the GTEx Adipose Subcutaneous and Liver databases as well as our WAT-MPS and liver-MPS RNA-seq data using Levene’s test with a significance threshold of *P* < 0.05 (that is, with unequal variance, and a log_2_ fold change of the coefficient of variation (CV) > 0, calculated as log_2_(CV_old_/CV_young_), that is, old CV is larger than young CV). CV was used to account for relative variation regarding mean expression values, acknowledging that higher means may exhibit greater variation and vice versa. Similarly, aging-associated genes were selected on the basis of HAGR. An advanced threshold with log_2_(CV_old_/CV_young_) > 0.5 was tested to further identify the core and mostly dysregulated genes. Data analysis and statistical tests were conducted using Python with the packages Pandas, Scikit-learn (1.3.2)^68^ and SciPy (1.11.3)^78^.

#### Gating threshold for liver samples in GTEx

For cross-comparison with physiological aging using the GTEx database, we initially aimed to match the same age ranges for young (20–29) and old (60–69) cohorts to serum donors as in the WAT analysis. However, the liver database presented limitations, including small sample sizes and uneven distributions (161 males and 65 females in the age range 20–79), and importantly, disease-biased phenotypes (for example, only 7 samples with high inflammation in the 20–29 cohort). To balance biases and statistical power, differential genes (DEGs) and variables (IVEGs) for liver aging were adjusted to compare 30–39 vs 50–69 age groups, with a lowered significance threshold (*P* < 0.05 instead of FDR < 0.05). To compensate for sex-related variances in DEA, the selection threshold was lowered to log_2_(fold change) ≥ 0.5.

#### Principal component analysis

Principle component analysis (PCA) was conducted on log-transformed normalized gene counts using Numpy (1.26.1) and Scikit-learn (1.3.2)^78^, and then visualized using Matplotlib (3.8.3). Ellipses in the PCA plots indicate 95% confidence intervals.

#### Enrichment analysis

Enrichment analyses were performed using both DAVID^79,80^ and SRplot^81^ based on the differential genes (DEGs) and variables (IVEGs). The two web-based tools yielded similar enriched terms, and we extracted the terms enriched from categories including Gene Ontology and KEGG pathways for further analysis and visualization. After Benjamini–Hochberg correction, terms and pathways that were enriched, with threshold adjusted *P* ≤ 0.05, were selected on the basis of biological functions and plotted in Python using Matplotlib (3.8.3) and Seaborn (0.13.0).

#### STRING network analysis

The STRING Data Resource (v.12.0)^82,83^ was used for the protein–protein interaction (PPI) networks. A medium confidence score of 0.4 (default) was applied as threshold to identify significant interactions. The STRING-enriched terms (consistent with DAVID enrichment) were incorporated into the PPI visualization. Singleton genes away from the PPI network were hidden for clarity. In our PPI prediction by STRING, we observed that the result from the latest version (v.12, updated in July 2023) was inconsistent with the previous 10 years of versions, ranging from v.9.1 (released in December 2013) to v.11.5 (archived in July 2023), as detailed in Supplementary Table 4. For higher confidence score and consistency with studies in the past decade, v.11.5 was used in prediction.

#### Transcriptome-based age-group prediction model

To assess our aging-induction outcomes, we obtained bulk RNA-seq data of SAT and VAT from the GTEx databases, which is by far the largest human bulk RNA-seq project^32^, and normalized the acquired data of each depot with the median of ratios method from DESeq2. We then split the normalized data by sex, which resulted in 445 male SAT samples and 218 female SAT samples, and 371 male VAT samples and 170 female VAT samples. For each sex and fat depot, genes with fewer than 100 total counts were filtered out. Mitochondrial genes were also excluded to reduce model complexity. A log transformation was applied to the remaining genes. Next, highly variable genes were selected on the basis of the variance-to-mean ratio and the mean expression level of each gene, yielding 530 genes in male SAT, 267 genes in female SAT, 697 genes in male VAT and 309 genes in female VAT. Dimensionality was further reduced using linear discriminant analysis (LDA) in Scikit-learn. To classify each sample to the age of its donor, *k*-nearest neighbours (kNN)-based classifiers imported from Scikit-learn were trained on the LDA-transformed data, using 70% randomly selected samples of each sex and depot as training set, with 6 age groups (20–29, 30–39, 40–49, 50–59, 60–69, 70–79) as prediction outcomes. The trained age-group prediction models were then validated using the remaining 30% of the samples (Extended Data Fig. 4a). In subsequent iteration, the classification function was updated to include three algorithm options: random forest, KNN and support vector machine (SVM). The model achieving the best performance during cross-validation on the GTEx database was the random forest model, which was selected for downstream prediction of MPS ages and shared in GitHub. Other classification methods, multilayer perceptron (MLP) and XGBoost, were also tested during the prototyping stage, but they exhibited signs of overfitting and performed poorly in cross-validation.

When applying the prediction model to our MPS, we first preprocessed the RNA-seq data using the same steps as in the GTEx. Next, we extracted the highly variable genes identified during the model training process and applied the same LDA model to reduce dimensionality. The LDA-transformed data were used as input for prediction. To visualize the prediction outcome, we created a scatterplot (Matplotlib, 3.8.3) using LD1 and LD2 from the LDA output.

#### Partial least squares discriminant analysis

Partial least squares discriminant analysis (PLS-DA)^84^ is a multivariable statistical technique to classify samples on the basis of their gene expression and to reveal biological mechanisms^85,86^. We began by normalizing the samples with the median of ratios method, and then applied a standard scaler to minimize bias towards genes with higher expression. We identified the major contributors to the latent variable that best distinguished the two conditions, selecting those with an absolute weight threshold of ≥0.01, which typically yielded the top 1,000 genes. Selected major contributor genes were then ranked on the basis of their log2 fold changes across the two conditions in the comparison. Up and down-altered major contributor genes were subsequently used for enrichment analyses.

### Reporting summary

Further information on research design is available in the Nature Portfolio Reporting Summary linked to this article.

## Supplementary information

Supplementary Information Supplementary Methods 1 and 2, Fig. 1 and Tables 1–4.

Reporting Summary

Peer Review File

Supplementary Data 1 Detailed statistical test methods, *P* values, degrees of freedom, confidence intervals and exact sample sizes (*n*) for all results.

Supplementary Data 2 Bioinformatic statistical source data (including raw and meta data) for all main figures.

Supplementary Data 3 Bioinformatic statistical source data (including raw and meta data) for all extended data figures.

## Source data

Source Data Figs. 1–6 and Extended Data Fig. 1 Statistical source data.

## Data Availability

Serum donor information and qPCR primer sequences are provided in Supplementary Tables 2 and 3. Sample sizes, statistical methods and *P* values are provided in Supplementary Data 1. Transcriptomic raw FASTQ data are available at the Gene Expression Omnibus GSE280361 for all WAT MPSs and GSE309980 for all liver MPSs. Source data are provided with this paper.

## Code availability

The age prediction model code is shared on GitHub at https://github.com/yuchen-he2000/WAT-MPS-aging (ref. ^87^).
