# Supplementary material for: Human microphysiological systems of aging recreate the in vivo process expediting evaluation of anti-geronic strategies
Source: Nat Biomed Eng. 2026 Mar 25;10(10):2195–212. doi: 10.1038/s41551-026-01618-6 (PMC13672102; doi:10.1038/s41551-026-01618-6)
Supplement: Supplementary file 1 [file 41551_2026_1618_MOESM1_ESM.pdf]

nature biomedical engineering

Supplementary information

https://doi.org/10.1038/s41551-026-01618-6

Human microphysiological systems of aging recreate the in vivo process expediting evaluation of anti-geronic strategies

In the format provided by the authors and unedited

Supplementary Info for *"Human microphysiological systems of aging recreates the in vivo process expediting evaluation of anti-gerontic strategies"*

Table of Content:

Supplementary Method 1: MetRS^{L274G} hiPSCs line establishment

Supplementary Method 2: time-course pilot test of OCM induced aging.

Supplementary Figure 1. All gene expression by qPCR, including nonsignificant.

Supplementary Table 1. Comparison to animal alternative aging models

Supplementary Table 2. Biometric information of serum donors

Supplementary Table 3. Primers for qRT-PCR.

Supplementary Table 4. Prediction differences over different versions of STRING.

Supplementary Data 1. All detailed statistic reports and samples size (n) are provided in separate excel file "Supplementary_Data_1"

Supplementary Method: MetRS^{L274G} hiPSCs line establishment

Human iPSCs WTC11 (GM25256 hPSCreg: UCSFi001-A) were maintained on vitronectin-coated plates in Essential 8 medium (Thermo Fisher) and transduced at ~60–70% confluence with custom lentiviral cDNA expression construct encoding hMARS_L274G (MetRS^{L274G}, RefSeq#: NM_004990.4, Gene ID: 4141) together with TagRFP and a puromycin resistance cassette (pRCDCMURP-CMV-hMars_L274G-UbiC-TagRFP-2A-Puro, packaged lentivirus, Lot#231127004, Cellecta, CA, USA). Viral supernatant (2×10^{6} TU/mL, 500 µl) was mixed 1:1 with culture medium containing polybrene (5 µg/ml) and applied for 6 h, followed by dilution with fresh medium and incubation for a total of 24 h. Puromycin selection was initiated 48–72 h post-transduction, and TagRFP fluorescence was used to monitor transduction efficiency and enrich positive colonies. Stable integration and expression of MetRS were confirmed by fluorescence microscopy, with clonal colonies hand-picked for expansion. All lentiviral manipulations were performed under BSL-2 conditions, with waste decontaminated in 10% bleach.

Full plasmid construct attached below:

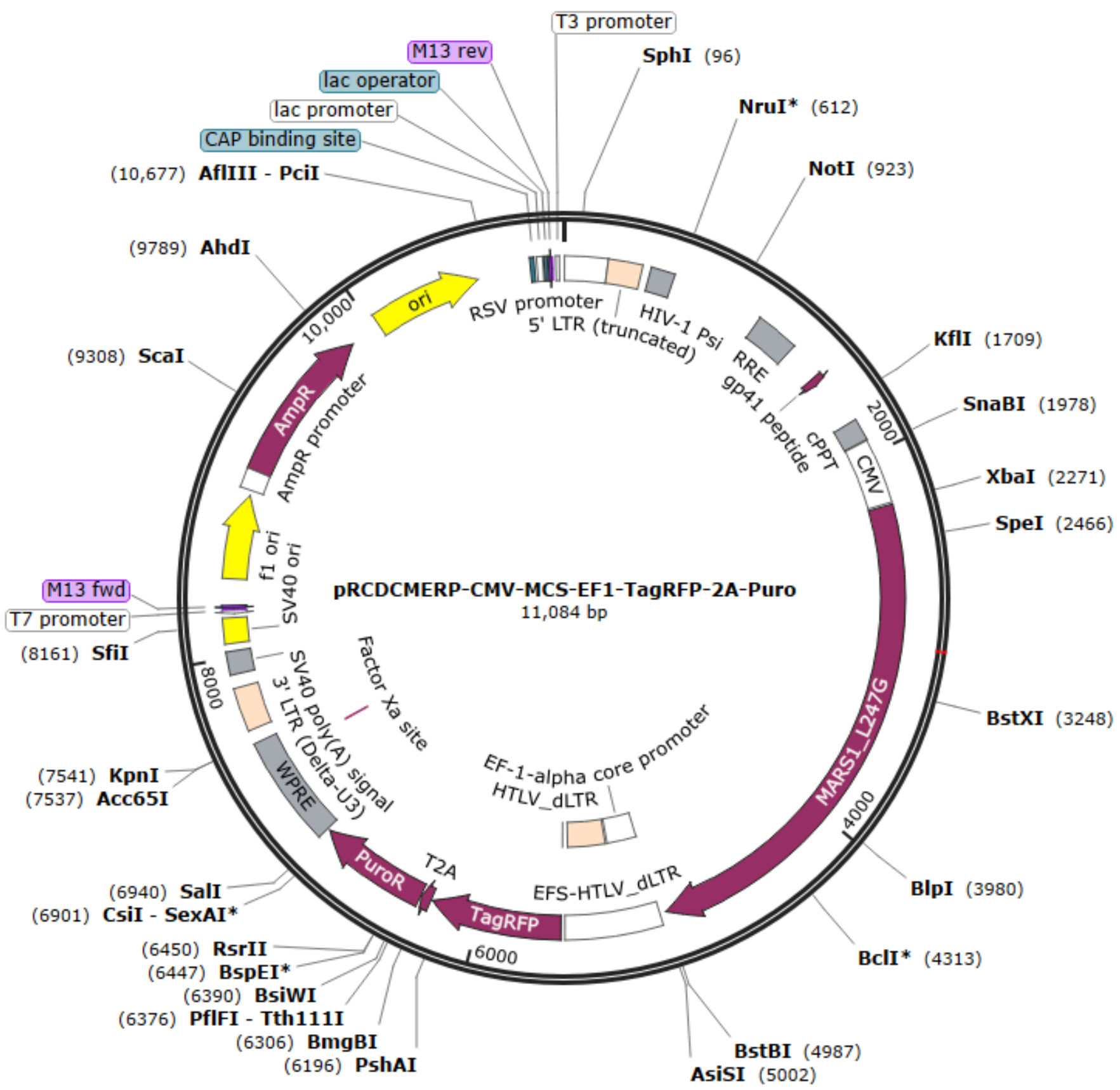

Supplementary Method 2: time-course pilot test of OCM induced aging: The same batch of WAT-MPSs was treated with OCM (HPLM supplemented with 5% pooled male serum) for 0-4 days. Aging status was assessed by senescence-associated β-galactosidase staining. n=4 for all. n referring to one chamber of MPS. One-way ANOVA followed by Dunnett's test was performed to determine the critical time point for senescence induction. Exact p values <0.1 were directly noted on the compared pairs. Data are presented as mean values +/- SD for all bar graphs.

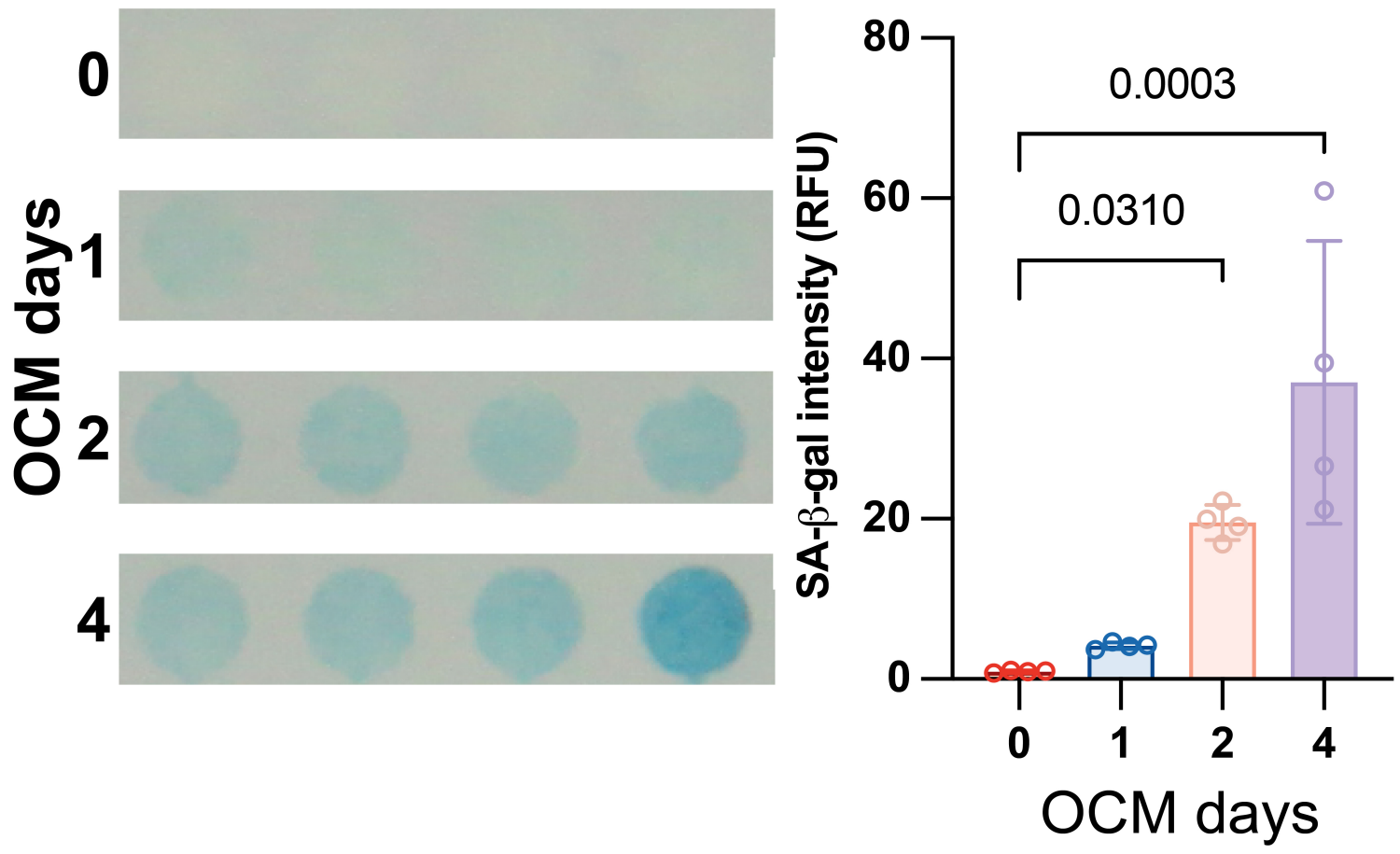

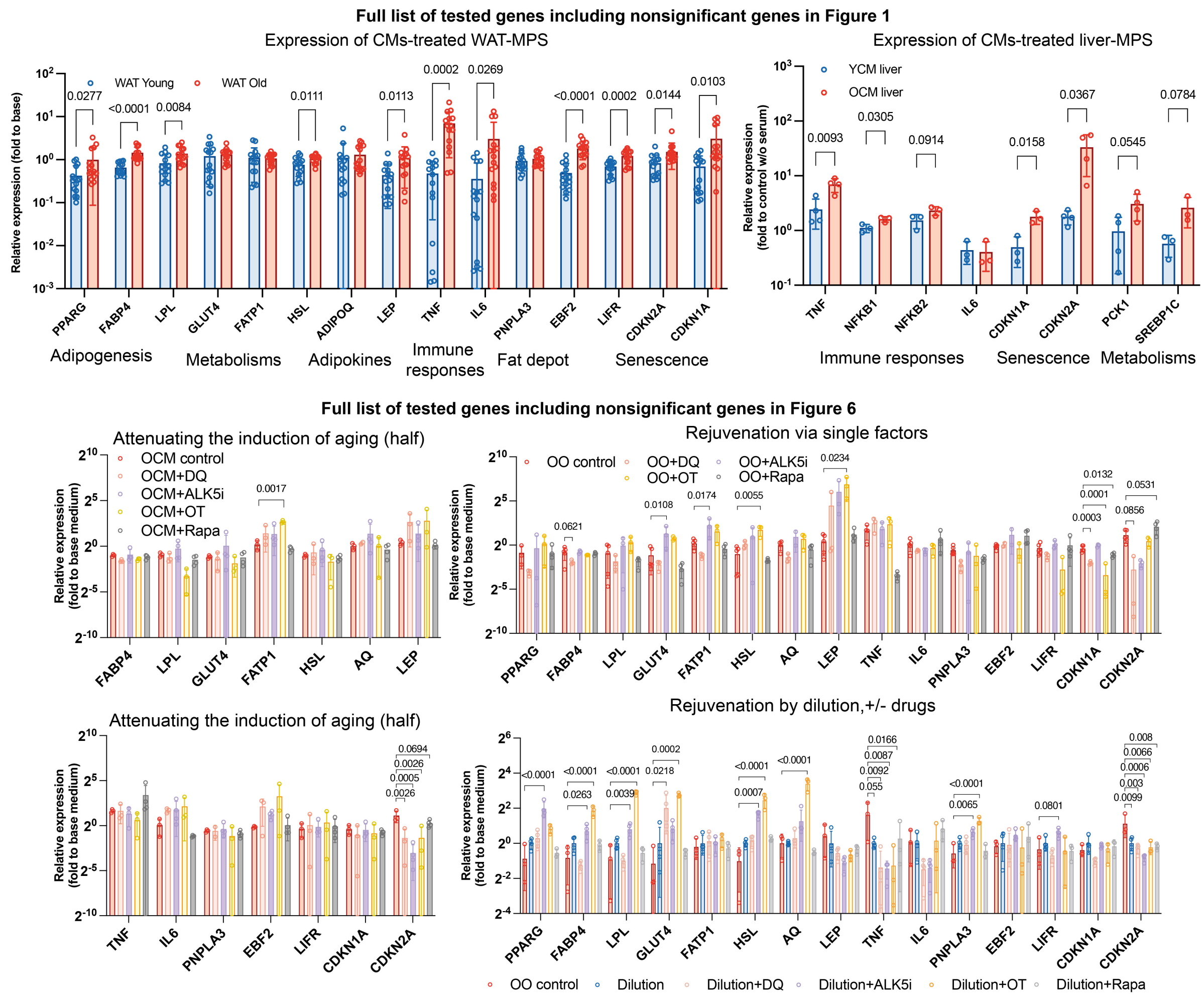

Supplementary Figure 1.

All gene expression by qPCR, including nonsignificant pairs in Figure 1: n=15 for CMs-treated WAT-MPS; n=4 for TNF, CDKN2A and PCK1 otherwise n=3 for CMs-treated liver-MPS. p values were determined by unpaired two-tailed t-tests. All gene markers were normalized to negative control, which was cultured in the same batch with the conventional MPS medium.

All gene expression by qPCR, including nonsignificant pairs in Figure 6: n=3 for all OCM control, OCM+DQ, OCM+OT and n=4 for all OCM+ALK5i and OCM+Rapa, excepting for ADIPOQ and PNPLA3 of OCM+DQ (n=2), TNF of OCM+ALK5i (n=3) in the Attenuating the induction of aging; n=3 for all OO+DQ, OO+ALK5i, OO+OT excepting EBF2 of OO+DQ, OO+ALK5i, OO+OT and ADIPOQ OO+ALK5i (n=2), n=4 for all OO+Rapa, n=5 for all OO control excepting PPARG (n=3), ADIPOQ and TNF (n=4) in the Rejuvenation via single factors. n=3 for all OO control, Dilution+OT, Dilution+Rapa, and n=4 for others in Rejuvenation by dilution +/- drugs. p-values in comparison of each gene in the dilution settings were calculated by one-way ANOVA followed by Tukey's test, otherwise by one-way ANOVA followed by Dunnett's test. All genes in the dilution settings were normalized to the dilution group, otherwise to the first group.

For all figures, n referring to MPS. Exact p values <0.1 were directly noted on the compared pairs. Data are presented as mean values +/- SD for all bar graphs.

Supplementary Table 1. Comparison to animal alternative aging models

Publication	Cell source	Cell scalability (pluripotency)	Isogenic coculture	Sexual dimorphic aging	Modeling tissue types	Aging induction method	Aging hallmarks
Ao, Z., et. al. 2022. *Advanced Science*, *9*(27), p.2200475.	hESC-derived brain organoid and human primary monocytes	Partially	No	N/A	Brain-Immune	senescent immune cells from old donors	Gene expression, SASP secretion
Bersini, S.,et. al. 2020. Advanced Biosystems, 4(5), p.2000044.	Human primary endothelial and fibroblast	No	No	N/A	Vascular	Donor's natural aging, human serum for 14 and 25 days	Gene expression, immunostaining, transcriptomics, function of permeability
Pauty, J., et. al. 2021. Biomaterials Science, 9(1), pp.199-211.	HUVEC, and human primary fibroblasts	No	No	N/A	Vascular	Irradiation-induced senescent fibroblasts	Gene expression, immunostaining, SASP secretion, functions of permeability and contraction
Giza, S., et. al. 2022. Aging Cell, 21(7), p.e13650.	Human primary myogenic cells	No	N/A	N/A	Muscle	Donor's natural aging	Gene expression, myotube morphology, function of contraction
Budhathoki, S., et. al. 2022. *Cells Tissues Organs*, *211*(3), pp.348-359.	Rat immortalized myoblast cell line	Yes	N/A	N/A	Muscle	doxorubicin, hypoxia	Gene expression, DNA damage and ROS staining, immunostaining, nuclei and cell morphology
Lee Y., et al. 2020. Adv. Funct. Mater., 30, 2002924	Mouse immortalized lines of myoblasts and endothelial cells, primary myogenic cell	Yes	No	N/A	Muscle-Vascular	Mice serum, oxidative stress	Gene expression, immunostaining, SASP secretion, myotube morphology
Our study	hiPSC-derived adipocyte and hepatocytes	Yes	Yes	Yes	Fat-Liver	Human serum for 4 days, serum-induced senescent adipocytes	Gene expression, b-gal staining, immunostaining, DNA damage, SASP secretion, glucose and lipid metabolism, and other aging hallmarks shown in transcriptomics. Detailed info in the Supplementary Table 2

Supplementary Table 2. Biometric information of serum donors. **BOLD** font labels the sera that were individually tested for the universality of serum-induced effects in WAT-MPS. After confirming, pooled serum was made by mixing equal volume of the rest of five sera in each condition.

Age and sex	Donor ID	Age	Age and sex	Donor ID	Age
young male	**354383631**	**28**	old male	**352526310**	**66**
	**354383556**	**29**		**390024399**	**74**
	354383654	33		MET0015	70
	350339778	33		MET0020	70
	354383653	32		MET0021	70
	350339460	32		MET0006	80
	370094035	33		MET0040	82
young female	**354383670**	**21**	old female	**390024897**	**73**
	**354383489**	**21**		**350339479**	**82**

Supplementary Table 3. Primers for qRT-PCR.

Gene	Direction	Sequence	Gene	Direction	Sequence
PPIA	Forward	GTGGCGGATTTGATCATTTGG	*GLUT4 (SLC2A4)*	Forward	TCCAACAGAT AGGCTCCGAA
	Reserve	CAAGACTGAGATGCACAAGTG		Reserve	CCCAATGTTGTACCCAAACTG
TNF	Forward	CCTCTCTCTAATCAGCCCTCTG	*FATP1 (SLC27A 1)*	Forward	AGTGTCTCATCTATGGGCTGA
	Reserve	GAGGACCTGGGAGTAGATGAG		Reserve	AGGTAGCGGCAGATCTCC
NFKB1	Forward	CAGGAGACGTGAAGATGCTG	*HSL (LIPE)*	Forward	AGTATGTCACGCTGCATAAGG
	Reserve	AGTTGAGAATGAAGGTGGATGA		Reserve	GAAGGACACCAGCCCAAT
NFKB2	Forward	ATGGAGAGTTGCTACAACCCA	*ADIPOQ*	Forward	CCTCACTTCCATTCTGACTGC
	Reserve	CTGTTCCACGATCACCAGGTA		Reserve	AGCATCCTGAGCCCTGAT
PCK1	Forward	TCATTTAAGGGCCATCAACC	*LEP*	Forward	TGGCTTCCAGGTATCTCCA
	Reserve	CTCATCAATGCCTTCCCAGT		Reserve	AGAGTGGCTTAGAGGAGTCAG
SREBP1c	Forward	CCATGGATTGCACTTTCGAA	*IL6*	Forward	GCAGATGAGTACAAAAGTCCTGA
	Reserve	GGCCAGGGAAGTCACTGTCTT		Reserve	TTCTGTGCCTGCAGCTTC
CDKN1A	Forward	GCAGACCAGCATGACAGAT	*PPARG*	Forward	GTTTCAGAAATGCCTTGCAGT
	Reserve	GAGACT AAGGCAGAAGATGTAGAG		Reserve	GGATTCAGCTGGTCGATATCAC
CDKN2A	Forward	CCAACGCACCGAATAGTTACG	*FABP4*	Forward	ACTTGTCTCCAGTGAAAACTTTG
	Reserve	CGCTACCTGATTCCAATTCC		Reserve	ATCACATCCCCATTCACACT
BCL6	Forward	AGAAGCCCTATCCCTGTGAA	*LPL*	Forward	AGTCAGAGCCAAAAGAAGCAG
	Reserve	GACGGAAATGCAGGTTACACT		Reserve	TTGGTATGGGTTTCACTCTCAG
LIFR	Forward	TTATTGCCATTCTCATCCCAGT	*PNPLA3*	Forward	CCTTGGTATGTTCCTGCTTCA
	Reserve	T’TGGAATATCAGGGTAGAAGGTT		Reserve	GTTGTCACTCACTCCTCCATC
			EBF2	Forward	CTATGTCAGGCTCATCGACTC
				Reserve	CAGCATCGACT ACACATCACT

Supplementary Table 4. Differences in the prediction of 10 partner genes by different versions of STRING. **BOLD** font labels shared genes in v9.1. Green front labels confidential score >or = 0.999; orange for score < 0.99; red for score < 0.90.

The 10 genes predicted by v12 have significantly lower confidence scores, with 9 of them scoring below 0.99 (including 4 of them below 0.9), and none overlapping with predicted genes from the earlier versions. In contrast, versions from v9.1 to v11.5 are consistent, with all predicted genes having confidence scores above 0.99, and pathways enriched in mTOR signaling/longevity and insulin signaling, none of which were enriched by v12.

STRING Versions	v9.1	v10a	v10.5	v11	v11.5	v12
Number of identifiable genes by STRING	31	31	33	33	33	34
Predicted 10 partner genes in different versions	**MTOR**	**MTOR**	**TSC2**	**MTOR**	**MTOR**	SLURP2
	**TSC2**	**TSC2**	MRPL1	**TSC2**	**TSC2**	KCNMB2
	**INSR**	**INSR**	**INSR**	RPTOR	**PIK3R1**	AGRP
	**PI3KR1**	**PIK3R1**	MRPL4	MRPL27	**CNTF**	PRR20C
	**CNTF**	**CNTF**	MRPL27	MRPS7	**GRB2**	KNMB3
	F10	**IGF1R**	MRPL17	MRPL4	RPTOR	CLCF1
	IRAK1	**GRB2**	MRPL36	MRPS9	TSC1	OGFR
	**IGF1R**	RPTOR	MRPL32	MRPL17	BNIP3	TLE7
	**GRB2**	TSC1	MRPL34	MYH9	MRPL27	HYLS1
	INS	SHH	MRPL20	MRPL32	MRPL4	GPC4
Whether below two key pathways can be enriched from the 37 plus 10 predicted genes?						
mTOR/longevity	YES	YES	YES	YES	YES	NO
Insulin signaling	YES	YES	YES	YES	YES	NO

We believe this inconsistency is primarily due to v12.0 being the first or alpha subversion of v12 thus less reliable, considering the similar issue occurred on v11 as well. Additionally, v12 has a reduced protein database (59,309,604 proteins from 12,535 organisms) compared to v11.5 (67,592,464 proteins from 14,094 organisms), see below screenshot of version history. To ensure reliability, greater predictability, and consistency with those studies involved STRING analysis from the past decade, we decided to use v11.5, the third subversion of v11,

to predict 10 biomarkers from the 37 noise markers. While the PPI network was plotted using v12 (27,541,372,833 interactions) due to its inclusion of more interactions than v11.5 (20,052,394,042 interactions).

STRING database version history

STRING Database Version History

Version	Date	Address	Content
12.0	current: since July 26, 2023	https://string-db.org/	59'309'604 proteins from 12535 organisms; 27'541'372'833 interactions.
11.5	August 12, 2021 to July 26, 2023	https://version-11-5.string-db.org/	67'592'464 proteins from 14094 organisms; 20'052'394'042 interactions.
11.0b	October 17, 2020 to August 12, 2021	https://version-11-0b.string-db.org/	24'584'628 proteins from 5090 organisms; 3'123'056'667 interactions.
11.0	January 19, 2019 to October 17, 2020	https://version-11-0.string-db.org/	24'584'628 proteins from 5090 organisms; 3'123'056'667 interactions.
10.5	May 14, 2017 to Jan 19, 2019	https://version-10-5.string-db.org/	9'643'763 proteins from 2031 organisms; 1'380'838'440 interactions.
10.0	Apr 16, 2016 to May 14, 2017	http://version10.string-db.org/	9'643'763 proteins from 2031 organisms; 932'553'897 interactions.
10.0a	Apr 12, 2105 to Apr 16, 2016	http://version10a.string-db.org/	9'643'763 proteins from 2031 organisms; 932'553'897 interactions.
9.1	Dec 27, 2013 to Apr 12, 2015	http://string91.embl.de/	5'214'234 proteins from 1133 organisms; 332'235'675 interactions.
