# Supplementary material for: Human microphysiological systems of aging recreate the in vivo process expediting evaluation of anti-geronic strategies
Source: Nat Biomed Eng. 2026 Mar 25;10(10):2195–212. doi: 10.1038/s41551-026-01618-6 (PMC13672102; doi:10.1038/s41551-026-01618-6)
Supplement: Supplementary file 2 [file 41551_2026_1618_MOESM2_ESM.pdf]

nature portfolio

Corresponding author(s): Irina Conboy, Andreas Stahl

Last updated by author(s): Dec 8, 2025

Reporting Summary

Nature Portfolio wishes to improve the reproducibility of the work that we publish. This form provides structure for consistency and transparency in reporting. For further information on Nature Portfolio policies, see our Editorial Policies and the Editorial Policy Checklist.

Statistics

For all statistical analyses, confirm that the following items are present in the figure legend, table legend, main text, or Methods section.

n/a	Confirmed	
☐	☒	The exact sample size (*n*) for each experimental group/condition, given as a discrete number and unit of measurement
☐	☒	A statement on whether measurements were taken from distinct samples or whether the same sample was measured repeatedly
☐	☒	The statistical test(s) used AND whether they are one- or two-sided<br>*Only common tests should be described solely by name; describe more complex techniques in the Methods section.*
☒	☐	A description of all covariates tested
☐	☒	A description of any assumptions or corrections, such as tests of normality and adjustment for multiple comparisons
☐	☒	A full description of the statistical parameters including central tendency (e.g. means) or other basic estimates (e.g. regression coefficient) AND variation (e.g. standard deviation) or associated estimates of uncertainty (e.g. confidence intervals)
☐	☒	For null hypothesis testing, the test statistic (e.g. *F*, *t*, *r*) with confidence intervals, effect sizes, degrees of freedom and *P* value noted<br>*Give P values as exact values whenever suitable.*
☒	☐	For Bayesian analysis, information on the choice of priors and Markov chain Monte Carlo settings
☒	☐	For hierarchical and complex designs, identification of the appropriate level for tests and full reporting of outcomes
☒	☐	Estimates of effect sizes (e.g. Cohen's *d*, Pearson's *r*), indicating how they were calculated

Our web collection on statistics for biologists contains articles on many of the points above.

Software and code

Policy information about availability of computer code

Data collection	Excel (Microsoft 365), QuantStudio Design and Analysis (v1.5.1, Applied Biosystems), Zen-black (2.3 SP1 FP3 64bit, Zeiss), SoftMax Pro (v6.3, Molecular Devices), EVOS M5000 Microscope Imaging System
Data analysis	Quantstudio Real-Time PCR software v1.3, Zen-blue (3.4 version, used as downloaded), ImageJ (1.53a, used as downloaded, no plug-ins or macros add-on), Prism 10 (Graphpad)

For manuscripts utilizing custom algorithms or software that are central to the research but not yet described in published literature, software must be made available to editors and reviewers. We strongly encourage code deposition in a community repository (e.g. GitHub). See the Nature Portfolio guidelines for submitting code & software for further information.

Data

Policy information about availability of data

All manuscripts must include a data availability statement. This statement should provide the following information, where applicable:

- Accession codes, unique identifiers, or web links for publicly available datasets
- A description of any restrictions on data availability
- For clinical datasets or third party data, please ensure that the statement adheres to our policy

Primer sequences are provided in Supplementary Tables 2. Sample sizes, statistic methods and p-values are provided in Supplementary_Data1_Statistic. Source data

are provided in supplementary file “Source_Data.xlsx”. Transcriptomic raw FASTQ data are available on public repository, Gene Expression Omnibus, GSE280361 for all WAT-MPSs, GSE309980 for all liver-MPSs. Age prediction model code is shared on Github via link: https://github.com/yuchen-he2000/WAT-MPS-aging.

Research involving human participants, their data, or biological material

Policy information about studies with human participants or human data. See also policy information about sex, gender (identity/presentation), and sexual orientation and race, ethnicity and racism.

Reporting on sex and gender	Female and male, detailed in the Supplementary Table 1 in the Supplementary Information.
Reporting on race, ethnicity, or other socially relevant groupings	Black, Sub-Saharan African, classified by the vendor (Metaphor Laboratory)
Population characteristics	Biological age of serum donors (young for ages 21-34, old for ages 62 and above) has been detailed in the Supplementary Table 1 in the Supplementary Information.
Recruitment	All human sera were from commercial vendors (Metaphor https://metaphorlaboratory.com/), i.e., no human subjects research was conducted, as per https://www.hhs.gov/ohrp/regulations-and-policy/guidance/research-involving-coded-private-information/index.html. as the samples had all identifying information removed by the vendors, meaning no one, including the researcher, can link the sample back to a specific individual, therefore they are not constituting "human subjects" under research regulations; essentially, the samples are anonymous and not associated with a living person.
Ethics oversight	Committee on Laboratory and Environmental Biosafety (CLEB), Stem Cell Research Oversight (SCRO) Office and Office of Environment, Health & Safety (EH&S) at University of California Berkeley

Note that full information on the approval of the study protocol must also be provided in the manuscript.

Field-specific reporting

Please select the one below that is the best fit for your research. If you are not sure, read the appropriate sections before making your selection.

☒ Life sciences ☐ Behavioural & social sciences ☐ Ecological, evolutionary & environmental sciences

For a reference copy of the document with all sections, see nature.com/documents/nr-reporting-summary-flat.pdf

Life sciences study design

All studies must disclose on these points even when the disclosure is negative.

Sample size	No statistical method was used to predetermine sample size. Sample size was chosen based on published examples minimizing assay-based errors, allowing for statistical analysis and ascertaining reproducibility of results.
Data exclusions	No data were excluded. MPS were excluded from analysis after technical failure during culture and/or microfluidic operation.
Replication	All experiments were repeated independently at least 3 times.. The exact number of biological replicates is summarized in Supplementary Data 1. All presented data are biological replicates from one of repeated experiments with the same conclusion. Technical replicates were performed for qPCR measurements to compensate for pipetting errors and averaged as one biological replicate.
Randomization	The study includes no experiments dependent on randomized allocation of samples into experimental groups.
Blinding	The study includes no experiments dependent on group allocation and blinding.

Reporting for specific materials, systems and methods

We require information from authors about some types of materials, experimental systems and methods used in many studies. Here, indicate whether each material, system or method listed is relevant to your study. If you are not sure if a list item applies to your research, read the appropriate section before selecting a response.

Materials & experimental systems

n/a	Involved in the study
☒	☐ Antibodies
☐	☒ Eukaryotic cell lines
☒	☐ Palaeontology and archaeology
☒	☐ Animals and other organisms
☒	☐ Clinical data
☒	☐ Dual use research of concern
☒	☐ Plants

Methods

n/a	Involved in the study
☒	☐ ChIP-seq
☒	☐ Flow cytometry
☒	☐ MRI-based neuroimaging

Eukaryotic cell lines

Policy information about cell lines and Sex and Gender in Research

Cell line source(s)	The healthy human male G15.AO iPSC line (RRID:CVCL_V192) was obtained from Barcelona Stem Cell Bank, Regenerative Medicine Program of IDIBELL. The human iPSC line GM25256 (WTC, hPSCreg: UCSFi001-A) was obtained from Bruce Conklin at the Gladstone Institute of Data Science and Biotechnology. Additional information can be found at https://www.coriell.org/O/Sections/Search/Sample_Detail.aspx? Ref=GM25256.
Authentication	Authenticated using SNP analysis.
Mycoplasma contamination	All cells were routinely tested for mycoplasma using Lonza MycoAlert Detection Kit and found to be negative.
Commonly misidentified lines (See ICLAC register)	No commonly misidentified cell lines were used in this study.

Plants

Seed stocks	*Report on the source of all seed stocks or other plant material used. If applicable, state the seed stock centre and catalogue number. If plant specimens were collected from the field, describe the collection location, date and sampling procedures.*
Novel plant genotypes	*Describe the methods by which all novel plant genotypes were produced. This includes those generated by transgenic approaches, gene editing, chemical/radiation-based mutagenesis and hybridization. For transgenic lines, describe the transformation method, the number of independent lines analyzed and the generation upon which experiments were performed. For gene-edited lines, describe the editor used, the endogenous sequence targeted for editing, the targeting guide RNA sequence (if applicable) and how the editor was applied.*
Authentication	*Describe any authentication procedures for each seed stock used or novel genotype generated. Describe any experiments used to assess the effect of a mutation and, where applicable, how potential secondary effects (e.g. second site T-DNA insertions, mosiacism, off-target gene editing) were examined.*
