# Supplementary material for: Human microphysiological systems of aging recreate the in vivo process expediting evaluation of anti-geronic strategies
Source: Nat Biomed Eng. 2026 Mar 25;10(10):2195–212. doi: 10.1038/s41551-026-01618-6 (PMC13672102; doi:10.1038/s41551-026-01618-6)
Supplement: Supplementary file 3 [file 41551_2026_1618_MOESM3_ESM.pdf]

nature portfolio

Peer Review File

Human microphysiological systems of aging recreate the in vivo process expediting evaluation of anti-gerontic strategies

Corresponding Author: Prof Andreas Stahl

Version 0:

Decision Letter:

Dear Professor Stahl,

Thank you again for submitting to *Nature Biomedical Engineering* your manuscript, "Aging in human microphysiological systems closely recreates the in vivo process with insights on rejuvenation", and please accept my apologies for the time taken in making a decision. The manuscript has been seen by 4 experts, but one of them is yet to provide us with their comments, depsite our chasing efforts. You will find the reports of the other three experts at the end of this message. Should the missing reviewer provide their comment, we will forward it to you, but at this point I find it unlikely.

You will see that the reviewers appreciate aspects of the work. However, they articulate concerns about the degree of support for some of the claims and about the advance that the work represents over relevant published studies, and provide useful suggestions for improvement. We hope that with substantial further effort you can address the criticisms, increase the strength of the evidence, and convince the reviewers of the merits of the study.
In particular, we would expect that a revised version of the manuscript provides:

* Broader analysis of the physiological relevance of the model, as per the many comments of Reviewer #1 and #2.
* Extended analysis of aging mechanisms within the chip.
* Stronger evidence of the applicability of the aging system.
* Clarification of the classification algorithms used throughout the work, as well as threshold used to draw conclusion about aging biomarkers.
* Thorough methodological reporting, with emphasis on facilitating reproducibility.

When you are ready to resubmit your manuscript, please upload the revised files, a point-by-point rebuttal to the comments from all reviewers, the reporting summary, and a cover letter that explains the main improvements included in the revision and responds to any points highlighted in this decision.

Please follow the following recommendations:

* Clearly highlight any amendments to the text and figures to help the reviewers and editors find and understand the changes (yet keep in mind that excessive marking can hinder readability).

* If you and your co-authors disagree with a criticism, provide the arguments to the reviewer (optionally, indicate the relevant points in the cover letter).

* If a criticism or suggestion is not addressed, please indicate so in the rebuttal to the reviewer comments and explain the reason(s).

* Consider including responses to any criticisms raised by more than one reviewer at the beginning of the rebuttal, in a section addressed to all reviewers.

* The rebuttal should include the reviewer comments in point-by-point format (please note that we provide all reviewers will the reports as they appear at the end of this message).

* Provide the rebuttal to the reviewer comments and the cover letter as separate files.

We expect that you will be able to resubmit the manuscript within 25 weeks of receiving this message. If this is the case, you

will be protected against potential scooping. Otherwise, we will be happy to consider a revised manuscript as long as the significance of the work is not compromised by work published elsewhere or accepted for publication at *Nature Biomedical Engineering*.

We hope that you will find the referee reports helpful when revising the work. Please do not hesitate to contact me should you have any questions.

Best wishes,

Valeria

__
Dr Valeria Caprettini
Senior Editor, *Nature Biomedical Engineering*

____
Reviewer #1 (Report for the authors (Required)):

The authors propose a novel microphysiological system (MPS) based on hiPSC technology, which simulates human tissue aging through heterochronic serum perfusion and integrates a machine learning model for age prediction, offering a unique platform for studying aging mechanisms. Under the induction of aged plasma, the authors rapidly established aging phenotypes in adipose and liver tissues (within 4 days) and explored cross-tissue cascading aging effects (adipose-liver). Based on the WAT-MPS aging model, the authors developed a transcriptome-based machine learning model for predicting human WAT age, achieving an accuracy of over 90%. Furthermore, the authors utilized WAT-MPS and liver-MPS for screening anti-aging compounds. The following suggestions may help the authors to improve this manuscript:

1. In Figure 1, prior to conducting experiments with WAT-MPS and iHEP-MPS, the authors should perform functional validation to confirm that the induced cells have successfully differentiated into fully functional adipocytes or hepatocytes. This step is essential to establish the physiological relevance of the model.
2. In Figure 2, the authors did not provide sufficient methodological details regarding the unidirectional perfusion from WAT to liver. Specifically, it remains unclear whether components of aged plasma were removed during perfusion. This information is critical for determining whether the observed aging phenotype in liver-MPS is attributable to OCM-pretreated WAT-MPS or residual aged plasma. It is recommended that the authors provide a detailed description of the experimental procedure and include a control group where WAT-MPS was not pretreated with OCM.
3. In Figure 4, while the authors attempted to elucidate the mechanisms of adipose tissue aging using the plasma-induced WAT-MPS aging model, the depth of mechanistic insights could be enhanced. The authors are encouraged to conduct more in-depth analyses to identify molecular targets underlying plasma-induced WAT-MPS aging and validate these findings through genetic manipulation experiments. This would strengthen the versatility and applicability of the model.
4. In Figure 5, although the authors utilized MPS for screening compounds that delay or reverse aging, the number of aging-related detection markers used was relatively limited. It is recommended to include additional assays, such as SA-β-gal staining and measurements of senescence-associated proteins (e.g., p21, p16), to provide a more comprehensive evaluation of the anti-aging effects.
5. The results suggest that young or old circulatory milieu exhibit dimorphic patterns based on the sex of serum donors. This finding raises important questions about potential sex-specific responses in hiPSC-derived MPSs. Would hiPSC-derived MPSs from male and female donors respond differently to the same experimental conditions? Do these MPSs show sex-dependent variations in receptor expression, signaling pathways, or functional responses? If so, how might these differences contribute to the observed outcomes?
6. The authors used human-on-chip aging models for drug screens, including dasatinib and quercetin (DQ). It would be valuable to determine whether DQ selectively eliminates senescent cells rather than normal cells in hiPSC-derived MPSs using IF staining or FACS analysis.
7. Importantly, while many results focus on the mechanism of heterochronic parabiosis, the key factor in the old circulatory milieu (OCM) that induces MPS aging remains unclear. Identifying this factor is crucial for understanding the underlying mechanisms.
8. Regarding the calculation of transcriptional noise, the authors define genes with log2(CVold/CVyoung) > 0 as transcriptional noise biomarkers. This threshold implies that any gene with marginally higher CVold than CVyoung would be classified as noise-associated, regardless of statistical significance. However, this threshold is overly lenient and lacks scientific justification, potentially leading to substantial false-positive identifications.
9. In the section on the “Transcriptome-based Age Group Prediction Model,” the authors claim to have used the K-Nearest Neighbors (KNN) classification algorithm. However, the code uploaded to GitHub indicates that a random forest algorithm was actually employed. This inconsistency needs to be addressed and clarified.

10. Why did the authors opt for the K-Nearest Neighbors (KNN) or Random Forest algorithms in this study rather than other classification methods such as XGBoost, Multi-Layer Perceptron (MLP), or Support Vector Machine (SVM)? It is advisable that the authors provide detailed justifications for their choice of KNN and Random Forest, including an evaluation of whether alternative algorithms were considered and why they were ultimately not selected.
11. The analysis does not adequately address key covariates such as diet, smoking, and alcohol use, which are extensively

documented in the GTEx metadata. Given the well-established influence of these factors on hepatic and adipose gene expression, their exclusion may introduce significant confounding variables that could affect the validity of age-related conclusions.
12. P16 expression is predominantly nuclear. However, the images presented in Figure 1a of the manuscript suggest that the fluorescence staining for P16 lacks specificity. This raises concerns regarding the reliability of the observed results. Furthermore, it should be noted that the field currently lacks a well-validated, highly specific antibody for P16, which may contribute to the challenges in achieving clear and specific staining.

Reviewer #2 (Report for the authors (Required)):

Qi et al. report the development of a novel microphysiological system (MPS) designed for real-time control of human aging in fat and liver. The study presents intriguing data on transcriptomic profiling and its similarities with human aging, along with an exploration of longevity interventions. While the system appears promising and potentially valuable for studying aging ex vivo, the lack of extensive experimental validation and the absence of functional readouts limit its impact and applicability.

Major Points
• The study suggests that senescence is induced quite early. Notably, p16 induction—typically considered a marker of late senescence—is already prominent just four days after exposure to aged serum. A more comprehensive time course of senescence marker expression would be valuable to better capture the dynamic nature of the senescent state.
• A broader analysis of senescence-associated secretory phenotype (SASP) markers is recommended to provide a more comprehensive understanding of the system’s aging phenotype.
• While the study presents interesting data on gene expression changes in metabolic mediators, this aspect remains insufficiently explored. Further validation, including functional analyses, would strengthen the conclusions.
• Figure 2 provides a snapshot of transcriptomic changes after four days of exposure to aged serum. It is crucial to assess the stability of these changes, particularly given the potential application of this system for pharmacological or genetic interventions aimed at mitigating, preventing, or reversing aging phenotypes.
• While transcriptional changes offer valuable insights, the system should also be characterized using additional omics approaches to provide a more holistic perspective on its aging-related alterations.
• RNA sequencing (RNA-seq) was performed on both fat and liver, but the analysis primarily focuses on fat. Including more liver-specific data and clearly delineating similarities and differences between fat and liver would enhance the study.
• In Figure 5, each tested intervention should be independently validated in a simpler, less complex model to ensure robustness.
• The final section of Figure 5, which demonstrates the partial reversal of aging traits through exposure to young serum, represents a particularly compelling aspect of the study. Expanding this analysis would help justify the use of specific terminology (e.g., "rejuvenation") in the title and introduction.
• One of the most intriguing aspects of the model is its potential to reveal sex-specific differences in aging responses. However, this remains underexplored and should be more thoroughly considered.

Minor Points
• The manuscript would benefit from thorough editing, as some sentences appear fragmented, grammatical errors are present, and references to figures are occasionally unclear.
• In the introduction, it would be beneficial to reference additional well-established interventions known to delay aging, such as caloric restriction, to provide broader context. Currently, the discussion emphasizes heterochronic blood exchange without acknowledging other established strategies.
Please review the sentence in lines 203–205, as it may require clarification or revision.

Reviewer #3 (Report for the authors (Required)):

Manuscript [nBME-25-0200] entitled “Aging in human microphysiological systems closely recreates the in vivo process with insights on rejuvenation” showed that a model indicating the aging transmission mechanism from adipose tissue to hepatocytes based on iPSC. This system can quickly simulate the aging process in vitro, that provided a new perspective for aging mechanisms and rejuvenation strategies. The authors also conducted massive omics analyses and revealed the changes of some aging-related key pathways and genes. However, there are several concerns that may significantly damp the overall quality and suitability for publication in nBME.

1. The authors have detected many biomarkers of aging including P16, SASP, PCK1 and SREBP1c, and evaluated by massive omics analysis. These changes have reported in previous studies (Carlos López-Otín,2023,Cell; Vall-Llaura N,2019, Redox Biol;). In addition, heterochronic parabiosis (Pálovics R,2022,Nature) and MPS (Ao Z, 2022,Adv Sci; Park S,2024, Aging Cell) have also reported in aging field.

2. Why does aging shift from fat aging to liver aging? What is the rationale for experimental design? Although there are some reports show the adipose tissueliver axis (Jamerson LE,2024, Antioxidants), the background information is

unclear.

3.There are some mistakes in the manuscript. For example, the number of key words in this manuscript is 13. Fig 1B showed the concentration is negative value.

Version 1:

Decision Letter:

Dear Prof Stahl,

Thank you again for submitting to *Nature Biomedical Engineering* your revised manuscript "Aging in human microphysiological systems closely recreates the in vivo process expediting evaluation of anti-gerontic strategies". The manuscript has been seen by the original 3 experts, whose reports you will find at the end of this message. You will see that two of the reviewers are happy wit hyour responses, and only Reviewer #3 raises a few remaining criticisms.
We hope that you can provide us with a revised version of the manuscript where the limitations of the induced aging phenotypes using serum from elderly donors are discussed, as well as the potential future work that incorporates a real-time aging monitoring indicator.

As before, when you are ready to resubmit your manuscript, please upload the revised files, a point-by-point rebuttal to the comments from all reviewers, and a cover letter that explains the main improvements included in the revision and responds to any points highlighted in this decision.

As a reminder, please follow the following recommendations:

* Please submit your revised manuscript in a word doc or LaTeX format.

* Clearly highlight any amendments to the text and figures to help the reviewers and editors find and understand the changes (yet keep in mind that excessive marking can hinder readability).

* If you and your co-authors disagree with a criticism, provide the arguments to the reviewer (optionally, indicate the relevant points in the cover letter).

* If a criticism or suggestion is not addressed, please indicate so in the rebuttal to the reviewer comments and explain the reason(s).

* Consider including responses to any criticisms raised by more than one reviewer at the beginning of the rebuttal, in a section addressed to all reviewers.

* The rebuttal should include the reviewer comments in point-by-point format (please note that we provide all reviewers will the reports as they appear at the end of this message).

* Provide the rebuttal to the reviewer comments and the cover letter as separate files.

We expect that you will be able to resubmit the manuscript within <u>8 weeks</u> of receiving message. If this is the case, you will be protected against potential scooping. Otherwise, we will be happy to consider the next version of the manuscript as long as the advance of the work is not compromised by work published elsewhere or accepted for publication at *Nature Biomedical Engineering*.

We look forward to receive a further revised version of the work. Please do not hesitate to contact me should you have any questions.

Best wishes,

Valeria

__
Dr Valeria Caprettini
Senior Editor, *Nature Biomedical Engineering*

____
Reviewer #1 (Report for the authors (Required)):

In this revised manuscript, the authors have addressed the major concerns of reviewers. It is suitable for publication on Nature Biomedical Engineering.

Reviewer #2 (Report for the authors (Required)):

The authors have done an excellent job in revising the manuscript and have fully addressed the concerns I raised in my initial review. The revisions are thorough, clearly explained, and strengthen the overall impact of the study. I sincerely thank the authors for the time, effort, and dedication they have invested in improving the manuscript, which was already of high quality.

Reviewer #3 (Report for the authors (Required)):

After careful review, we believe that the research is not innovative enough to be published in Nature BME. The major concern lies in the limited functionality and translational relevance of the microphysiological system (MPS) presented. While the system demonstrates the capability to culture and interconnect different tissue types, it lacks robust and validated functionality for detecting and analyzing key hallmarks of aging. The claim that aging phenotypes—including senescence, oxidative damage, and metabolic dysfunction—can be reliably induced within just 4 days using serum from elderly donors appears physiologically overstated and is not sufficiently supported by the data. Such rapid induction does not adequately recapture the complex, multifactorial, and gradual process of human aging, thereby limiting the system's utility for mechanistic studies of aging or for screening anti-aging therapeutics. Furthermore, the MPS does not represent a substantial technological advance over existing platforms due to the fact that there are many MPS devices available, and this study did not integrate the real-time aging monitoring indicator platform into the MPS device. The manuscript fails to introduce transformative innovations in design, readout capabilities, or physiological fidelity.

Version 2:

Decision Letter:

Dear Dr. Stahl,

Thank you for submitting your revised manuscript "Aging in human microphysiological systems closely recreates the in vivo process expediting evaluation of anti-gerontic strategies" (NBME-25-0200B). Having assessed the added discussion on the limitation of serum-induced aging, I am pleased to write that we shall be happy to publish the manuscript in Nature Biomedical Engineering, pending compliance with our editorial and formatting guidelines.

We are now performing detailed checks on your paper and will send you a checklist detailing our editorial and formatting requirements. Please do not upload the final materials and make any revisions until you receive this additional information from us.

TRANSPARENT PEER REVIEW
Nature Biomedical Engineering offers a transparent peer review option for new original research manuscripts submitted from 17th February 2021. We encourage increased transparency in peer review by publishing the reviewer comments, author rebuttal letters and editorial decision letters if the authors agree. Such peer review material is made available as a supplementary peer review file. **Please state in the cover letter 'I wish to participate in transparent peer review' if you want to opt in, or 'I do not wish to participate in transparent peer review' if you don't.** Failure to state your preference will result in delays in accepting your manuscript for publication.

Please note: we allow redactions to authors' rebuttal and reviewer comments in the interest of confidentiality. If you are concerned about the release of confidential data, please let us know specifically what information you would like to have removed. Please note that we cannot incorporate redactions for any other reasons. Reviewer names will be published in the peer review files if the reviewer signed the comments to authors, or if reviewers explicitly agree to release their name. For more information, please refer to our FAQ page.

ORCID
IMPORTANT: Non-corresponding authors do not have to link their ORCIDs but are encouraged to do so. Please note that it will not be possible to add/modify ORCIDs at proof. Thus, please let your co-authors know that if they wish to have their ORCID added to the paper they must follow the procedure described in the following link prior to acceptance: https://www.springernature.com/gp/researchers/orcid/orcid-for-nature-research

Author names using non-Roman characters
Nature Portfolio journals can support presentation of author names using non-Roman characters in the HTML version of the

article. If you wish to, please include author names in parentheses after the Roman-character spelling; see example online here. Currently supported scripts are: Arabic, Chinese, Cyrillic, Devanagari, Greek, Hebrew, Hangul, Japanese and Persian. You will be asked to verify the rendering is correct at proof stage.

Sincerely,

Valeria

__
Dr Valeria Caprettini
Senior Editor, *Nature Biomedical Engineering*

Version 3:

Decision Letter:

Dear Prof Stahl,

I am happy to inform you that your manuscript, "Human microphysiological systems of aging recreate the in vivo process expediting evaluation of anti-gerontic strategies", has now been accepted for publication in *Nature Biomedical Engineering*.

Over the next few weeks, the figures will be checked for production quality, the text edited to ensure that it conforms to house style, and the manuscript typeset.

Our Articles are published about 40 days after the acceptance date (we recommend that you inform your institutional press office of this timeframe), and you will be notified of the actual publication date a few days in advance. Articles can be published any working day of the week, and are pushed live shortly after 10 am London time.

Publishing agreement. You will be asked to digitally sign a publishing agreement (grant of rights). After the signed publishing agreement has been received, the proofs of the article will be sent to you for review. If you have any queries during the production process, or you cannot meet the requested deadline for returning the proofs, please contact rjsproduction@springernature.com.

Nature Biomedical Engineering is a Transformative Journal. Authors may publish their research with us through the traditional subscription access route, or make their paper immediately open access through payment of an article-processing charge. More information about publication options is available.

You may need to take specific actions to comply with funder and institutional open-access mandates. If the work described in the accepted manuscript is supported by a funder that requires immediate open access (as outlined, for example, by Plan S) and your manuscript was originally submitted on or after January 1st 2021, then you should select the gold OA route. Authors selecting subscription publication will need to accept our standard licensing terms (including our self-archiving policies), and these will supersede any other terms that the author or any third party may assert apply to any version of the manuscript.

Acceptance of your manuscript is conditional on agreement, by all authors, with both our media embargo and confidentiality and pre-publicity policies. In particular, you may arrange your own publicity of the Article (for instance, through your institutional press office), as long as you ensure that journalists strictly adhere to the media embargo.

To assist you in disseminating the work, as soon as the Article is published you will be able to take advantage of the Springer Nature SharedIt initiative to generate a unique shareable link to the Article that will allow anyone (with or without a subscription) to read it. Recipients of the link who are subscribers will also be able to download and print the PDF.

Thank you for having submitted this work to *Nature Biomedical Engineering*.

Best wishes,

Valeria

—
Dr Valeria Caprettini
Senior Editor, *Nature Biomedical Engineering*

Open Access This Peer Review File is licensed under a Creative Commons Attribution 4.0 International License, which permits use, sharing, adaptation, distribution and reproduction in any medium or format, as long as you give appropriate credit to the original author(s) and the source, provide a link to the Creative Commons license, and indicate if changes were made.
In cases where reviewers are anonymous, credit should be given to 'Anonymous Referee' and the source.
The images or other third party material in this Peer Review File are included in the article's Creative Commons license, unless indicated otherwise in a credit line to the material. If material is not included in the article's Creative Commons license and your intended use is not permitted by statutory regulation or exceeds the permitted use, you will need to obtain permission directly from the copyright holder.
To view a copy of this license, visit https://creativecommons.org/licenses/by/4.0/

Point-by-point response to reviewers' comments

Reviewer #1 (Report for the authors (Required)):

1. In Figure 1, prior to conducting experiments with WAT-MPS and iHEP-MPS, the authors should perform functional validation to confirm that the induced cells have successfully differentiated into fully functional adipocytes or hepatocytes. This step is essential to establish the physiological relevance of the model.

Thank you for the comment. Both iPSC derived adipocytes and hepatocytes were fully characterized based on gene expression and function in MPS in prior publications (Qi. et. al Small 2022, Groeger et. al. Nat. Comm. 2023, Qi. et. al Nat. Comm. 2024). We now cite these publications more clearly and provide a graphical overview in Fig.1.

2. In Figure 2, the authors did not provide sufficient methodological details regarding the unidirectional perfusion from WAT to liver. Specifically, it remains unclear whether components of aged plasma were removed during perfusion. This information is critical for determining whether the observed aging phenotype in liver-MPS is attributable to OCM-pretreated WAT-MPS or residual aged plasma. It is recommended that the authors provide a detailed description of the experimental procedure and include a control group where WAT-MPS was not pretreated with OCM.

We used the same liver-MPS arrangement as in our previous publication (Qi. et al. 2024 Nat. Comm.). We now provide more details in the methods section (line 763-773) and provide a graphical overview in Fig.2.

We appreciate your concern regarding residual aged plasma. Similar questions were asked in our previous papers (2023, Small; 2024 Nat. Comm.), and we have experimentally addressed the carry-over of pre-treatments such as lipopolysaccharide, a very effective and concern-able endotoxin, and found it to be negligible. This is because the microfluidic platform only contains sub-microliter volumes of medium that can quickly (in few seconds) diffuse to and be easily flushed out by the rinsing with medium between two treatments and the initial treatment with 5% serum is highly diluted by the perfusion medium (i.e. <<1 uL residual liquid diluted in >1 mL medium).

3. In Figure 4, while the authors attempted to elucidate the mechanisms of adipose tissue aging using the plasma-induced WAT-MPS aging model, the depth of mechanistic insights could be enhanced. The authors are encouraged to conduct more in-depth analyses to identify molecular targets underlying plasma-induced WAT-MPS aging and validate these findings through genetic manipulation experiments. This would strengthen the versatility and applicability of the model.

We appreciate the reviewer's suggestion and have accordingly conducted additional experiments on genetic mechanisms to further identify key targets. Among the 11 aging-related markers analyzed in Fig.4, CDKN2A (p16) emerged as a central regulatory node, exhibiting strong correlations with aging, and functionally connecting the remaining 10 markers through upstream and/or downstream signaling pathways. Thus, knockdown of CDKN2A is anticipated to directly suppress cell cycle arrest associated with senescence and diminish the autocrine pro-aging effects of SASPs, such as IL-6 and IL-8.[1]

To validate its functional role, we performed siRNA-mediated knockdown of CDKN2A together with and after OCM treatment to assess prevention and reversal of aging hallmarks. In both cases, CDKN2A knockdown led to a remarkable reduction in aging phenotypes and improved metabolic profiles, with greater efficacy observed when knockdown was performed after removal of OCM. These findings are consistent with our drug screening results, which demonstrated that removal of OCM enhances treatment efficacy.

Collectively, this experiment highlights p16 as a pivotal target for anti-aging interventions and demonstrates the broader utility of our model—not only for drug screening but also for genetic perturbation studies. These new data have been incorporated into the revised Fig. 6.

Further, additional discussion of molecular targets of OCM were included in the new data on liver aging shown in response to reviewer#2 Q6 and manuscript discussion (line 383-409).

4. In Figure 5, although the authors utilized MPS for screening compounds that delay or reverse aging, the number of aging-related detection markers used was relatively limited. It is recommended to include additional assays, such as SA-β-gal staining and measurements of senescence-associated proteins (e.g., p21, p16), to provide a more comprehensive evaluation of the anti-aging effects.

We agree and performed SA-b-gal staining and DNA oxidative damage and P16 ELISA assays with siRNA P16 , heterochronic microfluidics, and the most effective pharmacological setting (dilution + drugs) as shown below and in the new Fig. 6c (or previous Fig.5c).

5. The results suggest that young or old circulatory milieu exhibit dimorphic patterns based on the sex of serum donors. This finding raises important questions about potential sex-specific responses in hiPSC-derived MPSs. Would hiPSC-derived MPSs from male and female donors respond differently to the same experimental conditions? Do these MPSs show sex-dependent variations in receptor expression, signaling pathways, or functional responses? If so, how might these differences contribute to the observed outcomes?

This is an interesting question. Even when using an XY hiPSC line, we can clearly demonstrate the development of sexually dimorphic responses driven by the biological sex of the serum donor. So, effects of sex of the iPSC donors appears to be secondary but could have complementary effects. We now add this notion to our discussion (line 340) but refrain from experimental verification as methods for the induction of sexually dimorphic gene expression in iPSC derived adipocytes and hepatocytes have not yet been reported and are likely to involve more than sex chromosomes requiring the establishment of pulsatile hormonal milieus that would be beyond the scope of this manuscript.

6. The authors used human-on-chip aging models for drug screens, including dasatinib and quercetin (DQ). It would be valuable to determine whether DQ selectively eliminates senescent cells rather than normal cells in hiPSC-derived MPSs using IF staining or FACS analysis.

In response to the reviewer's concern, we validated the senolytic activity of DQ in our MPS model by performing senescence-associated staining. The results confirmed a reduction in senescent cells and have been included in the revised Fig. 6 of the manuscript.

7. Importantly, while many results focus on the mechanism of heterochronic parabiosis, the key factor in the old circulatory milieu (OCM) that induces MPS aging remains unclear. Identifying this factor is crucial for understanding the underlying mechanisms.

Heterochronically resetting the composition of systemic milieu to either pro-gerontic or to healthier and younger states involves more than one factor ([2-7],and many publications). The organ-aging-on-a-chip platform that was established in our work enables the future detailed studies on these complex mechanisms. The above is now added to the Discussion. Further, we discuss the key candidate pro-gerontic systemic factors and anti-aging strategies:

Starting from Line 383: " Our system showed that inflammatory cytokines and growth factors, the two major components of SASPs[8,9], were primary drivers of aging, but their roles differed between WAT and liver: OCM-induced liver aging showed a stronger dependence on growth factors (*BDNF, GDF15, NRG*) rather than inflammatory cytokine (*CCL2*). This was accompanied by activation of the growth-factor-responsive cell cycle regulator *CDKN2B* (p15) via the TGF-β/SMAD pathway, and induction of an inflammation suppressor, *TNFAIP3* (A20) to inhibit the NF-κB pathway. In contrast, WAT aging showed a clear inflammatory propensity, characterized by an aging loop involving immediate-early transcription factors (*JUN, FOS, EGR1*), the senescence center *CDKN2A* (p16), and key inflammatory triggers, modulators, and SASPs (*PTGS2, IL6, IL8*) that reinforce autocrine senescence and propagate paracrine aging signals.

TGF-β, a growth factor and SASP component that is elevated during aging[9], may serve as a central regulator distinguishing WAT from liver aging, as it suppresses adipogenesis[10] while promoting hepatogenesis[11,12]. This role is strongly supported by our findings: in WAT-MPS, YCM and OCM induced divergent genotypic and phenotypic outcomes, whereas in liver-MPS, both conditions consistently converged toward enhanced developmental and hepatogenic programs.

Interestingly, OCM-pretreated WAT caused liver aging with similar features to that of OCM-induced aging and both processes closely resembled in-vivo aging (overlapped GO terms and aging biomarkers of expression and biological noise). Growth factors (*BDNF*, *GDF15*) and aging-related regulatory pathways (*CDKN2B*) as well as inflammation suppressor (*TNFAIP3*) were upregulated in the liver exposed to OCM-aged WAT. However, inflammaging signatures (*EGR1*, *PTGS2*, *IL6*), proinflammatory cytokines/SASPs (*CCL20*, *CXCL2*) were also elevated. Based on the secretome analyses of OCM vs. YCM-treated-WAT (Fig.2g,h and Extended Data Fig. 8a) and TNF□ ELISA (Fig.1b), the heterochronic interorgan signatures were due to SASPs secreted from the OCM-aged WAT and hepatogenic growth factors secreted from the YCM-exposed youthful WAT (BMP4[13], FGFs[14])."

And anti-aging targeting starting at line 422: "While not exhaustive, we tested multiple anti-gerontic strategies to demonstrate the value of our aging-on-a-chip system for discovery applications focusing on the adipocyte-MPS. Serum dilution, though a simple approach, proved highly effective in removing SASPs, thereby attenuating aging phenotypes and enhancing the efficacy of subsequent interventions. This finding aligns with the growing clinical interest in therapeutic plasma exchange (TPE) as an emerging rejuvenation modality[15]. Oxytocin treatment demonstrated some of the more promising anti-gerontic effects by simultaneously promoting lipolysis, reducing inflammation, and correcting visceral fat redistribution[16-18]. Inhibition of TGF-β signaling through its receptor subunit ALK5 also produced robust anti-gerontic effects, potentially due to its capacity to both

promote adipogenesis and alleviate TGF-β/SMAD3-driven senescence. However, ALK5i treatment also resulted in the expansion of aged adipose progenitor populations based on increased LIFR in Fig.6c.”

8. Regarding the calculation of transcriptional noise, the authors define genes with log2(CVold/CVyoung) > 0 as transcriptional noise biomarkers. This threshold implies that any gene with marginally higher CVold than CVyoung would be classified as noise-associated, regardless of statistical significance. However, this threshold is overly lenient and lacks scientific justification, potentially leading to substantial false-positive identifications.
Clarify in the method and figure legend

This is a great point, which allows us to clarigy that first, since we utilized Levene’s test $p<0.05$ as the threshold, all 37 markers are ensured to be statistically significant noises. We utilized the log2 (CVold/CVyoung) > 0 as the other threshold because the highest noise of the overlapped genes in GTEx database is about 1.7-fold or log2(CVold/CVyoung) = 0.766, even below the common threshold of log2 (CVold/CVyoung) > 1, i.e. 2-fold.

We understand and agree to the reviewer’s concern about the low threshold of log2(CVold/CVyoung) > 0. To address this, we further gated the 37 noise markers to log2(CVold/CVyoung) > 0.5, resulting in the top 5 genes. We also reduced the number of predicted gene patterners down to only 5 genes. Interestingly, these analyses still yielded similar core PPI network and enriched functions mainly in insulin and mTOR associated signaling pathways as well. Therefore, thanks to the reviewer’s comment, we were able to show that our claim and conclusion are valid with higher gating threshold.

Considering the PPI from 37 biomarkers still provided some insightful pathways, such as those related to mitochondria and interleukin functions, we kept the original 37 biomarker network, and labeled the further gated core PPI network in the Fig.4d. The summary table of our thresholding results, in the measures of log2(CVold/CVyoung) and Levene’s test p values, is provided in the supplementary information. Above rationale is involved in the Method section.

**Levene’s p values**		< 0.01	< 0.05
**Log2(CVold /CYyoung)**	>0	1	37
	>0.05	0	28
	>0.1	0	26
	>0.15	0	20
	>0.2	0	20
	>0.25	0	18
	>0.5	0	5
	>0.75	0	1
	>1	0	0

9. In the section on the “Transcriptome-based Age Group Prediction Model,” the authors claim to have used the K-Nearest Neighbors (KNN) classification algorithm. However, the code uploaded to GitHub indicates that a random forest algorithm was actually employed. This inconsistency needs to be addressed and clarified.

In the initial version of the classification model, K-nearest neighbors (KNN) was used as the core algorithm. In the final version, which was used to generate the figures and uploaded to GitHub, the classification function was updated to include three algorithm options: Random Forest, KNN, and Support Vector Machine (SVM). The model achieving the best performance during cross-validation on the GTEx dataset with the Random Forest that was selected for downstream prediction of MPS ages.

We are sorry for this delayed updating and greatly appreciate the reviewer reminded us. The Methods section is updated to clarify this change and to indicate that the models shown are based on Random Forest (line 1031-1037).

10. Why did the authors opt for the K-Nearest Neighbors (KNN) or Random Forest algorithms in this study rather than other classification methods such as XGBoost, Multi-Layer Perceptron (MLP), or Support Vector Machine (SVM)? It is advisable that the authors provide detailed justifications for their choice of KNN and Random Forest, including an evaluation of whether alternative algorithms were considered and why they were ultimately not selected.

As noted in the previous question, our classification fitting function includes K-nearest neighbors (KNN), Random Forest, and Support Vector Machine (SVM), with the final model selected based on cross-validation accuracy. During the prototyping stage,

we also tested a multilayer perceptron (MLP), but it exhibited signs of overfitting and performed poorly in cross-validation. Similarly, XGBoost was explored during initial testing but also showed overfitting under the tested hyperparameter settings.

We have provided above rationale of method selection in the revised manuscript (line 1031-1037).

11. The analysis does not adequately address key covariates such as diet, smoking, and alcohol use, which are extensively documented in the GTEx metadata. Given the well-established influence of these factors on hepatic and adipose gene expression, their exclusion may introduce significant confounding variables that could affect the validity of age-related conclusions.

The new paradigm that our data shows is that aging dominates over such covariates when studying the systemically induced health decline of fat and liver on-chip, which is very interesting and is now better discussed in Results and Discussion.

While nuances of the health-styles of the blood donors would be interesting to study, a rigorous investigation into these variables would require a substantially larger and purpose-built cohort and a new separate manuscript with many figures[23].

Although it is theoretically feasible to investigate such lifestyle risk factors using our machine learning–based aging fat model, doing so would require substantial bioinformatic and computational resources, an effort significant enough to establish a separate dedicated study rather than in the current one. For example, a recently published study addressed the similar question using a large-scale dataset of over 40,000 individuals from the UK Biobank (Hamilton Se-Hwee Oh et al., Nature Medicine, 2025[24]), highlighting the scale and complexity involved in such analyses. Besides, the publicly available metadata from the GTEx database is limited to basic donor information (e.g., age, sex, and date of birth). More detailed variables, such as diet, smoking, or alcohol use, are not included in the downloadable datasets and require special access through a formal approval process bouncing between NIH and GTEx administrators. We have initiated this process, but given the current revision timeline, approval is unlikely to be granted in time (see attached screenshot for our initial request dated one year ago but still pending).

We appreciate the reviewer's insightful suggestion, which highlights a compelling direction for future cohort-based investigations. In recognition of its value, we have added a brief discussion of this point in the revised manuscript (line 453-458)

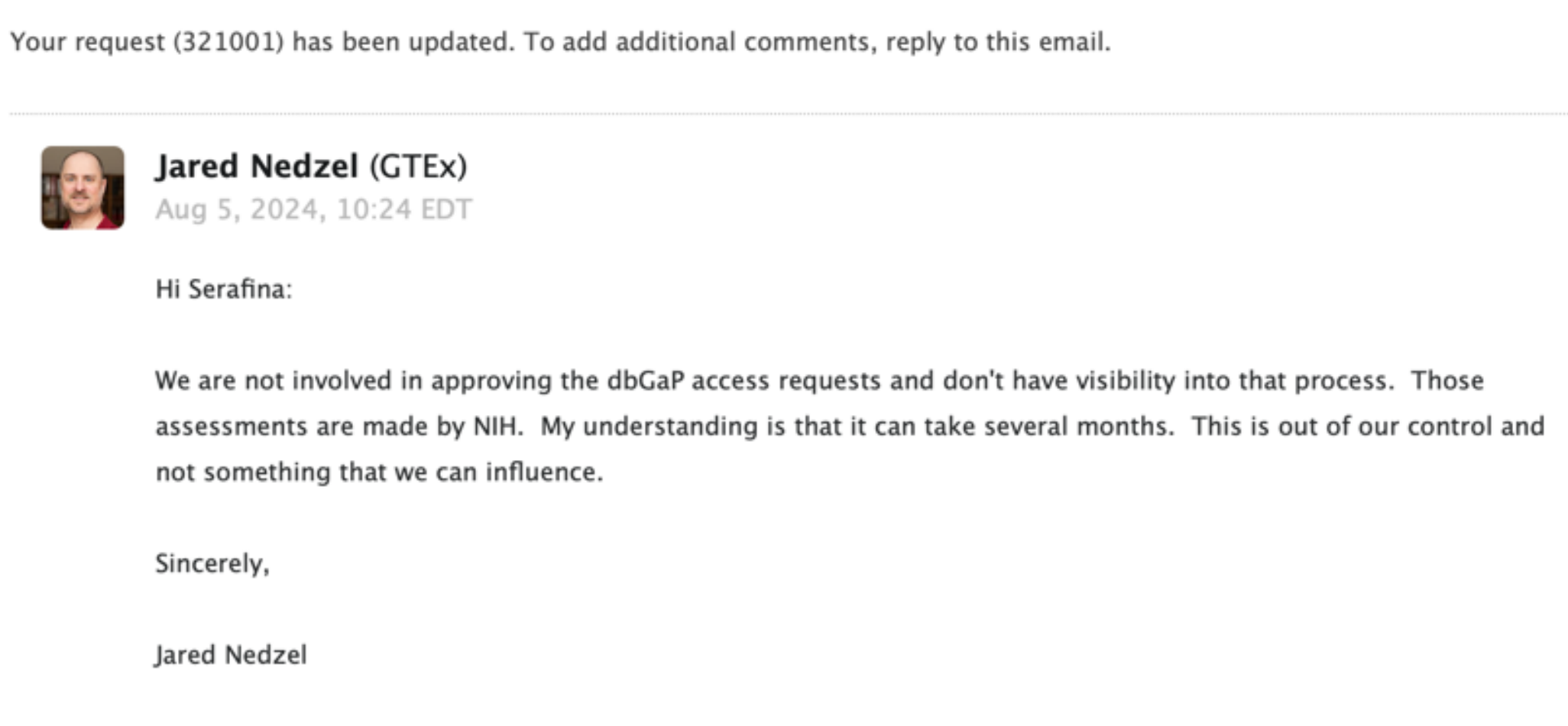
Your request (321001) has been updated. To add additional comments, reply to this email.

Jared Nedzel (GTEx)
Aug 5, 2024, 10:24 EDT

Hi Serafina:

We are not involved in approving the dbGaP access requests and don't have visibility into that process. Those assessments are made by NIH. My understanding is that it can take several months. This is out of our control and not something that we can influence.

Sincerely,

Jared Nedzel

12. P16 expression is predominantly nuclear. However, the images presented in Figure 1a of the manuscript suggest that the fluorescence staining for P16 lacks specificity. This raises concerns regarding the reliability of the observed results. Furthermore, it should be noted that the field currently lacks a well-validated, highly specific antibody for P16, which may contribute to the challenges in achieving clear and specific staining.

We thank the reviewer for raising the important issue regarding the specificity of commercially available p16 antibodies. This is a legitimate concern and may indeed contribute to variability in immunofluorescence results. As you mentioned, cytoplasmic p16 immunostaining can sometimes be a false-positive artifact due to non-specific antibody binding, as previously reported[25]. Nevertheless, there is also literature suggesting that cytoplasmic localization of p16 may have biological significance in certain aging contexts[26].

To minimize potential false-positive interpretations, we implemented several measures. First, all immunofluorescence images were quantified relative to an IgG negative control to ensure specificity. Second, we confined our analysis to nuclear-localized p16 signals, aligning with the canonical function of p16 as a nuclear CDK4/6 inhibitor. Third, we independently validated p16

upregulation using an ELISA-based assay. These three complementary approaches consistently supported the conclusion that p16 expression increases under aged circulatory conditions.
In addition, we updated the manuscript figures to highlight regions with strong nuclear colocalization of p16 and DAPI. To further address the antibody specificity concern, we also tested an alternative p16 primary antibody (ab108349), which yielded similar staining patterns and results.

We fully understand your and another reviewer's concern about the robustness of aging induction in our model. That is why we incorporated a multi-parametric approach to assess aging phenotypes, including hallmark gene expression, β-galactosidase staining, DNA oxidation, inflammation/SASP profiles, and transcriptomic comparisons with human aging tissues. Our qPCR, transcriptomics, ELISA all confirmed the significant upregulation of CDKN2A (p16) under aged conditions, as one of 11 aging biomarkers. CDKN2A knockdown by siRNA further validated its presence and critical role in aging. These convergent lines of evidence strengthen the reliability of our findings, even considering potential limitations in antibody specificity.

We have updated the p16 immunostaining images and quantification accordingly in the revised manuscript in Fig.1.

Lastly, our new data with siRNA to p16 (Figure 6d,e) confirms the role of this gene/protein in the phenomenon of on-chip induced human tissue aging.

Reviewer #2 (Report for the authors (Required)):

Qi et al. report the development of a novel microphysiological system (MPS) designed for real-time control of human aging in fat and liver. The study presents intriguing data on transcriptomic profiling and its similarities with human aging, along with an exploration of longevity interventions. While the system appears promising and potentially valuable for studying aging ex vivo, the lack of extensive experimental validation and the absence of functional readouts limit its impact and applicability.

Major Points
1• The study suggests that senescence is induced quite early. Notably, p16 induction—typically considered a marker of late senescence—is already prominent just four days after exposure to aged serum. A more comprehensive time course of senescence marker expression would be valuable to better capture the dynamic nature of the senescent state.

We appreciate that the reviewer noticed the fast senescent process, which is exactly what we want to report here. This is a signature advantage of heterochronic blood exchange in vivo, or serum treatment in vitro in our previous study, showing 2-day period is enough to induce significant p16 difference in vivo, or 3~6 days in vitro[6].

We understand the reviewer's concern regarding the absence of direct evidence in this newly developed WAT-MPS, which to our knowledge has not been previously tested in this context. To address this, we conducted a time-course experiment of aging induction over 0 to 4 days, demonstrating that senescent cells begin to stably appear as early as day 2 in our MPS model. The corresponding data have been included in the Supplementary Information.

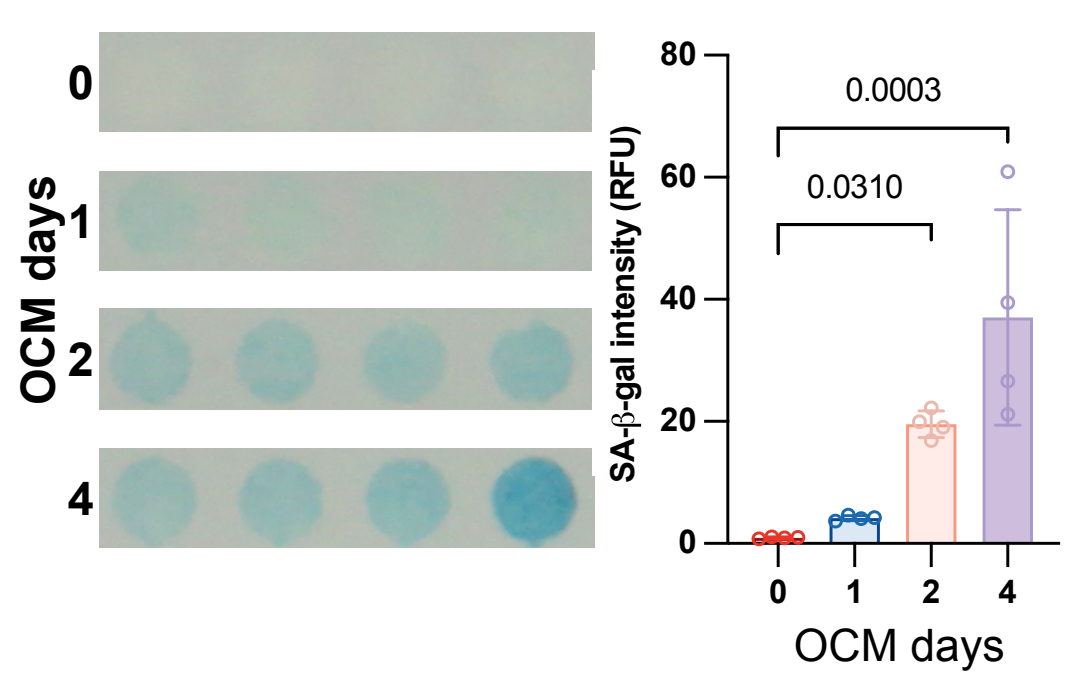

2• A broader analysis of senescence-associated secretory phenotype (SASP) markers is recommended to provide a more comprehensive understanding of the system's aging phenotype.

In response to this comment, we extracted SASP markers from transcriptomic datasets across all measured conditions in this study and summarized them into a comparative table. Overall, 8-day OCM treatment induced the strongest and most widespread upregulation of SASPs. Consistent with other analyses, OCM-treated WAT aging exhibited greater upregulation of inflammatory and immunoregulatory cytokines (TNFSFs, CCLs, CXCLs) but much less in tissue remodeling and development regulators (MMPs and TGFBs) compared to liver. Interestingly, OCM-treated WAT and liver displayed opposite regulatory patterns within

the Serpin superfamily, suggesting distinct roles of these factors in modulating inflammation, metabolism, and cell signaling across tissues. Moreover, aged-WAT-induced liver aging displayed a composite signature that integrated features of both WAT and liver OCM aging, such as increased expression of CCLs and CXCLs consistent with WAT, alongside downregulation of selected Serpin family members consistent with liver.
Notably, the table also exhibited sex-biased aging phenotype with males showing stronger and higher expression of inflammatory markers than females, particularly in WAT, consistent with the well-documented observation that males generally experience faster aging and shorter lifespans than females.

The SASP list is updated as Extended Data 8a, and its implication is discussed in the revised manuscript (line 353,407, 444).

3• While the study presents interesting data on gene expression changes in metabolic mediators, this aspect remains insufficiently explored. Further validation, including functional analyses, would strengthen the conclusions.

We agree with the reviewer that this is important and now included functional analyses of glucose and lipid metabolism for all tests in our original submission (Fig.1-3, 5). Specifically, adipocytes were characterized using insulin-regulated glucose and fatty acid uptake while hepatocytes were phenotyped using fatty acid uptake and glucose production.

4• Figure 2 provides a snapshot of transcriptomic changes after four days of exposure to aged serum. It is crucial to assess the stability of these changes, particularly given the potential application of this system for pharmacological or genetic interventions aimed at mitigating, preventing, or reversing aging phenotypes.

We agree and the results were confirmed to be robust: they were generated from multiple donors of varied ages and different sexes. Although some donor-to-donor variances was detected, transcriptomic analysis still reliably separate young vs. old groups in PCA and provided an extensive list of significantly differentially expressed genes (DEG list). In summary, the changes are reproducible.

We also now directly address the stability of the aging phenotype. Comparing the DEG lists of 8-day OCM (male) to 4-day OCM (male) old we found 188 overlapping genes indicating a that many of the OCM induced changes are stable. Enriched terms for 8 days OCM are very similar to the 4-day old conditions, with the exact same pathways in RIG-I, NOD-like, Cytosolic DNA, Toll-like, TNF, Cytokine-cytokine receptor. Comparison of their respective GO terms yields similar outcomes. Therefore, the dominated signaling pathways are stable in continuous OCM treatment. Our SASP summary table shown in above question also supports the claim of stable aging, as most SASPs upregulated at the 4-day condition were further elevated in the 8-day condition (Extended Data Fig.8a).

Moreover, OCM-induced aging is even too stable to be easily reversed, as demonstrated in Fig. 6 by the low rejuvenation efficiency once OCM was present. This stability further manifested as a form of cellular aging memory after OCM removal, evident in both heterochronic inter-organ aging (aged fat to liver) and heterochronic microfluidics (OO vs. OY).

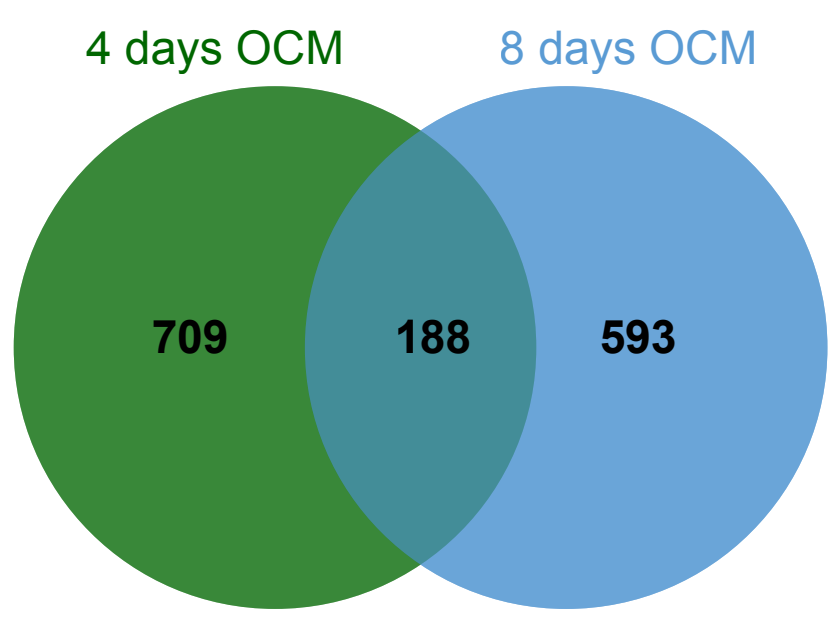

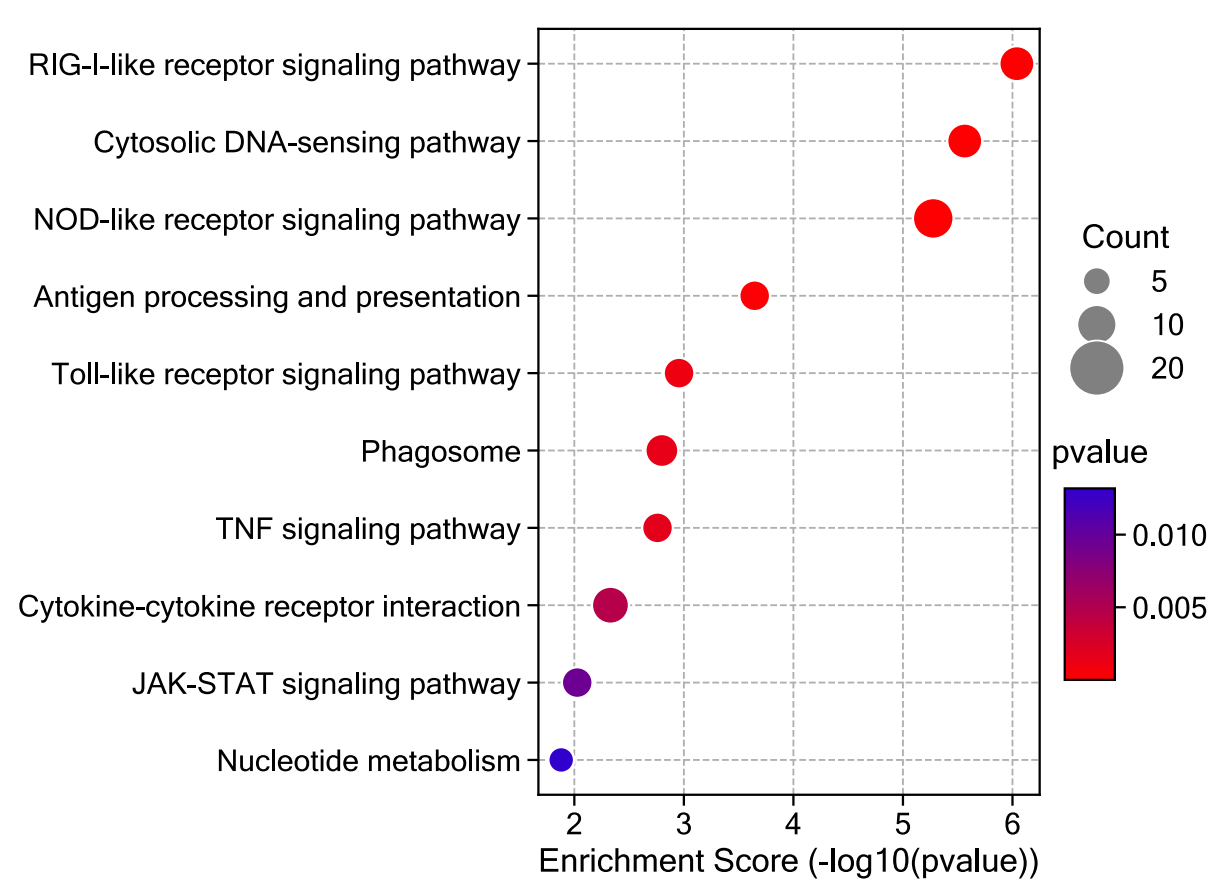

5• While transcriptional changes offer valuable insights, the system should also be characterized using additional omics approaches to provide a more holistic perspective on its aging-related alterations.

We took multiple steps to expand on our transcriptomic analysis. These include functional metabolic assays in all aging conditions, and proteomic profiling of de novo synthesized proteins using bioorthogonal non-canonical amino acid tagging

(BONCAT). We generated a methionyl-tRNA synthetase (MetRS^{L274G}) hiPSC line, which enables the efficient incorporation of azidonorleucine (ANL) into newly synthesized proteins that are traceable after click chemistry on azide-site (Fig.2f). This approach revealed that under the OCM condition, cytokine interactions predominantly occurred in the extracellular space, activating the inflammatory signaling pathway. In contrast, the YCM condition was associated with growth factor signaling that promoted cell proliferation, development, and other fundamental biological processes (Fig.2g,h).

Moreover, the BONCAT analysis uncovered sexually dimorphic proteomic patterns: male samples showed enrichment in developmental processes, whereas female samples exhibited a stronger inflammatory signature. These proteomic findings were consistently supported by the corresponding transcriptomic data. These BONCAT data are now shown in the new Extended Data Fig. 8 b,c.

In addition, we have expanded our study to include a more comprehensive transcriptomic analysis focused on liver aging under OCM exposure and aged fat-derived conditions, now in the new Fig.5

6 • RNA sequencing (RNA-seq) was performed on both fat and liver, but the analysis primarily focuses on fat. Including more liver-specific data and clearly delineating similarities and differences between fat and liver would enhance the study.

We have expanded our study to include a more comprehensive transcriptomic analysis focused on liver aging under OCM exposure and aged fat-derived conditions. We also developed liver aging prediction model following the same algorithm in WAT-MPS.

The difference between fat and liver specific aging mechanism are discussed below and in the revised manuscript:

<u>Starting from Line 383:</u> " Our system showed that inflammatory cytokines and growth factors, the two major components of SASPs[8,9], were primary drivers of aging, but their roles differed between WAT and liver: OCM-induced liver aging showed a stronger dependence on growth factors (*BDNF, GDF15, NRG*) rather than inflammatory cytokine (*CCL2*). This was accompanied by activation of the growth-factor-responsive cell cycle regulator *CDKN2B* (p15) via the TGF-β/SMAD pathway, and induction of an inflammation suppressor, *TNFAIP3* (A20) to inhibit the NF-κB pathway. In contrast, WAT aging showed a clear inflammatory propensity, characterized by an aging loop involving immediate-early transcription factors (*JUN, FOS, EGR1*), the senescence center *CDKN2A* (p16), and key inflammatory triggers, modulators, and SASPs (*PTGS2, IL6, IL8*) that reinforce autocrine senescence and propagate paracrine aging signals.

TGF-β, a growth factor and SASP component that is elevated during aging[9], may serve as a central regulator distinguishing WAT from liver aging, as it suppresses adipogenesis[10] while promoting hepatogenesis[11,12]. This role is strongly supported by our findings: in WAT-MPS, YCM and OCM induced divergent genotypic and phenotypic outcomes, whereas in liver-MPS, both conditions consistently converged toward enhanced developmental and hepatogenic programs.

Interestingly, OCM-pretreated WAT caused liver aging with similar features to that of OCM-induced aging and both processes closely resembled in-vivo aging (overlapped GO terms and aging biomarkers of expression and biological noise). Growth factors (*BDNF*, *GDF15*) and aging-related regulatory pathways (*CDKN2B*) as well as inflammation suppressor (*TNFAIP3*) were upregulated in the liver exposed to OCM-aged WAT. However, inflammaging signatures (*EGR1*, *PTGS2*, *IL6*), proinflammatory cytokines/SASPs (*CCL20*, *CXCL2*) were also elevated. Based on the secretome analyses of OCM vs. YCM-treated-WAT (Fig.2g,h and Extended Data Fig. 8a) and TNF□ ELISA (Fig.1b), the heterochronic interorgan signatures were due to SASPs secreted from the OCM-aged WAT and hepatogenic growth factors secreted from the YCM-exposed youthful WAT (BMP4[13], FGFs[14])."

<u>And anti-aging targeting starting at line 422:</u> "While not exhaustive, we tested multiple anti-gerontic strategies to demonstrate the value of our aging-on-a-chip system for discovery applications focusing on the adipocyte-MPS. Serum dilution, though a simple approach, proved highly effective in removing SASPs, thereby attenuating aging phenotypes and enhancing the efficacy of subsequent interventions. This finding aligns with the growing clinical interest in therapeutic plasma exchange (TPE) as an emerging rejuvenation modality[15]. Oxytocin treatment demonstrated some of the more promising anti-gerontic effects by simultaneously promoting lipolysis, reducing inflammation, and correcting visceral fat redistribution[16-18]. Inhibition of TGF-β signaling through its receptor subunit ALK5 also produced robust anti-gerontic effects, potentially due to its capacity to both promote adipogenesis and alleviate TGF-β/SMAD3-driven senescence. However, ALK5i treatment also resulted in the expansion of aged adipose progenitor populations based on increased LIFR in Fig.6c."

In addition to above mechanism, the feasibility of using a tissue as an aging prediction target differs substantially, with subcutaneous WAT (SAT) being far more practical than liver. Liver tissue is considerably less accessible, as intraperitoneal sampling is much more invasive and riskier compared to subcutaneous adipose sampling. Furthermore, liver biopsies are typically derived from sub-healthy donors, introducing biases toward disease-associated geno/phenotypes. As a result, the GTEx database contains only ~200 liver samples in the age range of 20-79, and the 20-29-year-old liver cohort (4 males and 3 females)

unexpectedly shows enrichment for inflammatory signatures, raising concerns about both the availability and reliability of these samples for machine-learning applications. This justification is now in line 369-372 in the new manuscript.

Despite these limitations, we trained a biological age prediction model for liver using GTEx data, applying the same machine learning framework used for WAT and developing sex-specific models. The male liver model reached an accuracy of 88%, predicting YCM liver-MPS as 57.8 years and OCM liver-MPS as 61.2 years. However, its performance was less robust than the corresponding male WAT model. Within this compromised model, knock-on liver aging groups failed to show distinct predicted ages (all 54.5 years), likely due to their aging hallmarks being less comprehensive and less altered compared to OCM-induced aging. The female liver model, though yielding a nominal accuracy of 100%, was even less reliable, given the very limited dataset of only 65 samples. It predicted YCM and OCM liver-MPS as 54.5 and 59.5 years, respectively, but with limited confidence. All of these sexual dimorphism result and considerations are included in the manuscript (Extended Data Fig. 4 and line 360-372)

7 • In Figure 5, each tested intervention should be independently validated in a simpler, less complex model to ensure robustness.

Heterochronic serum and senolytics have been extensively tested in standard tissue culture and in vivo animal model systems, for which we now provide references[27,28], and it was concludes by the filed that while somewhat informative, traditional static 2D tissue culture suffers from many inherent flaws, underperforming as compared to MPS/3D model [29-31]. To ensure the most rigorous validation of our MPS aging model, we demonstrated the geno- and phenotypic competency of our MPS against human primary tissues in our previous studies (Qi. et. al Small 2022, Groeger et. al. Nat. Comm. 2023, Qi. et. al Nat. Comm. 2024), and compared anti-gerontic interventions with the data from human primary tissues in line 422-436 in this study.

8 • The final section of Figure 5, which demonstrates the partial reversal of aging traits through exposure to young serum, represents a particularly compelling aspect of the study. Expanding this analysis would help justify the use of specific terminology (e.g., "rejuvenation") in the title and introduction.

Thank you for sharing our excitement. We have extended the section on rejuvenation strategies by adding CDKN2A/P16 knockdown and rapamycin treatment to inhibit mTOR signaling and characterized aging outcomes with additional assays (SA-beta-gal staining, P16 ELISA, DNA oxidation).

To be specific and conservative, we have changed term of “rejuvenation” to “anti-gerontic strategies” in the title and text.

9 • One of the most intriguing aspects of the model is its potential to reveal sex-specific differences in aging responses. However, this remains underexplored and should be more thoroughly considered.

We expanded our proteomics-based sex-specific analysis to adipose tissue and examined sex-specific transcriptomic profiles in the liver, now shown in Extended Data Fig.1, and further discussed the result (line 340-359).

Remarkably, using the same set of serum conditions, we were able to recapitulate tissue-specific sexual dimorphisms: inflammatory responses were higher in female adipose tissue and male liver, mirroring patterns observed in the GTEx human dataset. These findings suggest that sexually dimorphic inflammation arises not solely from inflammatory cytokines present in the serum, but also involves intrinsic, tissue-specific regulatory mechanisms.

Minor Points

• The manuscript would benefit from thorough editing, as some sentences appear fragmented, grammatical errors are present, and references to figures are occasionally unclear.

Multiple authors assisted in further editing of the manuscript.

• In the introduction, it would be beneficial to reference additional well-established interventions known to delay aging, such as caloric restriction, to provide broader context. Currently, the discussion emphasizes heterochronic blood exchange without acknowledging other established strategies.

Inspired by above, and given the metabolic similarities between adipose tissue aging and obesity, we may expect that interventions for weight loss by promoting catabolism, such as activation of the cAMP/PKA/AMPK pathways (e.g. metformin, semaglutide), or by inhibiting anabolic signaling, such as suppression of the insulin/PI3K/AKT/mTOR axis (e.g. caloric restriction, rapamycin) are considerable strategies to extend health span and may act synergistically with the approaches outlined above[19-22].

As we lacked direct correlations for these approaches in the MPS context and did not provide experimental evidence for the effectiveness of such anti-aging strategies, we now briefly mentioned and reference other intervention strategies including caloric restriction. (Line 457)

Please review the sentence in lines 203–205, as it may require clarification or revision.

We reviewed and corrected the sentence, and restructured whole discussion for better readability.

Reviewer #3 (Report for the authors (Required)):

Manuscript [nBME-25-0200] entitled “Aging in human microphysiological systems closely recreates the in vivo process with insights on rejuvenation” showed that a model indicating the aging transmission mechanism from adipose tissue to hepatocytes based on iPSC. This system can quickly simulate the aging process in vitro, that provided a new perspective for aging mechanisms and rejuvenation strategies. The authors also conducted massive omics analyses and revealed the changes of some aging-related key pathways and genes. However, there are several concerns that may significantly damp the overall quality and suitability for publication in nBME.

1. The authors have detected many biomarkers of aging including P16, SASP, PCK1 and SREBP1c, and evaluated by massive omics analysis. These changes have reported in previous studies (Carlos López-Otín,2023,Cell; Vall-Llaura N,2019, Redox Biol;). In addition, heterochronic parabiosis (Pálovics R,2022,Nature) and MPS (Ao Z, 2022,Adv Sci; Park S,2024, Aging Cell) have also reported in aging field.

Heterochronic parabiosis is not applicable to humans, and the published profiling of plasma factors is limited to post-fact correlations. This contrasts with the dynamic real-time changes of human tissue aging that we establish through human serum circulation and mechanistically explain. Moreover, no studies previously reported the induction of human aging on chip in 4 days, or male to female specifics, or key involved signaling networks, or the effects on the biological noise, or correspondence between on-chip and in-vivo biological age: our work is pioneering in all these regards.

We sincerely thank the reviewer for pointing us to valuable references on aging research in MPS, which we have cited in our updated manuscript (line 338). We have discussed them and added the summary table in our revised manuscript.

Publication	Cell source	Cell scalability (pluripotency)	Isogenic coculture	Sexual dimorphic aging	Modeling tissue types	Aging induction method	Aging hallmarks
Ao, Z., et. al. 2022. *Advanced Science*, *9*(27), p.2200475.	hESC-derived brain organoid and human primary monocytes	Partially	No	N/A	Brain-Immune	senescent immune cells from old donors	Gene expression, SASP secretion
Bersini, S.,et. al. 2020. Advanced Biosystems, 4(5), p.2000044.	Human primary endothelial and fibroblast	No	No	N/A	Vascular	Donor's natural aging, human serum for 14 and 25 days	Gene expression, immunostaining, transcriptomics, function of permeability
Pauty, J., et. al. 2021. Biomaterials Science, 9(1), pp.199-211.	HUVEC, and human primary fibroblasts	No	No	N/A	Vascular	Irradiation-induced senescent fibroblasts	Gene expression, immunostaining, SASP secretion, functions of permeability and contraction
Giza, S., et. al. 2022. Aging Cell, 21(7), p.e13650.	Human primary myogenic cells	No	N/A	N/A	Muscle	Donor's natural aging	Gene expression, myotube morphology, function of contraction
Budhathoki, S., et. al. 2022. *Cells Tissues Organs*, *211*(3), pp.348-359.	Rat immortalized myoblast cell line	Yes	N/A	N/A	Muscle	doxorubicin, hypoxia	Gene expression, DNA damage and ROS staining, immunostaining, nuclei and cell morphology
Lee Y., et al. 2020. Adv. Funct. Mater., 30, 2002924	Mouse immortalized lines of myoblasts and endothelial cells, primary myogenic cell	Yes	No	N/A	Muscle-Vascular	Mice serum, oxidative stress	Gene expression, immunostaining, SASP secretion, myotube morphology
Our study	hiPSC-derived adipocyte and hepatocytes	Yes	Yes	Yes	Fat-Liver	Human serum for 4 days, serum-induced senescent adipocytes	Gene expression, b-gal staining, immunostaining, DNA damage, SASP secretion, transcriptomics, glucose and lipid metabolism

2. Why does aging shift from fat aging to liver aging? What is the rationale for experimental design? Although there are some reports show the adipose tissueliver axis (Jamerson LE,2024, Antioxidants), the background information is unclear.

The background has been well documented in our previous publication (Lin et al, 2024, Nature Comm.), where the fat-liver-MPS has been demonstrated its capacity of studying liver inflammation, dysregulated glucose and lipid metabolism induced by the metabolites and cytokines (e.g. TNFa and adiponectin) released from the upstream proinflammatory fat chip.

This study followed the exact same rationale as suggested in our transcriptomics and de novo proteomics. The upstream aged-fat released excessive SASPs (majorly the inflammatory and immunoregulatory cytokines) to downstream liver, leading to aging phenotype. On the other hand, YCM-treated fat produced tremendous youthful growth factors (e.g. BMP4 & FGFs) to liver, promoting liver developments and functions.

We have added a schematic to clarify the integration. Moreover, extended multi-omics analysis of serum-induced and adipose-liver axis aging were performed, as shown in updated Fig.2, 5 and extended data figures 1,2,8.

3.There are some mistakes in the manuscript. For example, the number of key words in this manuscript is 13. Fig 1B showed the concentration is negative value.

Per Nature BME's author guidance, there is no need for "key words". Therefore, we deleted key word section in the revised manuscript.

Negative value of Fig.1B ELISA, this is due to our values are calculated based on a negative control, i.e. a chip without any serum treatment in circulatory milieu by (tested concentration – control concentration). A negative value in TNFa ELISA indicates the beneficial effect of young circulatory milieu, which is anti-inflammatory compared to the no serum control. We thank reviewer's reminding and have added more clarification in the Figure legend to avoid potential misunderstanding.

References in this response letter:

1 Buj, R., Leon, K. E., Anguelov, M. A. & Aird, K. M. Suppression of p16 alleviates the senescence-associated secretory phenotype. *Aging (Albany NY)* **13**, 3290 (2021).
2 Kim, D. *et al.* Old plasma dilution reduces human biological age: a clinical study. *Geroscience* **44**, 2701-2720 (2022).
3 Mehdipour, M. *et al.* Rejuvenation of three germ layers tissues by exchanging old blood plasma with saline-albumin. *Aging (Albany NY)* **12**, 8790 (2020).
4 Conboy, I. M. *et al.* Rejuvenation of aged progenitor cells by exposure to a young systemic environment. *Nature* **433**, 760-764 (2005).
5 Rebo, J. *et al.* A single heterochronic blood exchange reveals rapid inhibition of multiple tissues by old blood. *Nature communications* **7**, 13363 (2016).
6 Jeon, O. H. *et al.* Systemic induction of senescence in young mice after single heterochronic blood exchange. *Nature metabolism* **4**, 995-1006 (2022).
7 Liu, Y. *et al.* Application of bio-orthogonal proteome labeling to cell transplantation and heterochronic parabiosis. *Nature communications* **8**, 643 (2017).
8 Yue, Z. *et al.* Senescence-associated secretory phenotype and its impact on oral immune homeostasis. *Frontiers in Immunology* **13**, 1019313 (2022).
9 Wang, B., Han, J., Elisseeff, J. H. & Demaria, M. The senescence-associated secretory phenotype and its physiological and pathological implications. *Nature Reviews Molecular Cell Biology* **25**, 958-978 (2024).
10 Lee, M.-J. Transforming growth factor beta superfamily regulation of adipose tissue biology in obesity. *Biochimica et Biophysica Acta (BBA)-Molecular Basis of Disease* **1864**, 1160-1171 (2018).
11 Sánchez, A. *et al.* Transforming growth factor-β (TGF-β) and EGF promote cord-like structures that indicate terminal differentiation of fetal hepatocytes in primary culture. *Experimental cell research* **242**, 27-37 (1998).
12 Sánchez, A. & Fabregat, I. Growth factor-and cytokine-driven pathways governing liver stemness and differentiation. *World journal of gastroenterology: WJG* **16**, 5148 (2010).
13 Fan, J. *et al.* Bone morphogenetic protein-4 induced rat hepatic progenitor cell (WB-F344 cell) differentiation toward hepatocyte lineage. *J Cell Physiol* **220**, 72-81 (2009).
14 Padrissa-Altés, S. *et al.* Control of hepatocyte proliferation and survival by Fgf receptors is essential for liver regeneration in mice. *Gut* **64**, 1444-1453 (2015).
15 Fuentealba, M. *et al.* Multi-Omics Analysis Reveals Biomarkers That Contribute to Biological Age Rejuvenation in Response to Single-Blinded Randomized Placebo-Controlled Therapeutic Plasma Exchange. *Aging Cell*, e70103 (2025).
16 Szeto, A., Cecati, M., Ahmed, R., McCabe, P. M. & Mendez, A. J. Oxytocin reduces adipose tissue inflammation in obese mice. *Lipids in health and disease* **19**, 1-11 (2020).
17 Yuan, J., Zhang, R., Wu, R., Gu, Y. & Lu, Y. The effects of oxytocin to rectify metabolic dysfunction in obese mice are associated with increased thermogenesis. *Molecular and cellular endocrinology* **514**, 110903 (2020).
18 Cho, S. Y. *et al.* Oxytocin alleviates cellular senescence through oxytocin receptor-mediated extracellular signal-regulated kinase/Nrf2 signalling. *British Journal of Dermatology* **181**, 1216-1225 (2019).
19 McIntyre, R. L. *et al.* Pharmaceutical and nutraceutical activation of FOXO3 for healthy longevity. *Ageing research reviews* **78**, 101621 (2022).
20 Ortiz, G. U. & de Freitas, E. C. Semaglutide as a possible therapy for healthy aging: targeting the hallmarks of aging. *Ageing Research Reviews* **102**, 102582 (2024).
21 Yang, Y. *et al.* Metformin decelerates aging clock in male monkeys. *Cell* **187**, 6358-6378. e6329 (2024).
22 Miranda, A. M. *et al.* Selective remodelling of the adipose niche in obesity and weight loss. *Nature*, 1-11 (2025).
23 Ng, R., Sutradhar, R., Yao, Z., Wodchis, W. P. & Rosella, L. C. Smoking, drinking, diet and physical activity—modifiable lifestyle risk factors and their associations with age to first chronic disease. *International journal of epidemiology* **49**, 113-130 (2020).
24 Oh, H. S.-H. *et al.* Plasma proteomics links brain and immune system aging with healthspan and longevity. *Nature Medicine*, 1-9 (2025).
25 Ressler, S. *et al.* p16INK4A is a robust in vivo biomarker of cellular aging in human skin. *Aging cell* **5**, 379-389 (2006).

26 Coryell, P. R. *et al.* Autophagy regulates the localization and degradation of p16INK4a. *Aging cell* **19**, e13171 (2020).

27 Di Micco, R., Krizhanovsky, V., Baker, D. & d'Adda di Fagagna, F. Cellular senescence in ageing: from mechanisms to therapeutic opportunities. *Nature reviews Molecular cell biology* **22**, 75-95 (2021).

28 Kirkland, J. & Tchkonia, T. Senolytic drugs: from discovery to translation. *Journal of internal medicine* **288**, 518-536 (2020).

29 Carr, L. M., Mustafa, S., Care, A. & Collins-Praino, L. E. More than a number: incorporating the aged phenotype to improve in vitro and in vivo modeling of neurodegenerative disease. *Brain, Behavior, and Immunity* **119**, 554-571 (2024).

30 Mannarino, M. *et al.* Senolytic combination treatment is more potent than single drugs in reducing inflammatory and senescence burden in cells from painful degenerating IVDs. *Biomolecules* **13**, 1257 (2023).

31 Biju, T. S., Priya, V. V. & Francis, A. P. Role of three-dimensional cell culture in therapeutics and diagnostics: an updated review. *Drug delivery and translational research* **13**, 2239-2253 (2023).

<u>Point-by-point response to reviewers:</u>

Reviewer #1 (Report for the authors (Required)):

In this revised manuscript, the authors have addressed the major concerns of reviewers. It is suitable for publication on Nature Biomedical Engineering.

Reviewer #2 (Report for the authors (Required)):

The authors have done an excellent job in revising the manuscript and have fully addressed the concerns I raised in my initial review. The revisions are thorough, clearly explained, and strengthen the overall impact of the study. I sincerely thank the authors for the time, effort, and dedication they have invested in improving the manuscript, which was already of high quality.

We sincerely thank Reviewer #1, Reviewer#2, and our Editor for their insightful and constructive comments throughout the peer-review process. We truly believe that the quality and depth of this manuscript have been greatly enhanced by their professional and intellectual contributions. It is our honor to collaborate with them in advancing the science.

Reviewer #3 (Report for the authors (Required)):

After careful review, we believe that the research is not innovative enough to be published in Nature BME. The major concern lies in the limited functionality and translational relevance of the microphysiological system (MPS) presented.

We are sorry to hear that our manuscript did not meet your expectations. As your review did not include constructive suggestions for improvement, we have instead focused on addressing the points of disagreement in detail. For clarity and transparency, your comments have been numbered below for a point-by-point response:

1. While the system demonstrates the capability to culture and interconnect different tissue types, it lacks robust and validated functionality for detecting and analyzing key hallmarks of aging.

According to a much cited **review in Cell by López-Otín et al. (Cell, 2013; cited >17,500 times)** and the **updated review (Cell, 2023; cited >4,400 times),** twelve fundamental hallmarks define the biology of cellular aging: genomic instability, telomere attrition, epigenetic alterations, loss of proteostasis, deregulated nutrient sensing, mitochondrial dysfunction, cellular senescence, stem cell exhaustion, altered intercellular communication, disabled macroautophagy, chronic inflammation, and dysbiosis.

In our study, we validated **11 of 12 hallmarks**, excepting dysbiosis was beyond the scope of our model due to its tissue specificity. Although stem cell exhaustion is less directly relevant to mature adipocytes and hepatocytes, it was nevertheless reflected in our RNA-seq analysis as an enriched GO term associated with stem cell differentiation.

Importantly, **none of the current MPS-based aging studies**, including those cited in your previous comments and summarized in our comparative table (Supplementary Information) **that was included in the previous resubmission** have achieved validation of such a broad spectrum of aging hallmarks as demonstrated in our work.

Comprehensive evidence supporting the presence of these hallmarks in our system is summarized in the table below:

References	Aging Hallmarks	Validation in our study
Reviews by Carlos López-Otín, et al. on Cell in 2013 and on Cell in 2023	Genomic instability	DNA damage (Fig.1, Fig.5a, EDFig.2), aging noise (Fig.4b,5d),
	Telomere attrition	DNA damage (Fig.1), dysregulated telomere maintenance (Fig.5d, EDFig.5e),
	Epigenetic alterations	All transcriptomic alteration and noises
	Loss of proteostasis	Upregulated protein refolding GO terms (bioinformatic source data of ED. Fig7b), autophagy terms (EDFig.5b,c,d)
	Deregulated nutrient sensing	Insulin-desensitized glucose uptakes (Fig.1,2, 6), KEGG terms of insulin and PI3K-Akt signaling pathways (EDFig.1,2 5)
	Mitochondrial dysfunction	Dysregulated mitochondrial initiation, elongation and termination (Fig.4d, EDFig.5b
	Cellular senescence	SA-b-gal staining and P16 expression (Fig.1, 2, 6). KEGG terms of cellular senescence or aging (EDFig.2 and EDFig.5)
	Stem cell exhaustion	Not relevant/applicable in adipose and hepatic tissue. GO terms of stem cell differentiation (GO:0048863, bioinformatic source data of Fig.3c, EDFig.2c,5a)
	Altered intercellular communication	All data relevant to aging propagation from aged fat to liver MPS (Fig.2,5, EDFig.5 ,8)
Updated on Cell in 2023	Disabled macroautophagy	KEGG term of phagosome (EDFig.2g); KEGG term of noised autophagy (EDFig.5b,c,d);
	Chronic inflammation	All data support. EDFig.6 better distinguish it as chronic inflammation from M1 macrophage induced acute inflammation
	Dysbiosis	Not relevant/applicable in adipose and hepatic tissue

2. The claim that aging phenotypes—including senescence, oxidative damage, and metabolic dysfunction—can be reliably induced within just 4 days using serum from elderly donors appears physiologically overstated and is not sufficiently supported by the data. Such rapid induction does not adequately recapture the complex, multifactorial, and gradual process of human aging, thereby limiting the system's utility for mechanistic studies of aging or for screening anti-aging therapeutics.

Rapid induction is, in fact, **a well-established and distinctive advantage** of heterochronic blood exchange, which can robustly induce or reduce physiologic aging phenotypes within weeks to days, as demonstrated in many, highly cited research manuscripts by our colleagues and us over the past ~ 2 decades, e.g., [1-6]. These and other research papers and reviews on the field[7-9] show

that in rodents blood heterochronicity rapidly and robustly both, induces and rejuvenates complex, multifactorial physiologic aging processes. **Our technological platform innovatively moves this very interesting scientific paradigm from mice to human iPSC based MPS.**

We respectfully disagree with the reviewer's assertion that "rapid induction" is "physiologically overstated." Theoretically, **all classical aging induction approaches**, whether chemical, genetic, or environmental, operate on an accelerated timescale relative to human chronological aging. In the field, **the validity of an aging model is defined not by its temporal scale**, but by its ability to reproduce authentic aging phenotypes.

Our RNA-seq analyses and machine-learning-based aging prediction models consistently demonstrate that aging phenotypes in our MPS system **closely resemble natural aging patterns shown in the largest human database GTEx** for both adipose and liver tissues, and even more notably, they reproduce **sex-biased aging patterns**.

We also disagree with the statement that our system "does not adequately recapture complex, multifactorial aging." On the contrary, as detailed above table, our model successfully recreated **all aging hallmarks of our targeting tissues**, establishing it as a robust and physiologically relevant platform for **human** aging research.

Conclusively, this study establishes a paradigm of rapid, systemically controlled, human tissue aging process, exemplified by the human MPS age clock, which aligns with the chronological age of human serum donors.

3. Furthermore, the MPS does not represent a substantial technological advance over existing platforms due to the fact that there are many MPS devices available, and this study did not integrate the real-time aging monitoring indicator platform into the MPS device. The manuscript fails to introduce transformative innovations in design, readout capabilities, or physiological fidelity.

First, the **physiological fidelity** of our MPS platforms has already been **rigorously validated** in our previous studies (*Lin Qi et al., Small, 2022*; *Nature Communications, 2024*), demonstrating that the system faithfully reproduces physiologically relevant phenotypes **comparable to human primary tissues**. We recognize that this is a common and important question from reviewers indeed, which was also raised by the other two reviewers for this manuscript, and we have fully addressed it in our previous resubmission.

Second, the **objective of this work** is ***not*** to introduce a new microfluidic MPS design per se, but rather to **apply a recently-established and physiologically validated MPS to a new pre-clinical model: deliberate control of aging and rejuvenation of interconnectable human tissues**. Therefore, concerns regarding new types of MPS per se are not central to the current study's scope.

We noticed that the reviewer is asking for both translation and innovations in design. From a practical and translational standpoint, a device intended for broad use should adopt a straightforward and widely accessible design, ideally one that is already standardized and

familiar to most users. Consequently, highly novel designs may be less compatible with or even contradict to ease of translation and widespread implementation.

Instead, we wish to highlight the **key innovation** of this paper:

1. **Aging studies in adipose tissue** remain limited, and our work represents one of the most comprehensive and mechanistically detailed investigations to date.
2. This enabled us to pioneer the **first in vitro study of aging along the adipose–liver axis**, a connection that has been extremely challenging to examine *in vivo*. This represents a **major conceptual advancement**, providing new insights into how aging signals propagate from the initially aged tissue to the downstream organ.
3. We further demonstrate the successful **translation of heterochronic-based aging induction and rejuvenation from mice to human MPSs**, expanding its application to involve metabolism, senescence, and inflammaging, while standardizing assays and MPS configurations to **enable reproducible mechanistic studies** and the screening of longevity therapeutics.
4. A fully scalable aging MPS model based on **hiPSC sources**, offering strong potential for translation, and importantly, future studies of isogenic system on immune crosstalk without the risk of immune rejection, as showcased in our fat-macrophage MPS study (Lin Qi, et al. Small 2023, Nature Communication 2024).

We acknowledge that the phrase **"real-time"** appeared twice (in the Abstract and Discussion Summary) with only limited direct experimental validation of BONCAT de-novo proteomics on microfluidics. We have now **removed the two mentions** for accuracy.

We appreciate the reviewer's and editor's insightful suggestion regarding **monitoring indicators**. We have now included a discussion of this valuable point, for its "rapid" signature instead of "real-time" in the revised manuscript, as shown below:

> Starting from line 375:
> "Beyond transcriptome-based age prediction models, aging clock algorithms utilizing proteomics, metabolomics, and imaging modalities are currently under development[10]. Our system enables rapid monitoring of metabolite and cytokine dynamics through analysis of BONCAT de-novo proteomics on microfluidics, effluent medium and microscopy, facilitating continuous tracking of the aging process without disrupting MPS cultures. The integration of multiple indicators, including fatty acids, glucose, and SASP factors in the effluent medium, along with cellular morphology and intracellular lipid droplet dimensions obtained via imaging, provides orthogonal validation and enhances the precision of aging status assessment in live MPS."

We understand the reviewer's concerns and respect to editor's suggestion regarding **the limitation of aging induction by elderly donor serum**. We have now included a discussion of this valuable point in the revised manuscript, as shown below:

> Starting from line 452
> "One limitation of inducing aging through bloodborne factors is the variability of serum composition among individual donors. In our preliminary studies, we observed that this

variability was particularly pronounced in samples from regions with a high prevalence of obesity. To minimize such bias, we selected serum samples from sub-Saharan populations, where the average obesity rate remains below 15%. Similarly, other confounding factors, such as medication use, dietary habits, and lifestyle, may further influence the outcomes of aging induction. From a positive perspective, this variability provides an opportunity to investigate cohort-specific effects driven by geography, genetics and life-styles (e.g. caloric restriction, exercise, alcohol) in future work with larger donor pools and platforms with high experimental scalability. Given the practical limitations of human serum availability, our miniaturized and cost-efficient system established the key parameters of controlled human tissue aging on chip for such future explorations.

Overall, we hope that our novel aging-on-a-chip approach will empower research to further understanding of human aging, to provide mechanistic insights into heterochronic serum exchange, as well as the discovery and testing of anti-gerontic strategies in a pre-clinical model with high human relevance.

References for this response letter:

1 Conboy, I. M. *et al.* Rejuvenation of aged progenitor cells by exposure to a young systemic environment. *Nature* **433**, 760-764 (2005).

2 Villeda, S. A. *et al.* The ageing systemic milieu negatively regulates neurogenesis and cognitive function. *Nature* **477**, 90-94 (2011).

3 Rebo, J. *et al.* A single heterochronic blood exchange reveals rapid inhibition of multiple tissues by old blood. *Nature communications* **7**, 13363 (2016).

4 Jeon, O. H. *et al.* Systemic induction of senescence in young mice after single heterochronic blood exchange. *Nature metabolism* **4**, 995-1006 (2022).

5 Rando, T. A., Brunet, A. & Goodell, M. A. Hallmarks of stem cell aging. *Cell stem cell* (2025).

6 Rando, T. A. & Chang, H. Y. Aging, rejuvenation, and epigenetic reprogramming: resetting the aging clock. *Cell* **148**, 46-57 (2012).

7 Lagunas-Rangel, F. A. Aging insights from heterochronic parabiosis models. *npj Aging* **10**, 38 (2024).

8 Pálovics, R. *et al.* Molecular hallmarks of heterochronic parabiosis at single-cell resolution. *Nature* **603**, 309-314 (2022).

9 Ximerakis, M. *et al.* Heterochronic parabiosis reprograms the mouse brain transcriptome by shifting aging signatures in multiple cell types. *Nature Aging* **3**, 327-345 (2023).

10 Han, J.-D. J. The ticking of aging clocks. *Trends in Endocrinology & Metabolism* **35**, 11-22 (2024).

Manuscript [nBME-25-0200] entitled "Aging in human microphysiological systems closely recreates the in vivo process with insights on rejuvenation" showed that a model indicating the aging transmission mechanism from adipose tissue to hepatocytes based on iPSC. This system can quickly simulate the aging process *in vitro*, that provided a new perspective for aging mechanisms and rejuvenation strategies. The authors also conducted massive omics analyses and revealed the changes of some aging-related key pathways and genes. However, there are several concerns that may significantly damp the overall quality and suitability for publication in nBME.

1. The authors have detected many biomarkers of aging including P16, SASP, PCK1 and SREBP1c, and evaluated by massive omics analysis. These changes have reported in previous studies (Carlos López-Otín,2023,*Cell*; Vall-Llaura N,2019, *Redox Biol*;). In addition, heterochronic parabiosis (Pálovics R,2022,*Nature*) and MPS (Ao Z, 2022,*Adv Sci*; Park S,2024, *Aging Cell*) have also reported in aging field.

2. Why does aging shift from fat aging to liver aging? What is the rationale for experimental design? Although there are some reports show the adipose tissue-liver axis (Jamerson LE,2024, *Antioxidants*), the background information is unclear.

3. There are some mistakes in the manuscript. For example, the number of key words in this manuscript is 13. Fig 1B showed the concentration is negative value.
